# *CYP1B1*-*RMDN2* Alzheimer’s disease endophenotype locus identified for cerebral tau PET

**DOI:** 10.1038/s41467-024-52298-2

**Published:** 2024-09-20

**Authors:** Kwangsik Nho, Shannon L. Risacher, Liana G. Apostolova, Paula J. Bice, Jared R. Brosch, Rachael Deardorff, Kelley Faber, Martin R. Farlow, Tatiana Foroud, Sujuan Gao, Thea Rosewood, Jun Pyo Kim, Kelly Nudelman, Meichen Yu, Paul Aisen, Reisa Sperling, Basavaraj Hooli, Sergey Shcherbinin, Diana Svaldi, Clifford R. Jack, William J. Jagust, Susan Landau, Aparna Vasanthakumar, Jeffrey F. Waring, Vincent Doré, Simon M. Laws, Colin L. Masters, Tenielle Porter, Christopher C. Rowe, Victor L. Villemagne, Logan Dumitrescu, Timothy J. Hohman, Julia B. Libby, Elizabeth Mormino, Rachel F. Buckley, Keith Johnson, Hyun-Sik Yang, Ronald C. Petersen, Vijay K. Ramanan, Nilüfer Ertekin-Taner, Prashanthi Vemuri, Ann D. Cohen, Kang-Hsien Fan, M. Ilyas Kamboh, Oscar L. Lopez, David A. Bennett, Muhammad Ali, Tammie Benzinger, Carlos Cruchaga, Diana Hobbs, Philip L. De Jager, Masashi Fujita, Vaishnavi Jadhav, Bruce T. Lamb, Andy P. Tsai, Isabel Castanho, Jonathan Mill, Michael W. Weiner, for the Alzheimer’s Disease Neuroimaging Initiative (ADNI), Kwangsik Nho, Liana G. Apostolova, Kelley Faber, Martin R. Farlow, Tatiana Foroud, Paul Aisen, Reisa Sperling, Clifford R. Jack, William J. Jagust, Susan Landau, Ronald C. Petersen, Prashanthi Vemuri, Oscar L. Lopez, Michael W. Weiner, Andrew J. Saykin, the Department of Defense Alzheimer’s Disease Neuroimaging Initiative (DoD-ADNI), Paul Aisen, Ronald C. Petersen, Michael W. Weiner, the Anti-Amyloid Treatment in Asymptomatic Alzheimer’s Study (A4 Study) and Longitudinal Evaluation of Amyloid Risk and Neurodegeneration (LEARN), Jared R. Brosch, Martin R. Farlow, Paul Aisen, Reisa Sperling, Sergey Shcherbinin, Clifford R. Jack, Colin L. Masters, Elizabeth Mormino, Rachel F. Buckley, Keith Johnson, Oscar L. Lopez, Tammie Benzinger, the Australian Imaging, Biomarker & Lifestyle Study (AIBL), Simon M. Laws, Colin L. Masters, Tenielle Porter, Christopher C. Rowe, Andrew J. Saykin

**Affiliations:** 1 Center for Neuroimaging, Department of Radiology and Imaging Sciences, Indiana University School of Medicine, Indianapolis, USA; 2 Center for Computational Biology and Bioinformatics, Indiana University School of Medicine, Indianapolis, USA; 3 Indiana Alzheimer’s Disease Research Center, Indiana University School of Medicine, Indianapolis, USA; 4 Department of BioHealth Informatics, Indiana University, Indianapolis, USA; 5 Department of Neurology, Indiana University School of Medicine, Indianapolis, USA; 6 Department of Medical and Molecular Genetics, Indiana University School of Medicine, Indianapolis, USA; 7 National Centralized Repository for Alzheimer’s Disease and Related Dementias, Indiana University School of Medicine, Indianapolis, USA; 8 Department of Biostatistics, Indiana University School of Medicine, Indianapolis, USA; 9 Department of Neurology, Keck School of Medicine, University of Southern California, San Diego, USA; 10 Department of Neurology, Massachusetts General Hospital, Harvard Medical School, Boston, USA; 11 Eli Lilly and Company, Indianapolis, USA; 12 Department of Radiology, Mayo Clinic, Rochester, USA; 13 UC Berkeley Helen Wills Neuroscience Institute, University of California - Berkeley, Berkeley, USA; 14 Genomics Research Center, AbbVie, North Chicago, USA; 15 CSIRO Health and Biosecurity, Melbourne, Australia; 16 Department of Molecular Imaging & Therapy, Austin Health, Heidelberg, Australia; 17 Centre for Precision Health, School of Medical and Health Sciences, Edith Cowan University, Joondalup, Australia; 18 Florey Institute of Neuroscience and Mental Health and The University of Melbourne, Parkville, Australia; 19 Department of Psychiatry, University of Pittsburgh School of Medicine, Pittsburgh, USA; 20 Vanderbilt Memory & Alzheimer’s Center, Vanderbilt University Medical Center, Nashville, USA; 21 Vanderbilt Genetics Institute, Vanderbilt University Medical Center, Nashville, USA; 22 Department of Neurology & Neurological Sciences, Stanford University, Stanford, USA; 23 Department of Radiology, Massachusetts General Hospital, Harvard Medical School, Boston, USA; 24 Center for Alzheimer’s Research and Treatment, Department of Neurology, Brigham and Women’s Hospital, Harvard Medical School, Boston, USA; 25 Department of Neurology, Mayo Clinic, Rochester, USA; 26 Department of Neurology, Mayo Clinic, Jacksonville, USA; 27 Department of Neuroscience, Mayo Clinic, Jacksonville, USA; 28 Department of Human Genetics, University of Pittsburgh, Pittsburgh, USA; 29 Department of Neurology, University of Pittsburgh School of Medicine, Pittsburgh, USA; 30 Department of Neurological Sciences, Rush Medical College, Rush University, Chicago, USA; 31 Department of Psychiatry, Washington University, St. Louis, USA; 32 Department of Radiology, Washington University School of Medicine, St. Louis, USA; 33 NeuroGenomics and Informatics Center, Washington University School of Medicine, St. Louis, USA; 34 Center for Translational and Computational Neuroimmunology, Department of Neurology and Taub Institute for Research on Alzheimer’s Disease and the Aging Brain, Columbia University Irving Medical Center, New York, USA; 35 Stark Neuroscience Research Institute, Indiana University School of Medicine, Indianapolis, USA; 36 Wu Tsai Neurosciences Institute, Stanford University School of Medicine, Stanford, USA; 37 Department for Clinical and Biomedical Sciences, University of Exeter Medical School, University of Exeter, Exeter, UK; 38 Department of Pathology, Beth Israel Deaconess Medical Center, Harvard Medical School, Boston, USA; 39 Departments of Radiology, Medicine, and Psychiatry, University of California-San Francisco, San Francisco, USA; 40 Department of Veterans Affairs Medical Center, San Francisco, USA

**Keywords:** Genome-wide association studies, Alzheimer's disease

## Abstract

Determining the genetic architecture of Alzheimer’s disease pathologies can enhance mechanistic understanding and inform precision medicine strategies. Here, we perform a genome-wide association study of cortical tau quantified by positron emission tomography in 3046 participants from 12 independent studies. The *CYP1B1*-*RMDN2* locus is associated with tau deposition. The most significant signal is at rs2113389, explaining 4.3% of the variation in cortical tau, while *APOE4* rs429358 accounts for 3.6%. rs2113389 is associated with higher tau and faster cognitive decline. Additive effects, but no interactions, are observed between rs2113389 and diagnosis, APOE4, and amyloid beta positivity. *CYP1B1* expression is upregulated in AD. rs2113389 is associated with higher *CYP1B1* expression and methylation levels. Mouse model studies provide additional functional evidence for a relationship between *CYP1B1* and tau deposition but not amyloid beta. These results provide insight into the genetic basis of cerebral tau deposition and support novel pathways for therapeutic development in AD.

## Abstract (web summary)

A multi-cohort genome-wide association study of tau PET, a brain imaging-based marker of Alzheimer’s disease, identifies a *CYP1B1*-*RMDN2* locus as associated with higher tau and faster cognitive decline. These results suggest a new genetic contribution to cerebral tau and target for Alzheimer’s disease research.

## Introduction

Alzheimer’s disease (AD) is a neurodegenerative disease featuring amyloid-beta (Aβ) plaques and neurofibrillary tau tangles^1^. Aβ and tau measurements using positron emission tomography (PET) are common in research (i.e., amyloid/tau/neurodegeneration (A/T/N))^2^.

Genetic factors conferring susceptibility to or protection from AD are important for identifying biological pathways for drug development and personalized medicine^3^. Large-scale genome-wide association studies (GWAS) using case-control designs have identified risk genes in immune, tau, Aβ, lipid, and other pathways^4,5^. The strongest AD genetic risk locus is *APOE* (apolipoprotein E) ε4 (*APOE4*)^6^. Large case-control studies are often limited because participant neuropathology is unknown.

Endophenotype studies complement case-control studies by testing genetic variants against disease pathology^7^. Studies have assessed genetic predictors of Aβ PET measures^8–13^. Most genetic studies of tau have utilized cerebrospinal fluid (CSF) tau measures due to non-availability of large tau PET datasets^14^. One study investigated the association of [^18^F]flortaucipir PET with *BIN1*, finding an association between a known *BIN1* risk single nucleotide polymorphism (SNP; rs744373) and greater tau^15^. Another performed a GWAS on tau PET endophenotypes and identified two genetic loci (*PPP2R2B* and *IGF2BP3*), but a modest sample size (*n* = 754) and no replication sample^16,17^. Guo et al. performed a GWAS on tau PET (*n* = 543) and identified two genetic loci (*ZBTB20* and *EYA4*) associated with elevated tau accumulation and worse clinical performance^18^.

Here, we perform the largest GWAS of PET-based cortical tau to date (*n* = 3046). We include data from twelve independent cohorts. We also assess the relationship of the top SNP with cognitive decline and additive and interaction effects with diagnosis, *APOE ε4* status, and Aβ positivity. We map topographic distribution of the top variant effect on voxel-wise tau deposition. We perform a gene-set enrichment analysis, assess gene expression levels in human brain tissue and single-nucleus RNA-Seq data, map the expression of the top genes in the Allen Human Brain Atlas, and perform methylation and expression quantitative trait loci (eQTL) analyses. Finally, we investigate expression levels of the top gene in tau and Aβ mouse models^19–21^.

## Results

### Genome-wide association analysis (GWAS)

**Fig. 1:**
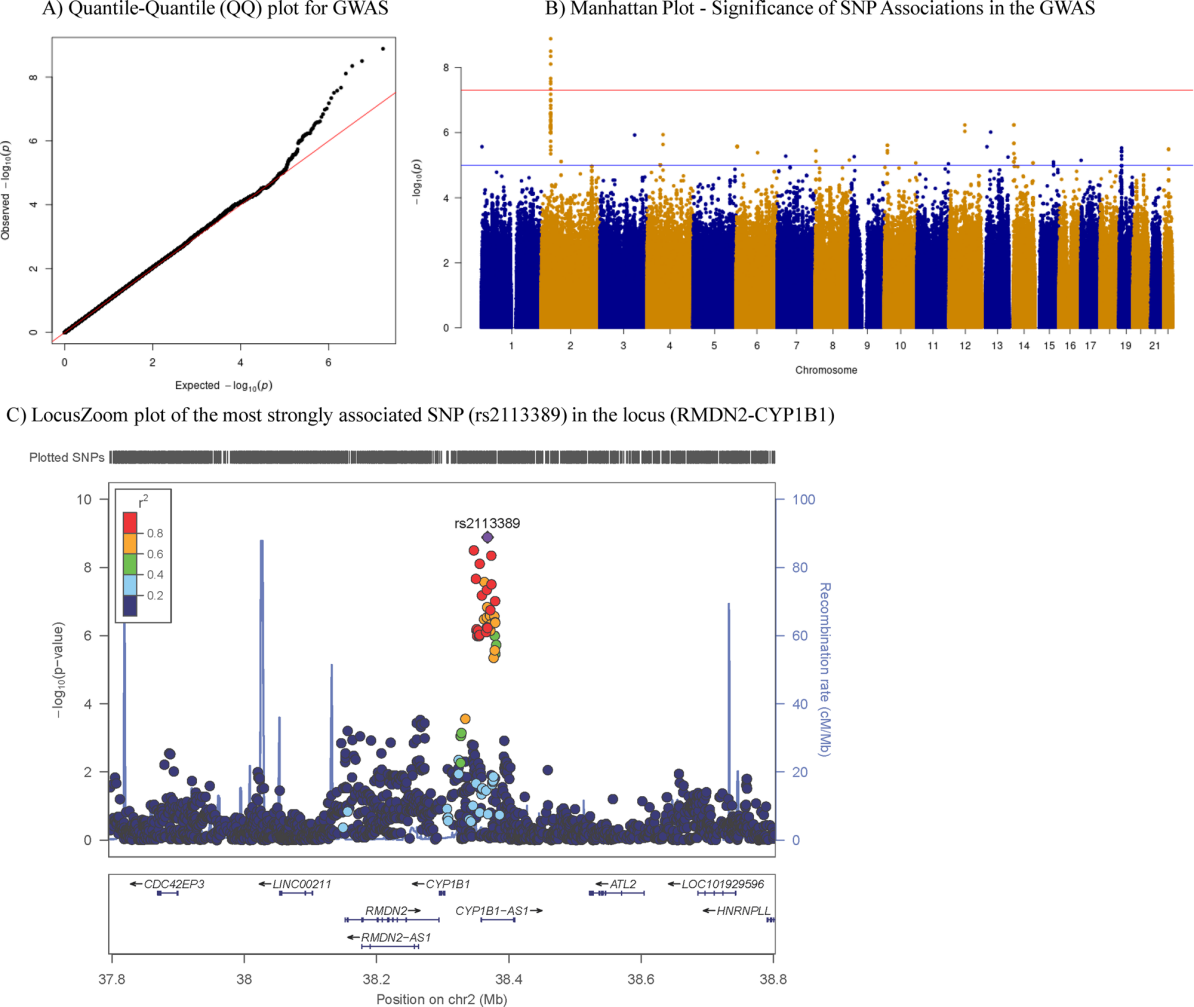
Results of Discovery GWAS for cortical tau deposition. Quantile-quantile (QQ) (**A**), Manhattan (**B**), and LocusZoom (**C**) plots of genome-wide association study (GWAS) results from seven discovery cohorts (*N* = 1446) using a linear regression model with age, sex, two principal component (PC) factors from population stratification, *APOE4* status, and diagnosis as covariates are shown. The genomic inflation factor is λ = 1.025 in the Manhattan plot (**B**), the horizontal blue and red lines represent the -log_10_(10^−5^) and -log_10_(5.0 × 10^−8^) threshold levels, respectively. Two single nucleotide polymorphisms (SNPs) on chromosome 2 showed highly significant ( < 5.0 × 10^−8^) associations with cerebral tau deposition. The regional association plot (**C**) for the locus that passed genome-wide significance shows the region around the most significant SNP (rs2113389) at the *RMDN2-CYP1B1* locus. SNPs were plotted based on their GWAS −log_10_ *p*-values and genomic position. The red color scale of *r*^2^ values was used to label SNPs based on their degree of linkage disequilibrium with the most significant SNP. Recombination rates calculated from 1000 Genomes Project reference data are also displayed in a blue line corresponding to the right vertical axis. *Note: cerebral tau endophenotype measured as an inverse normal transformed variable of cortical tau SUVR*.

Meta-analyzed GWAS results from seven discovery cohorts (*n* = 1446) are shown as quantile-quantile (Fig. 1A) and Manhattan (Fig. 1B) plots. No systematic *p*-value inflation was found (genomic inflation factor λ = 1.025; Fig. 1A). We identified a genome-wide significant association of cortical tau with a novel locus at 2p22.2 (Fig. 1B), with two SNPs reaching genome-wide significance (*p*-value ≤ 5 × 10^−8^). The strongest associated SNP is rs2113389, which was directly genotyped. The other SNP (rs918804) is in strong linkage disequilibrium (LD, *r*^2^ = 0.91 and D’ = 0.95). rs2113389 is located on 2p22.2 between *RMDN2*, *CYP1B1*, and non-coding RNA, *CYP1B1-AS1* (Fig. 1C). The minor allele T of rs2113389 (MAF = 0.146) was associated with higher tau (Z score = 5.68; *p*-value = 1.37 × 10−^8^; Heterogeneity *I*^2^ = 27.8; Heterogeneity *p*-value = 2.17 × 10−^1^). A replication meta-analysis in five additional cohorts (*n* = 1600) showed that the significant SNPs (rs2113389 and rs918804) in the discovery stage were replicated with the same association direction (Z Score=3.83, *p*-value = 1.26 × 10−^4^, Heterogeneity *I*^2^ = 52.0, Heterogeneity *p*-value = 8.02  × 10−^2^; Z-score = −2.97, *p*-value = 2.97 × 10^−3^, Heterogeneity *I*^2^ = 59.5; Heterogeneity *p*-value = 5.99 × 10^−2^, respectively; Supplementary Fig. 1). ~4.3% of the estimated proportional variation in cortical tau in ADNI is explained by rs2113389 and *APOE4* (rs429358).

### Association of rs2113389 genotype with regional and global tau

**Fig. 2:**
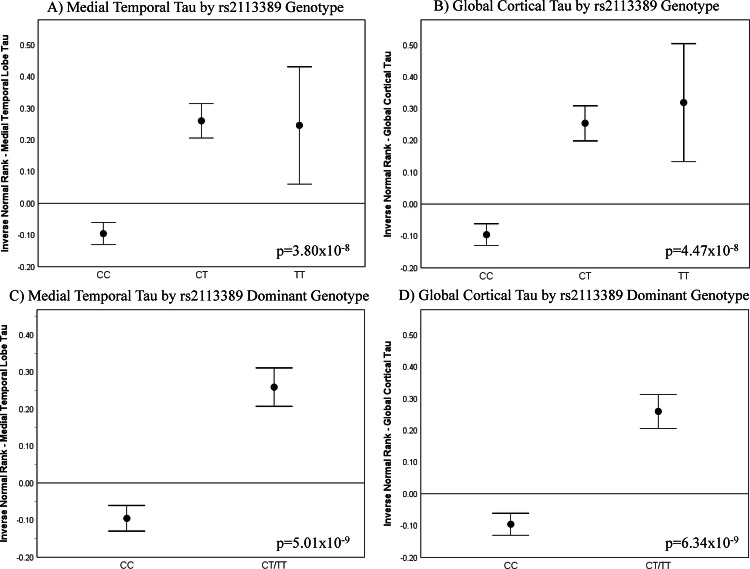
Association of the most significant SNP (rs2113389) at the *RMDN2-CYP1B1* locus with regional and global cortical tau burden. Using an additive model, the minor allele (T) of rs2113389 is associated with higher tau deposition across participants, with both rs2113389 CT and TT individuals showing significantly greater medial temporal lobe (MTL; **A**) and cortical (**B**) tau deposition than rs2113389 CC individuals. Similar results are seen using a dominant model. Specifically, individuals with one or more minor alleles of rs2113389 show significantly greater tau deposition in the medial temporal lobe (**C**) and cortex (**D**) than rs2113389 CC individuals. One-way ANCOVA models are used with rs2113389 genotype as the independent variable, covaried for age, sex, Aβ positivity, *APOE4* carrier status, and diagnosis. Plots represent mean ± standard error of the mean. Panels include 1,161 individuals (for **A**, **B**, 834 CC, 300 CT, 27 TT); for (**C**,**D**), 834 CC, 327 CT/TT). Source data are provided as a Source Data file. Aβ amyloid-beta; ANCOVA analysis of covariance; APOE apolipoprotein E; MTL medial temporal lobe; SUVR standardized uptake value ratio. *Note: tau measured as an inverse normal transformed variable of medial temporal and cortical tau SUVR*.

Figure 2 shows that both additive (Fig. 2A,B) and dominant models (Fig. 2C,D) demonstrated higher MTL and cortical tau deposition in rs2113389 minor allele (T) carriers. Similar results were observed when stratified by sex (Supplementary Figs. 2,3) and when using SUVR values rather than those with rank-based inverse normal transformation (Supplementary Fig. 4).

### Interaction of rs2113389 genotype with variables of interest

**Fig. 3:**
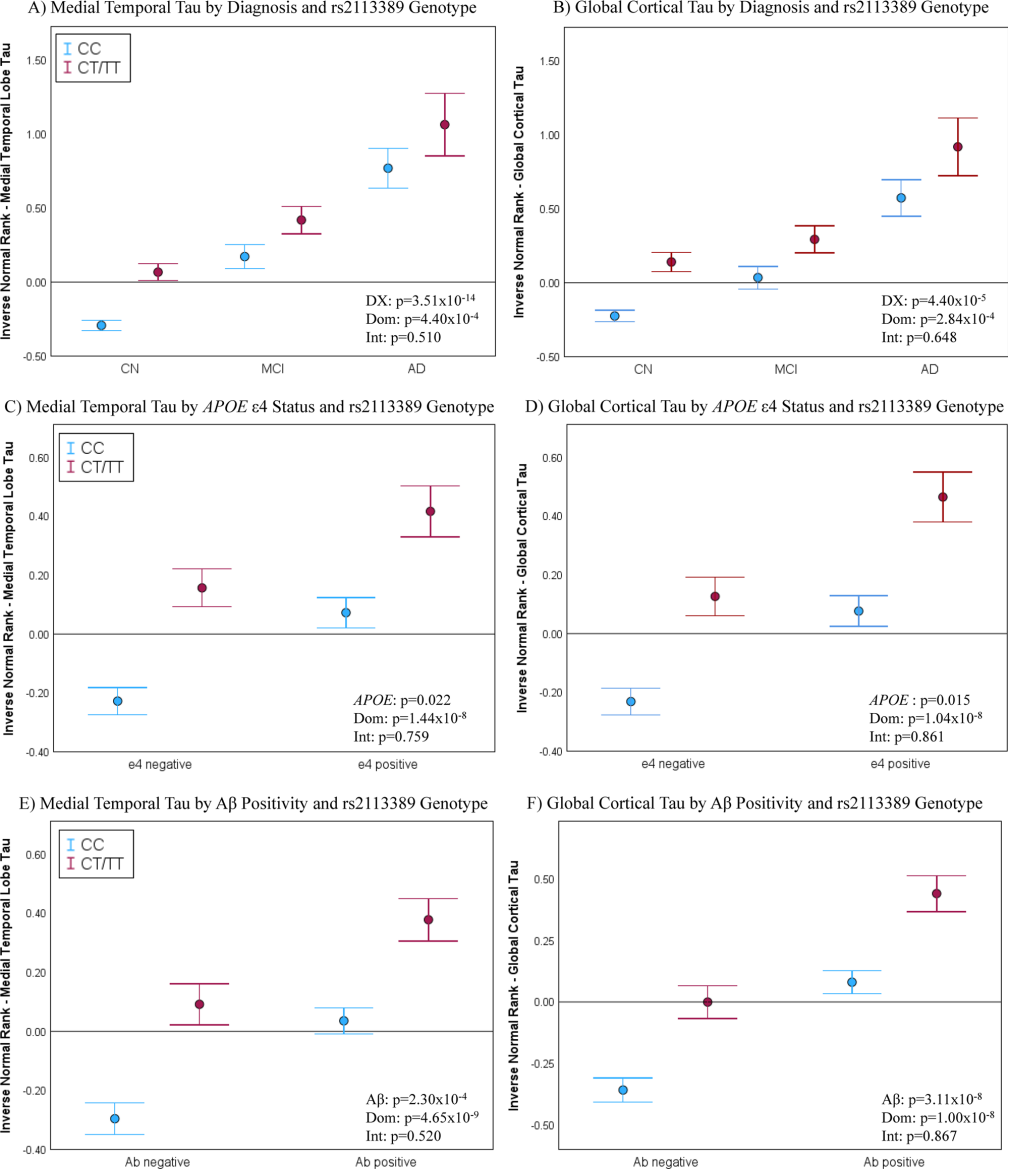
Interaction effect of the most significant SNP (rs2113389) at the *RMDN2-CYP1B1* locus with diagnosis, *APOE ε4* carrier status, and Aβ positivity on regional and cortical tau deposition. Both diagnosis and rs2113389 dominant genotype are significantly associated with medial temporal lobe (MTL; **A**) and cortical (**B**) tau deposition. *APOE4* carrier status and rs2113389 dominant genotype are significantly associated with MTL (**C**) and cortical (**D**) tau deposition. Significant effects of both Aβ positivity and rs2113389 dominant genotype on MTL (**E**) and cortical (**F**) tau deposition are observed. Two-way ANCOVA models, covaried for age, sex, as well as diagnosis, *APOE4* carrier status, and Aβ positivity where appropriate, are used. Plots are displayed as mean + /−standard error of the mean. Panels (**A**) and (**B**) include 1161 participants (568 CN-CC, 222 CN-CT/TT, 195 MCI-CC, 75 MCI-CT/TT, 71 AD-CC, 30 AD-CT/TT); panels (**C**) and (**D**) include 1161 participants (468 *APOE4*-/CC, 199 *APOE4*-/CT/TT, 366 *APOE4* + /CC, 128 *APOE4* + /CT/TT); panels (**E**) and (**F**) include 1154 participants (338 Aβ-/CC, 131 Aβ-/CT/TT, 491 Aβ + /CC, 194 Aβ + /CT/TT). Source data are provided as a Source Data file. Aβ amyloid-beta; AD Alzheimer’s disease; ANCOVA analysis of covariance; APOE apolipoprotein E; CN cognitively normal; DX diagnosis; Dom rs2113389 dominant genotype (CC vs. CT/TT); Int. interaction; MCI mild cognitive impairment; MTL medial temporal lobe; SUVR standardized uptake value ratio. *Note: tau measured as an inverse normal transformed variable of medial temporal and cortical tau SUVR*.

Main effects of diagnosis and rs2113389 genotype were observed but no interaction effect (Fig. 3A,B). As the pattern of the *RMDN2-CYP1B1* association is similar across diagnoses, this effect is not being fully driven by MCI/AD patients. The effect was similar in both males and females (Supplementary Fig. 5). Main effects, but no interaction effect, for rs2113389 genotype and *APOE4* were also observed (Fig. 3C,D). The sex-stratified analysis showed similar results in both males and females (Supplementary Fig. 6). Finally, main effects of Aβ positivity and rs2113389 genotype, but no interaction effect were observed (Fig. 3E,F). In the sex-stratified analysis, males and females showed similar results (Supplementary Fig. 7), except for an interaction effect of Aβ positivity and rs2113389 genotype on MTL tau deposition in females (Supplementary Fig. 7C). Similar results were also observed using SUVR values rather than the rank-based inverse normal transformed values (Supplementary Fig. 8).

### Voxel-wise association of rs2113389 genotype with tau

**Fig. 4:**
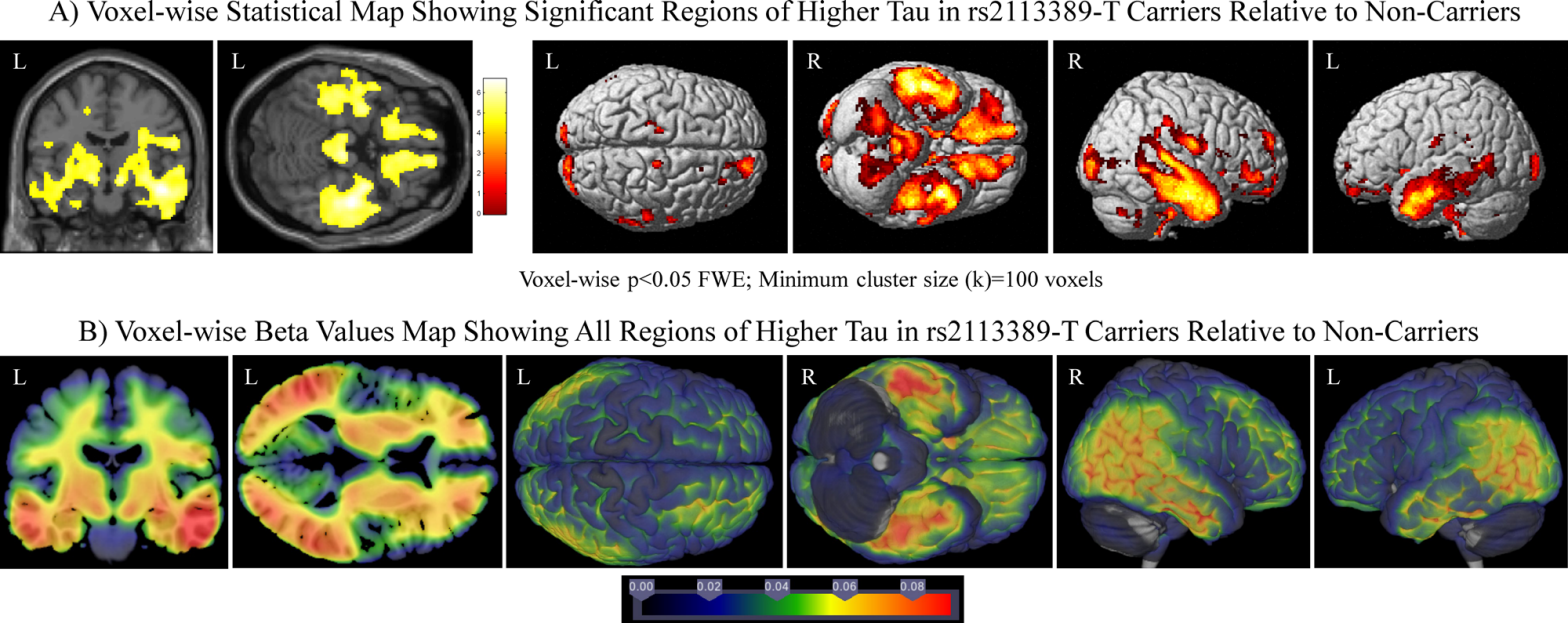
Voxel-wise analysis and visualization of the effect of rs2113389 dominant genotype on tau deposition. **A** Widespread regions of association between rs2113389 dominant genotype and tau deposition are observed in the inferior frontal, parietal, and medial and lateral temporal lobes, such that those with one or more minor alleles (T) at rs2113389 show greater tau deposition than CC rs2113389 individuals. Images are displayed at a voxel-wise threshold of *p* < 0.05 with family-wise error correction for multiple comparisons and a minimum cluster size (k) = 100 voxels. **B** Beta-value maps show widespread regions of higher tau deposition in rs2113389-T carriers relative to non-carriers. Specifically, temporal, parietal, and frontal lobe tau is greater in minor allele carriers than non-carriers. A one-way ANCOVA model is used, covaried for age, sex, diagnosis, *APOE4* carrier status, and Aβ positivity. Analyzes include 1154 individuals (829 CC, 325 CT/TT). Aβ amyloid-beta; ANCOVA analysis of covariance; APOE apolipoprotein E.

A voxel-wise analysis of the effect of rs2113389 (voxel-wise *p* < 0.05 (FWE corrected), minimum cluster size (k) = 100 voxels; Fig. 4 and Supplementary Fig. 9) evaluated the topographic pattern of the association. In the dominant model, rs2113389 minor allele carriers (CT or TT; *n* = 327) demonstrated greater tau than rs2113389 CC individuals (*n* = 834; Fig. 4A). Beta-value maps supported the statistical map, showing widespread areas where rs2113389-T carriers show higher tau than non-carriers (Fig. 4B). Using an additive model, rs2113389 CT individuals (*n* = 300) showed higher tau than CC individuals (*n* = 834) in the temporal, parietal, and frontal lobes (Supplementary Fig. 9A), while rs2113389 TT (*n* = 27) showed a focal region of higher frontal tau relative to CC individuals (Supplementary Fig. 9B). Beta-value maps revealed rs2113389 CT individuals showing higher temporal and parietal tau relative to rs2113389 CC individuals (Supplementary Fig. 9C). rs2113389 TT individuals showed widespread higher tau relative to rs2113389 CC individuals, especially in the frontal lobe (Supplementary Fig. 9D). Finally, the beta-values map shows that rs2113389 TT homozygotes show higher frontal tau than rs2113389 CT heterozygotes (Supplementary Fig. 9E), although this did not reach statistical significance.

### Association of rs2113389 genotype with CSF tau biomarkers

In addition to the findings with PET, rs2113389 genotype was associated with CSF levels of both total tau and phosphorylated tau 181 (pTau181), with the rs2113389 T-allele associated with higher levels of CSF total tau and pTau181 both in the additive model (Supplementary Fig. 10A,B) and dominant model (Supplementary Fig. 10C,D). We reviewed the GWAS summary statistics from two large-scale GWAS for CSF biomarkers^14,22^. rs1478361 was associated with CSF total-tau levels but not CSF p-Tau levels. rs1478361, which is in strong LD with rs2113389 (*r*^2^ = 0.96 and D’ = 1.00), was associated with CSF total tau levels (*n* = 3,076; β = 0.0176; *p*-value = 0.0295)^14^. Within the *CYP1B1* locus, the most significant SNPs for CSF p-Tau levels were rs12463523 (*p*-value = 0.0026) from the Deming et al. paper^14^ and rs9341266 (*p*-value = 0.0029) from the Jansen et al. paper^22^.

### Pathway analysis

When gene ontology (GO) terms were considered, 480 gene-sets were significant after correction for multiple testing. GO for cell-cell adhesion was the most significant pathway identified (Supplementary Table 13A). GO terms for MHC protein complex, postsynaptic density, regulation of synaptic transmission, and calcium ion transport were also significant. For the KEGG pathway, 44 gene-sets were significant, including cell adhesion molecules, calcium signaling pathways, and axon guidance (Supplementary Table 13B). GO terms for several pathways containing genes near the *CYP1B1* locus were significant, including those that regulate reactive oxygen species, metabolic processes, monooxygenase activity, Golgi organization, and endoplasmic reticulum organization, as well as the KEGG pathway for steroid hormone biosynthesis.

### Gene expression analysis and eQTL analysis

**Fig. 5:**
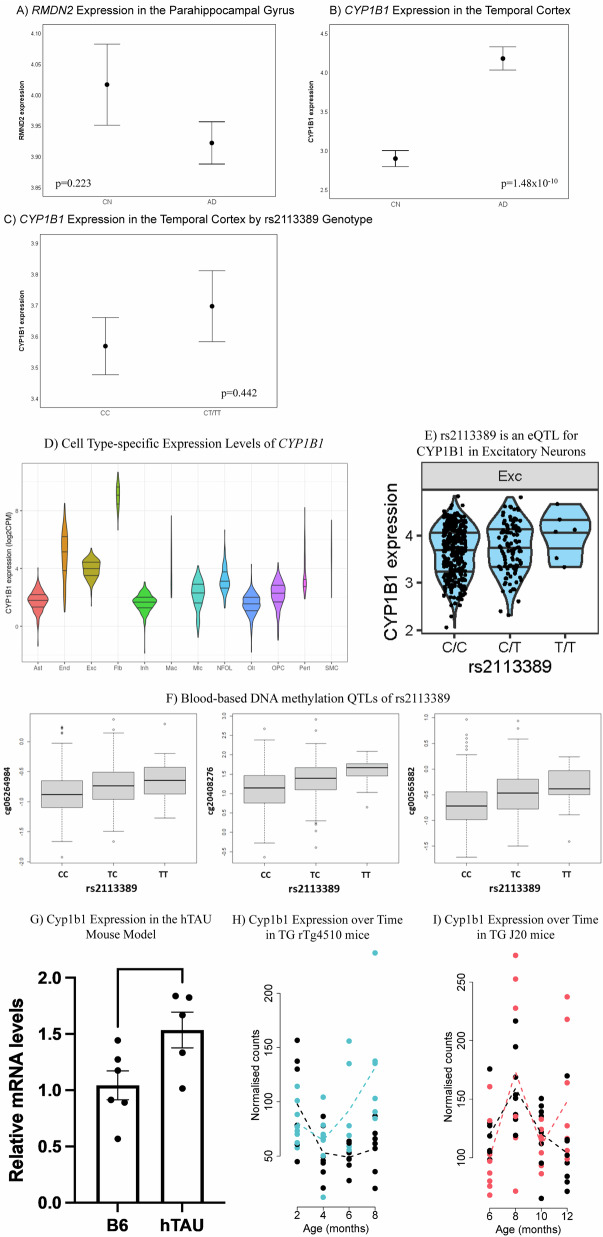
Gene expression analysis of *RMDN2* and *CYP1B1* and expression quantitative trait locus (eQTL) and DNA methylation QTL (meQTL) analysis of rs2113389. AD patients show downregulated expression of *RMDN2* in the parahippocampal gyri (**A**) and upregulated expression of *CYP1B1* in the temporal cortex (**B**) relative to CN using brain tissue-based RNA-Seq data from the AMP-AD project (Panel (**A**), *n* = 135 (26 CN, 109 AD); Panel (**B**), *n* = 151 (71 CN, 80 AD)). **C** In an eQTL analysis, the identified SNP (rs2113389) is associated with *CYP1B1* expression levels in the temporal cortex (*n* = 257 (188 CC, 69 CT/TT)). One-way ANCOVA models are used in Panels (**A**–**C**), and plots represent the mean ± standard error of the mean. Source data are provided for panels (**A**–**C**) as a Source Data file. Cell type-specific expression levels (**D**) and eQTL in the excitatory neuron (**E**) of *CYP1B1* gene (*N* = 424) are shown. In (**D**), the x-axis is cell types in ROSMAP DLPFC single-nucleus RNA-Seq data. The y-axis is the log_2_ of counts per million mapped reads (CPM) of *CYP1B1* gene. Expression levels are computed at the donor level by aggregating cells from the same donor. Rare cell types are observed only in a small fraction of donors. Areas of violin plots are scaled to the number of donors. Fibroblasts (Fib) has the highest expression of *CYP1B1* gene. Among major cell types, excitatory neurons (Exc) has the highest expression. In (**E**), the minor allele (T) of rs2113389 is associated with higher cell type-specific *CYP1B1* expression levels in the excitatory neuron (*p*-value = 0.035). **F** DNA methylation QTL analysis (*cis*-meQTL) of rs2113389 with CpGs in *CYP1B1* measured in blood samples from 634 ADNI participants demonstrate three CpGs, located in the *CYP1B1* gene body region, as significantly associated with rs2113389 (*p*-value = 7.04 × 10^−8^, 5.43 × 10^−9^, and 4.73 × 10^−12^, respectively). **G** *Cyp1b1* expression (relative mRNA expression levels by qPCR) is increased in the cortex of 6-month-old hTAU mice consistent with our findings in human LOAD (*p*-value = 0.038). The error bars represent the standard error of the mean. **H** *Cyp1b1* expression (normalized RNA-Seq read counts) significantly changes with time (genotype*age) in TG rTg4510 mice, suggesting *Cyp1b1* is associated with disease progression in the rTg4510 model. **I** *Cyp1b1* expression (normalized RNA-Seq read counts) does not change with time (genotype*age) in J20 mice, suggesting that *Cyp1b1* is not associated with amyloid pathology progression. AD Alzheimer’s disease; ADNI Alzheimer’s Disease Neuroimaging Initiative; AMP-AD Accelerating Medicines Partnership-AD; ANCOVA analysis of covariance; *cis*-meQTL DNA methylation quantitative trait loci; CN cognitively normal; CpG cytosines followed by guanine residues; CPM counts per million; DLPFC dorsolateral prefrontal cortex; DNA Deoxyribonucleic acid; eQTL expression quantitative trait loci; Exc excitatory neurons; Fib fibroblasts; hTAU humanized tau; ROSMAP Religious Orders Study/Memory and Aging Project; RNA-Seq Ribonucleic acid sequencing; SNP single nucleotide polymorphism.

Our genome-wide gene-based association analysis identified two protein coding genes (*CYP1B1* (corrected *p*-value = 0.040)), *RMDN2* (corrected *p*-value = 0.040)), and one non-coding RNA (*CYP1B1-AS1* (corrected *p*-value = 0.040)) associated with tau. Then, our Allen Human Brain Atlas visualization showed that *CYP1B1* was expressed across the whole brain, especially in the insula, orbitofrontal cortex, and temporal lobe. *RMDN2* was also expressed throughout the brain, especially the temporal lobe, visual cortex, frontal and posterior default mode network regions, and sensorimotor cortex (Supplementary Fig. 11). Processed bulk RNA-Seq data from 1917 samples downloaded from the AMP-AD Knowledeg Portal^23–26^ was evaluated for these genes. Differential expression of *RMDN2* was seen in the parahippocampal gyrus (*p*-value = 0.004; Fig. 5A), with down-regulation in AD. *CYP1B1* demonstrated differential expression in the temporal cortex (*p*-value = 0.001; Fig. 5B), with upregulation in AD. In eQTL analysis, the rs2113389 was associated with *CYP1B1* expression levels in the temporal cortex, but not with *RMDN2* expression. Specifically, the rs2113389 T-allele was associated with higher temporal *CYP1B1* expression (β = 0.25; *p*-value = 0.02; Fig. 5C). Finally, the rs2113389 T-allele was associated with higher *CYP1B1* expression levels in blood from the eQTLGen consortium database (*n* = 31,684; Z Score=24.93; *p*-value = 3.6 × 10^−137^).

### Cell type-specific expression and eQTL analysis of CYP1B1

Single-cell expression of *CYP1B1* in ROSMAP single-nucleus RNA-Seq data from the dorsolateral prefrontal cortex downloaded from the AMP-AD Knowledge Portal showed that fibroblasts (Fib) had the highest *CYP1B1* gene expression across all cell types^27^. Among the eight major brain cell types, excitatory neurons (Exc) had the highest *CYP1B1* expression (Fig. 5D). Finally, eQTL analysis of cell type specific *CYP1B1* expression in excitatory neurons showed that the rs2113389 T-allele was associated with higher cell type-specific *CYP1B1* expression levels (*p*-value = 0.035; Fig. 5E).

### Blood-based DNA methylation QTLs of rs2113389

The DNA methylation QTL (meQTL) analysis of rs2113389 with CpGs in *CYP1B1* in blood identified three CpGs located in the *CYP1B1* gene body^28^ associated with rs2113389 (*p*-value < 1 × 10^−5^; Fig. 5F). The rs2113389 T-allele was associated with higher CpG expression levels.

### Cyp1b1 expression and expression changes in the brain of AD mice

*Cyp1b1* expression was increased in the cortex of 6-month-old hTAU mice (*p*-value = 0.038; Fig. 5G). *Cyp1b1* expression also significantly changed with time (genotype*age) in rTg4510 mice (FDR corrected *p*-value = 0.040) but not J20 mice relative to wild-type mice (Fig. 5H, I)^21^. *Cyp1b1* differential expression over time in the TG rTg4510 mice was associated with entorhinal cortex tau pathology (FDR-corrected *p*-value = 0.002; Supplementary Table S5 in Castanho et al.)^21^.

## Discussion

We performed a GWAS of cortical tau PET and identified and replicated a novel SNP at the *CYP1B1*-*RMDN2* locus at 2p22.2. The most significant SNP at the locus was rs2113389, with the minor allele (T) of rs2113389 associated with higher tau across diagnoses. An additive effect of the T-allele with *APOE4* status and Aβ positivity was also observed, with *APOE4*+ and Aβ+ minor T-allele carriers having the highest tau levels. In sex-stratified analyses, generally similar results were observed. Overall, these results provide converging evidence that the minor allele (T) of rs2113389 is a risk variant for high tau. Voxel-wise whole brain analysis confirmed that the rs2113389 T-allele was associated with tau in AD-related cortical regions. These findings also support a previous GWAS of CSF tau, where rs1478361, which is in strong LD with rs2113389 (*r*^2^ = 0.96 and D’ = 1.00), was associated with CSF total tau levels (*n* = 3,076; β = 0.0176; *p*-value = 0.0295)^14^. However, recent large-scale AD GWAS studies have shown that the two SNPs were not significantly associated with AD with different association directions across the studies (*p*-value > 0.05)^4,29–31^. This lack of significant association may reflect heterogeneity in case-control ascertainment based on clinical diagnosis and is consistent with a selective association elucidated using quantitative endophenotype analysis.

The two protein coding genes at the locus identified in this analysis (*CYP1B1* and *RMDN2*) are highly expressed in the brain in the frontal and temporal lobes (*CYP1B1*) and the cortex (*RMDN2)*. Regions showing higher expression levels overlap with the typical patterns of tau deposition, suggesting a spatial relationship between gene expression levels and tau deposition. *RMDN2* (Regulator of Microtubule Dynamics 2) is down-regulated in the parahippocampal gyrus in AD, while *CYP1B1* (Cytochrome P450 Family 1 Subfamily B Member 1) is up-regulated in the temporal cortex in AD. The rs2113389 minor allele is associated with higher temporal cortex *CYP1B1* expression levels. Fibroblasts and excitatory neurons had the highest expression levels of the *CYP1B1*, and in excitatory neurons, the rs2113389 minor allele was associated with higher *CYP1B1* expression levels. Blood-based meQTL analysis also supported the impact of rs2113389 on CpGs within the *CYP1B1* gene, with the rs2113389 T-allele associated with higher CpG expression. Finally, *Cyp1b1* expression was higher in the cortex of 6-month-old hTAU mice relative to controls. In longitudinal analysis, *Cyp1b1* expression changed with aging in rTg4510 mice but not J20 mice, suggesting *Cyp1b1* expression is associated with tau but not amyloid pathology.

*CYP1B1* is of particular interest as the eQTL analysis shows altered temporal lobe expression in AD patients, and the rs2113389 genotype is linked to temporal lobe *CYP1B1* expression. *CYP1B1* is a member of the cytochrome p450 enzyme family (CYP). CYP is present and active in the brain and expressed in a region- and cell-specific manner, including in the blood-brain barrier^32–34^. CYP is responsible for oxidative metabolism of exogenous and endogenous substrates, potentially having both neuroprotective and pathologic roles^33^. CYP is also involved in modulating blood flow, metabolism of fatty acids, cholesterol, and neurotransmitters, and mobilization of intracellular calcium^35–38^, suggesting multiple potential roles in AD. Previously, genetic variants in CYP genes have been associated with neurodegenerative diseases, including AD^39,40^, as well as Aβ and tau^35,41–43^. *CYP1B1* regulates endogenous pathways involved in the metabolism of drugs and the synthesis of cholesterols, steroids, and other lipids^44^. While several cytochrome P450 family genes have been implicated in AD, *CYP1B1* has not previously been directly implicated in AD^39,40,43^. However, *CYP1B1* may have multiple potential roles related to AD-related tau pathology and has been shown to be a regulator of oxidative stress, which promotes angiogenesis^45,46^. *CYP1B1* also promotes angiogenesis by suppressing NF-kB activity, which is also implicated in inflammation^47^. Previous studies suggest that *CYP1B1* inhibition reduced oxidative stress and metabolized cell products that modulate intracellular oxidative stress; however, a lack of *CYP1B1* leads to increased intracellular oxidative stress in the endothelium^48–50^. *CYP1B1* may play an important role in high fat diet-associated learning and memory deficits and oxidative damage^50^. Increased brain oxidative stress causes cell damage with aging and is an important pathogenic factor in AD, contributing to tau phosphorylation and the formation of neurofibrillary tangles^51–53^. Functional studies for *RMDN2* are limited, only showing that it encodes a protein important for regulating microtubule dynamics.

Pathway-based analysis identified enrichment in pathways related to the MHC, postsynaptic membrane, postsynaptic density, synapse organization, and calcium channel activity. MHC pathways have been implicated in large-scale AD genetic associations^4,29,54^, along with specific MHC alleles^55^. Microglial activation via MHC class II signaling is increased in regions of phosphorylated tau^56^. Dysfunctional synaptic connections are involved early in AD-related cognitive impairment^57^, and tau deposition may induce synaptic impairment and learning deficits^58,59^. Studies also suggest a role for tau at dendritic spines in affecting the trafficking of postsynaptic receptors^60,61^. Finally, the Ca^2+^ signaling and homeostasis are implicated in AD pathology^62^ and have been linked to tau phosphorylation^63,64^. Treatments targeting calcium channels are potential pathways for novel therapeutics for neurodegenerative diseases^64^.

There are some notable limitations, as this study was primarily observational and composed only of European ancestry cohorts. Multiethnic studies are important, and to be generalizable to other populations, our findings require replication using large community studies or international collaborations. Although similar methodologies were used in all cohorts, subtle differences due to Freesurfer version or slightly different reference regions for SUVR calculation are possible. Further, all cohorts except AIBL-2 employed the same tau PET tracer ([^18^F]flortaucipir), which may have introduced additional variability. However, the replication of the genetic association in an independent cohort using a different tau PET tracer lends confidence to the generalizability of the findings. Minor sex differences were observed in the pattern of results. Although sex differences are increasingly recognized as important for precision medicine in ADRD, the current study was not designed or powered to thoroughly test these effects. Future studies that assess the presence and pattern of sex differences in longitudinal studies with larger samples are warranted. Even though a number of the cohorts included in the present manuscript have longitudinal follow-up, the current study focused primarily on cross-sectional associations. Future studies to evaluate longitudinal follow-up in these cohorts, including analyses of longitudinal tau PET phenotypes, are also warranted. The Allen Human Brain Atlas results suggest that the genes identified in this analysis are expressed in tau-relevant brain regions. However, these findings do not indicate that expression of these genes is exclusive to brain regions with high tau. Notably, the AHBA did not include patients with ADRD, which limits their utility for disease-related hypotheses. Finally, although we performed the largest GWAS of tau PET to date, our meta-analysis had limited statistical power due to the moderate sample size for genetic association. Additional independent large cohorts with tau PET and GWAS data are needed.

In summary, GWAS of tau PET identified novel genetic variants in a locus (*CYP1B1*-*RMDN2*) that influences MTL and cortical tau levels. The mechanistic significance of this locus was supported by a range of independent functional genomic observations in humans and model systems. Taken together, these results can inform future biomarker and therapeutic development.

## Methods

### Participants

The study complies with all relevant ethical regulations. Informed consent was obtained for all participants according to the Declaration of Helsinki, and studies were approved by the Human Subjects & Institutional Review Boards (IRB) at Indiana University (Alzheimer’s Disease Genomics: Systems Biology and Endophenotypes, 1806870105) as well as the Institutional Review Boards at each participating site. All animal studies were performed in accordance with US National Institutes of Health guidelines on animal care and were approved by appropriate Institutional Animal Care and Use Committees^21^. Descriptions of all cohorts are found in the Supplementary information (Supplementary Tables 1–12). Participants were from the Alzheimer’s Disease Neuroimaging Initiative (ADNI; http://adni.loni.usc.edu), ADNI-Department of Defense (ADNI-DoD), Indiana Memory and Aging Study (IMAS), Avid A05 clinical trial (A05), Anti-Amyloid Treatment in Asymptomatic Alzheimer’s (A4) and Longitudinal Evaluation of Amyloid Risk and Neurodegeneration (LEARN) studies, Harvard Aging Brain Study (HABS), University of Pittsburgh Alzheimer’s Disease Research Center (UPitt ADRC), Mayo Clinic Study of Aging (MCSA), Memory and Aging Project (MAP) at the Knight Alzheimer’s Disease Research Center (Knight-ADRC), the Australian Imaging, Biomarker and Lifestyle Study (AIBL; (https://aibl.org.au/), and the Berkeley Aging Cohort Study (BACS). The discovery sample included ADNI, ADNI-DoD, IMAS, A05, A4, HABS, UPitt ADRC. The replication sample included MCSA, MAP-Knight ADRC, AIBL, and BACS. Post-hoc analyzes of interactions with diagnosis, APOE4, and Aβ positivity, and voxel-wise analyzes were performed in 1161 individuals from ADNI, ADNI-DoD, IMAS, A05, A4, and LEARN.

### Genotyping and imputation

Participants were genotyped using several genotyping platforms. Un-genotyped SNPs were imputed separately in each cohort using the Haplotype Reference Consortium (HRC) data as a reference panel^65^. Before imputation, standard sample and SNP quality control (QC) procedures were performed^66^. Only non-Hispanic participants of European ancestry by multidimensional scaling analysis were selected^67^. Imputation and QC procedures were performed as described previously^68^.

### Statistical analysis

#### Genome-wide association analysis (GWAS)

Cortical tau deposition (the weighted average SUVR of all cortical regions from FreeSurfer version 6.1 parcellation (aparc)) followed a normal distribution after a rank-based inverse normal transformation. Using imputed genotypes, a GWAS of cortical tau was performed using a linear regression model with age, sex, two principal component (PC) factors from population stratification, APOE4 status, and diagnosis as covariates using PLINK^69^. APOE4 status was included as a covariate because its effect was modeled to understand the contribution of the discovered *CYP1B1*-*RMDN2* locus above and beyond *APOE4* and to assess whether there is epistasis with *APOE4*. A fixed effect meta-analysis with an inverse variance weighted approach was performed using METAL, and a heterogeneity analysis in METAL was performed to evaluate the possible effect of study heterogeneity on the results^31,54,70^. See Supplementary information for more details. The proportion of variance in tau explained was assessed using the Genome-wide Complex Trait Analysis (GCTA) tool^71^.

#### Gene-set enrichment analysis

Gene-set enrichment analysis was performed using GWAS summary statistics to identify pathways and functional gene sets associated with cortical tau deposition using the GSA-SNP software^72^, as described in the Supplementary information.

#### Gene-based association analysis

Genome-wide gene-based association analysis was performed using GWAS p-values and the KGG software as described previously^73,74^ and in the Supplementary information.

#### Interaction with diagnosis, APOE genotype, and Aβ positivity

The effect of the top identified SNP (rs2113389 – dominant model) and its interaction with diagnosis, APOE4 status, and Aβ positivity, on global and medial temporal lobe (MTL) tau was assessed. Differential effects by sex were also evaluated using stratified analysis. See methods in Supplementary information.

#### Detailed whole-brain imaging analysis

Tau PET SUVR images (*n* = 1161) were used in a voxel-wise statistical analysis of the effect of the top identified SNP on tau using SPM12 (www.fil.ion.ucl.ac.uk/spm/) in a post-hoc analysis (described in the Supplementary information).

#### CSF tau analysis

CSF total tau and phosphorylated tau 181 values from the Roche Elecsys assay^75,76^ were available for a subset (*n* = 525; 332 CN, 153 MCI, 40 AD) of the ADNI and ADNI-DoD cohorts. Total tau and pTau181 levels were not normally distributed, and thus, we transformed using a natural log before analysis. A one-way ANOVA with rs2113389 genotype as the independent variable using both an additive model and dominant model was used to test the association of rs2113389 genotype and CSF total tau and pTau181 levels, covaried for age, sex, *APOE* ε4 carrier status, and diagnosis.

#### AMP-AD bulk RNA-Seq data in the post‑mortem human brain

Processed RNA-Seq data from seven brain regions in three cohorts were downloaded from the AMP-AD Knowledge Portal (10.7303/syn2580853) and analyzed as discussed in the Supplementary information^26^. The eQTLGen^77^ consortium database (*n* = 31,684) was used for eQTL of rs2113389 with CYP1B1 expression in blood.

#### Single-nucleus RNA-Seq (snRNA-Seq) preprocessing and analysis

Processed snRNA-Seq data from frozen brain tissue specimens (*n* = 479) from the dorsolateral prefrontal cortex in the Religious Orders Study/Memory and Aging Project (ROSMAP) was downloaded from the AMP-AD Knowledge Portal (https://www.synapse.org/#!Synapse:syn31512863)^27,78^.

#### Allen Human Brain Atlas data and analysis

Regional gene expression profiles for CYP1B1 and RMDN2 were downloaded from brain-wide microarray-based transcriptome data from the Allen Human Brain Atlas (https://human.brain-map.org/microarray/search), as described in the Supplementary information^79,80^.

#### ADNI DNA methylation data

ADNI DNA methylation data was downloaded from the ADNI LONI database (https://adni.loni.usc.edu/), where Illumina EPIC chips (Illumina, Inc., San Diego, CA, USA) were used to profile DNA methylation in 1920 blood or buffy coats samples including 200 duplicate samples according to the Illumina protocols^28^. A detailed protocol has been published previously^28,81,82^, and further methods are described in the Supplementary information.

#### AD pathology mouse model analysis

hTau mouse model: Generation of the hTAU mice, as well as brain extraction and tissue processing, was described previously^19,20,83,84^ and in the Supplementary information. Student’s t-test was performed for qPCR results comparing C57BL/6 J (B6; wild type) and hTAU mice. rTg4510 and J20 mouse model: Mice harboring human tau (rTg4510) and amyloid precursor protein (J20) mutations were used to investigate gene expression changes of the top identified gene^21^. The rTg4510 and J20 mouse models and experimental models and methods were described previously^21,85–88^, and are briefly summarized, along with statistical methods used, in the Supplementary information.

### Reporting summary

Further information on research design is available in the Nature Portfolio Reporting Summary linked to this article.

## Supplementary information

Supplementary Information

Peer Review File

Reporting Summary

## Source data

Source Data

## Data Availability

Summary statistics for the discovery analysis are available in the Alzheimer’s Disease Neuroimaging Initiative Laboratory of NeuroImaging repository (ADNI LONI; https://ida.loni.usc.edu/pages/access/studyData.jsp?categoryId=18&subCategoryId=28). Referenced data, including imaging, cognitive, clinical and genetic data from ADNI, A4, and ADNI-DoD can be requested through the Laboratory of NeuroImaging (LONI; https://www.loni.usc.edu/). Imaging data for AIBL is available through the Laboratory of NeuroImaging (LONI; https://www.loni.usc.edu/), while genetic and other data is available by request from the study PIs. Referenced imaging, cognitive, clinical, and genetic data from the other human cohorts (IMAS, A05, HABS, UPitt ADRC, BCSA, MCSA, and the Knight ADRC) is not publicly available and must be requested directly from the study PIs. ADNI DNA methylation data was downloaded from the ADNI LONI database (https://adni.loni.usc.edu/)^28^. Brain-wide microarray-based transcriptome data from the Allen Human Brain Atlas is available through the Allen Brain Map portal (https://human.brain-map.org/microarray/search). RNA-Seq data is available through the AMP-AD Knowledge Portal (10.7303/syn2580853)^26^. ROSMAP single-nucleus RNA-Seq data is available through the AD Knowledge Portal (https://www.synapse.org/#!Synapse:syn31512863)^27^. Data is available for general research use according to the following requirements for data access and data attribution (https://adknowledgeportal.synapse.org/DataAccess/Instructions). The data from the rTg4510 and J20 mouse models is available in a previous paper^21^. The data from the hTau mouse model is provided. No primary data was generated in this study, as all data used were reference datasets. Source data for the figures included in this paper are provided. Source data are provided with this paper.
