# Supplementary material for: CYP1B1-RMDN2 Alzheimer’s disease endophenotype locus identified for cerebral tau PET
Source: Nat Commun. 2024 Sep 20;15:8251. doi: 10.1038/s41467-024-52298-2 (PMC11415491; doi:10.1038/s41467-024-52298-2)
Supplement: Supplementary file 1 — Supplementary Information [file 41467_2024_52298_MOESM1_ESM.pdf]

Supplementary Material

CYP1B1-RMDN2 Alzheimer's disease endophenotype locus identified for cerebral tau PET

Kwangsik Nho, Shannon L. Risacher, et al.

Correspondence to: asaykin@iu.edu

TABLE OF CONTENTS

Supplementary Methods and Tables

Supplementary Methods (Includes Supplementary Tables 1 – 12) – Pages 2-18

Supplementary Table 13 – Pages 27-28

Supplementary Figures

Supplementary Figure 1 – Page 29-30

Supplementary Figure 2 – Page 31-32

Supplementary Figure 3 – Page 33-34

Supplementary Figure 4 – Page 35-36

Supplementary Figure 5 – Pages 37-38

Supplementary Figure 6 – Pages 39-40

Supplementary Figure 7 – Pages 41-42

Supplementary Figure 8 – Pages 43-44

Supplementary Figure 9 – Pages 45-46

Supplementary Figure 10 – Page 47-48

Supplementary Figure 11 – Page 49

Supplementary References – Pages 50-55

Consortia

Alzheimer's Disease Neuroimaging Initiative (ADNI) – Pages 56-57

Department of Defense Alzheimer's Disease Neuroimaging Initiative (DoD-ADNI) – Page 57

Anti-Amyloid Treatment in Asymptomatic Alzheimer's Study (A4 Study) and Longitudinal Evaluation of Amyloid Risk and Neurodegeneration (LEARN) – Pages 57-59

Australian Imaging Biomarkers and Lifestyle (AIBL) – Page 59

Supplementary Methods

Discovery sample cohorts

<u>Alzheimer's Disease Neuroimaging Initiative (ADNI)</u>: The ADNI initial phase (ADNI-1) was launched in 2003 to test whether serial magnetic resonance imaging (MRI), position emission tomography (PET), other biological markers, and clinical and neuropsychological assessment could be combined to measure the progression of mild cognitive impairment (MCI) and early AD. ADNI-1 has been extended in subsequent phases (ADNI-GO, ADNI-2, and ADNI-3) for follow-up of existing participants and additional new enrollments. More information about ADNI can be found in previous publications[1-4] and at https://adni.loni.usc.edu. Demographic information, pre-processed tau PET scans, *APOE* and whole-genome genotyping data, neuropsychological test scores, and clinical information are publicly available from the LONI ADNI data repository. The study complies with all relevant ethical regulations. Informed consent was obtained for all participants according to the Declaration of Helsinki, and studies were approved by the relevant Institutional Review Boards. A total of 567 ADNI participants (351 cognitively normal older adults (CN), 168 MCI, and 48 AD) were available for analysis. [^{18}F]flortaucipir scans were collected using previously described methods (http://adni.loni.usc.edu [5]). Pre-processed [^{18}F]flortaucipir, [^{18}F]florbetapir, and [^{18}F]florbetaben scans were also downloaded from the ADNI data repository and processed using standard techniques using Statistical Parametric Mapping (SPM12), including normalization to MNI (Montreal Neurological Institute) space. Then, for [^{18}F]flortaucipir, standardized uptake value ratio (SUVR) images were created by intensity-normalization using a cerebellar crus reference region. Mean [^{18}F]flortaucipir SUVR was extracted from a global cortical grey matter region generated from subject-specific parcellations of structural MPRAGE images generated using

FreeSurfer version 6, which included all regions from the Freesurfer parcellation (aparc) based on the Desikan-Killiany atlas.[6] For [^{18}F]florbetaben and [^{18}F]florbetapir, scans were processed using the Centiloid (CL) method as previously described,[7-9] using local formulas derived from the stage 2 process as described in Risacher et al.[9] Global cortical CL≥20.76 was considered as Aβ positive (Aβ+), as this cut-off best predicted the SUVR cut-offs produced by the University of California - Berkeley (SUVR>1.11 for [^{18}F]florbetapir and SUVR>1.08 for [^{18}F]florbetaben). Demographic and neuropsychological performance data for ADNI can be found in **Supplementary Table 1**. P-values represent the results of AN(C)OVA or chi-squared statistical tests that were used to compare differences between diagnostic groups in continuous and categorical data, respectively. Covariates included for each variable (if any) are indicated in the table footer.

Supplementary Table 1. ADNI Demographics (Mean (Standard Deviation))

	**CN (n=351)**	**MCI (n=168)**	**AD (n=48)**	**p-value**
Age (years)	73.2 (7.0)	74.9 (8.1)	77.4 (9.8)	$4.76x10^{-4}$
Sex (M, F)	165, 186	97, 71	28, 20	0.042
Education (years)	16.7 (2.4)	16.3 (2.7)	16.2 (2.7)	0.088
APOE ε4 carrier (%)	36.5%	36.3%	56.3%	0.026
Aβ Positivity (%)[a]	54.3%	56.9%	66.7%	0.261
FAQ Total Score[b]	1.4 (4.5)	3.3 (5.6)	8.9 (9.6)	$3.44x10^{-17}$
CDR Global Score[c]	0.1 (0.3)	0.4 (0.3)	0.6 (0.6)	$7.28x10^{-27}$
CDR Sum of Boxes[c]	0.5 (1.6)	1.4 (1.9)	3.5 (3.6)	$1.71x10^{-20}$
MMSE Total Score[d,h]	28.5 (2.4)	27.6 (3.1)	25.3 (4.3)	$2.69x10^{-10}$
ADAS Total Score[e,h]	6.0 (4.2)	9.1 (5.4)	14.8 (9.1)	$1.91x10^{-19}$
Story Recall Z-Score – Learning[f,i]	0.0 (1.1)	-0.6 (1.2)	-1.1 (1.5)	$6.02x10^{-12}$
Story Recall Z-Score – Delayed[f,i]	0.0 (1.2)	-0.7 (1.2)	-1.3 (1.6)	$7.77x10^{-15}$
Trail Making – Part A[g,i]	32.4 (12.4)	36.8 (18.9)	43.8 (26.2)	$3.10x10^{-4}$
Trail Making – Part B[g,i]	84.5 (55.7)	98.9 (62.4)	133.1 (61.8)	$1.58x10^{-4}$
Animal Fluency[h,i]	20.8 (6.2)	18.7 (5.8)	17.1 (6.6)	0.002

[a] 2 participants (1 CN, 1 MCI) missing Aβ positivity<br>[b] 21 participants (11 CN, 8 MCI, 2 AD) missing FAQ score<br>[c] 19 participants (10 CN, 6 MCI, 3 AD) missing CDR scores<br>[d] 13 participants (8 CN, 3 MCI, 2 AD) missing MMSE score<br>[e] 85 participants (47 CN, 29 MCI, 9 AD) missing ADAS score	[f] 13 participants (6 CN, 5 MCI, 2 AD) missing Story Recall z-scores (*z-score calculated relative to CN individuals)<br>[g] 20 participants (8 CN, 9 MCI, 3 AD) missing Trail Making Test scores<br>[h] 12 participants (5 CN, 5 MCI, 2 AD) missing Animal Fluency<br>[i] Covaried for age, sex, and years of education<br>n.s. = not significant

<u>ADNI-Department of Defense (ADNI-DoD):</u> ADNI-DoD is an extension of the original ADNI (described above) to investigate the relationship of traumatic brain injury (TBI) and post-traumatic stress disorder (PTSD) on the development of AD. ADNI-DoD aims to enroll approximately 400 elderly Veterans who served in Vietnam, including some individuals with PTSD and/or TBI, as well as controls. Individuals in the study are diagnosed as CN or MCI using ADNI criteria (http://www.loni.usc.edu/ADNI). Participants in ADNI-DoD undergo all procedures that are collected as part of the original ADNI, including cognitive tests and clinical examinations, amyloid PET with [^{18}F]florbetapir, structural and functional MRI, lumbar puncture for CSF analysis, and blood collection for genetic analysis, including *APOE* and genome-wide data. In addition, a subset of participants also underwent tau PET imaging with [^{18}F]flortaucipir. A total of 68 participants from ADNI-DoD (55 CN, 13 MCI) were included in the present analysis. The study complies with all relevant ethical regulations. Informed consent was obtained for all participants according to the Declaration of Helsinki, and studies were approved by the relevant Institutional Review Boards. For more information, see a previous report from Weiner et al.[10] [^{18}F]flortaucipir scans from ADNI-DoD were processed using an identical processing pipeline to that in the ADNI study. Specifically, [^{18}F]flortaucipir scans were collected using previously described methods (http://adni.loni.usc.edu)[5]. Pre-processed [^{18}F]flortaucipir scans were downloaded from the ADNI data repository (http://adni.loni.usc.edu) and processed using standard techniques using Statistical Parametric Mapping (SPM12), including normalization to MNI (Montreal Neurological Institute) space. Then, standard uptake value ratio (SUVR) images were created by intensity-normalization using a cerebellar crus

reference region. Mean [^{18}F]flortaucipir SUVR was extracted from a global cortical grey matter region generated from subject-specific parcellations of structural MPRAGE images generated using from FreeSurfer version 6. As in ADNI, amyloid scans were also collected for 67 participants (1 MCI missing amyloid data) using only [^{18}F]florbetapir. Briefly, static images were downloaded from the ADNI data repository (http://adni.loni.usc.edu) and processed using standard methods, including normalization to MNI space, in SPM12. Again, scans were processed using the CL method as described above and previously,[7-9] and CL ≥ 20.76 was consider Aβ+. Demographic and neuropsychological performance data for ADNI-DoD can be found in **Supplementary Table 2**. P-values represent the results of AN(C)OVA or chi-squared statistical tests that were used to compare differences between diagnostic groups in continuous and categorical data, respectively. Covariates included for each variable (if any) are indicated in the table footer.

**Supplementary Table 2. ADNI-DoD Demographics (Mean (Standard Deviation))**			
	**CN (n=55)**	**MCI (n=13)**	**p-value**
Age (years)	71.1 (5.6)	70.1 (2.5)	0.527
Sex (M, F)	54, 1	13, 0	0.624
Education (years)	15.4 (2.5)	15.0 (2.9)	0.580
APOE ε4 carrier (%)	25.5%	15.4%	0.441
Aβ Positivity (%)[a]	23.6%	25.0%	0.920
FAQ Total Score[b]	1.3 (2.5)	4.8 (5.7)	0.012
CDR Global Score[c]	0.1 (0.2)	0.4 (0.2)	1.80×10^{-9}
CDR Sum of Boxes[c]	0.3 (0.7)	1.3 (1.3)	2.77×10^{-4}
MMSE Total Score[f]	28.4 (1.8)	27.5 (2.6)	0.248
ADAS-11 Total Score[f]	6.4 (3.5)	8.5 (3.6)	*0.090*
Story Recall Z-Score – Learning[d,f]	0.0 (1.1)	-1.1 (1.2)	2.70×10^{-3}
Story Recall Z-Score – Delayed[d,f]	0.1 (1.1)	-1.2 (1.0)	3.97×10^{-4}
Trail Making – Part A[e,f]	36.7 (17.8)	49.4 (33.6)	*0.077*
Trail Making – Part B[e,f]	82.4 (27.4)	150.8 (84.6)	5.91×10^{-6}
Animal Fluency[f]	20.9 (5.0)	17.2 (5.6)	0.023

[a] 1 MCI participant missing Aβ positivity<br>[b] 31 participants (28 CN, 3 MCI) missing FAQ score<br>[c] 9 CN participants missing CDR scores	[d] 1 CN participant missing Trail Making Test scores<br>[e] z-score calculated relative to CN individuals<br>[f] Covaried for age, sex, and years of education<br>n.s. = not significant

Indiana Memory and Aging Study (IMAS): IMAS is a longitudinal observational study of older adults at risk for and with prodromal and clinical AD. As part of the IMAS study, participants underwent a comprehensive clinical visit through the Indiana Alzheimer's Disease Research Center (IADRC), which included clinical and neurologic testing, neuropsychological testing with the Uniform Dataset 3 (UDS-3) battery, and blood samples, as well as structural and functional MRI, [^{18}F]flortaucipir PET scans, and amyloid PET scans with either [^{18}F]florbetapir or [^{18}F]florbetaben. AD and MCI were diagnosed using standard criteria.[11,12] CN were older adults without a significant performance deficit on cognitive testing. A total of 54 participants from IMAS (30 CN, 15 MCI, 9 AD) were included in the present analysis. The study complies with all relevant ethical regulations. Informed consent was obtained for all participants according to the Declaration of Helsinki, and studies were approved by the relevant Institutional Review Boards. In IMAS, [^{18}F]flortaucipir scans were collected as previously described.[8,9] Using SPM12, the middle four 5-minute frames (80-100 minutes) were motion corrected, normalized to MNI space, averaged to create an 80-100 minute static image, intensity normalized to the cerebellar crus to create SUVR images, and smoothed with an 8mm FWHM (Full Width at Half Maximum) Gaussian kernel. Mean [^{18}F]flortaucipir SUVR was extracted from a global cortical grey matter region generated from subject-specific parcellations of structural MPRAGE images generated using from FreeSurfer version 6. Amyloid scans were also collected for 55 individuals in IMAS (3 MCI missing amyloid data) using both [^{18}F]florbetapir and [^{18}F]florbetaben in IMAS as previously described.[8,9] Using SPM12, the four 5-minute frames were spatially aligned to each participant's individual magnetization-prepared rapid gradient-echo (MPRAGE) scan, motion

corrected, normalized to Montreal Neurologic Institute (MNI) space, and averaged to create a 50-70 minute static image for the [^{18}F]florbetapir or a 90-110 minute static image for the [^{18}F]florbetaben. Then SUVR images were created by intensity-normalizing to the whole cerebellum for both tracers. The whole cerebellum ROI was from the CL project. The resulting SUVR images were converted to CL units and smoothed using an 8mm full-width half maximum (FWHM) Gaussian kernel. Again, CL ≥ 20.76 was consider Aβ+. Demographic and neuropsychological performance data for IMAS can be found in **Supplementary Table 3**. P-values represent the results of AN(C)OVA or chi-squared statistical tests that were used to compare differences between diagnostic groups in continuous and categorical data, respectively. Covariates included for each variable (if any) are indicated in the table footer.

Supplementary Table 3. IMAS Demographics (Mean (Standard Deviation))

	**CN (n=30)**	**MCI (n=15)**	**AD (n=9)**	**p-value**
Age (years)	70.1 (7.7)	72.6 (8.3)	64.4 (11.2)	*0.083*
Sex (M, F)	8, 22	8, 7	4, 5	0.192
Education (years)	17.0 (2.2)	15.6 (3.3)	15.6 (2.6)	0.149
APOE ε4 carrier (%)	40.0%	66.7%	55.6%	0.226
Aβ Positivity (%)[a]	23.3%	41.7%	77.8%	0.012
FAQ Total Score	0.2 (0.6)	4.5 (4.4)	10.8 (6.5)	5.38×10^{-10}
CDR Global Score	0.1 (0.2)	0.5 (0.1)	0.7 (0.5)	7.11×10^{-10}
CDR Sum of Boxes	0.1 (0.3)	1.9 (1.2)	4.7 (3.0)	2.28×10^{-11}
MMSE Total Score[b,e,*]	29.6 (0.8)	26.3 (3.2)	21.6 (5.1)	1.73×10^{-8}
Story Recall Z-Score – Learning[c,i]	0.0 (1.0)	-1.4 (0.9)	-2.3 (0.5)	1.05×10^{-8}
Story Recall Z-Score – Delayed[c,i]	0.0 (1.0)	-2.3 (0.9)	-2.9 (0.6)	8.40×10^{-13}
Trail Making – Part A[d,e]	26.2 (9.2)	34.9 (11.3)	44.9 (14.5)	3.31×10^{-4}
Trail Making – Part B[d,e]	65.5 (24.7)	107.1 (35.7)	182.9 (64.2)	4.17×10^{-9}
Animal Fluency[e]	25.0 (4.3)	16.8 (4.6)	11.3 (5.5)	9.36×10^{-10}

[a] 3 MCI participants missing Aβ positivity
[b] 1 MCI participant missing MMSE score
[c] 1 AD participant missing Story Recall z-scores (*z-score calculated relative to CNs)
[d] 2 AD participants missing Trail Making Test scores
[e] Covaried for age, sex, and years of education
* imputed from MoCA scores using [13]
n.s. = not significant

A05 Study: The A05 study complies with all relevant ethical regulations. Informed consent was obtained for all participants according to the Declaration of Helsinki, and studies were approved

by the relevant Institutional Review Boards. 169 participants (51 CN, 74 MCI, 44 AD) were recruited at 25 sites and underwent cognitive testing and clinical assessment, structural MRI, amyloid PET with [^{18}F]florbetapir, tau PET with [^{18}F]flortaucipir, and blood collection for genetic analysis, including *APOE* and genome-wide data.[14,15] Structural MRIs, tau scans, and genome-wide data were provided by Avid/Eli Lilly under a research agreement, while clinical and cognitive data, *APOE* genotype (2 CN, 3 MCI missing *APOE* genotype data), and Aβ positivity (missing for 1 AD patient) were provided in a summary dataset. Tau PET scans were provided as 80-100 minute static images, which were subsequently co-registered to structural MRI scans from the same visit and normalized to MNI space using parameters from segmentation of the structural MRIs using SPM12. Then, standard uptake value ratio (SUVR) images were created by intensity-normalization using a cerebellar crus reference region. Mean [^{18}F]flortaucipir SUVR was extracted from a global cortical grey matter region generated from subject-specific parcellations of structural MPRAGE images generated using from FreeSurfer version 6. Demographic and neuropsychological performance data for A05 can be found in **Supplementary Table 4**. P-values represent the results of AN(C)OVA or chi-squared statistical tests that were used to compare differences between diagnostic groups in continuous and categorical data, respectively. Covariates included for each variable (if any) are indicated in the table footer.

**Supplementary Table 4. A05 Demographics (Mean (Standard Deviation))**				
	**CN (n=51)**	**MCI (n=74)**	**AD (n=44)**	**p-value**
Age (years)	63.6 (18.2)	71.6 (9.1)	74.0 (9.0)	$1.38x10^{-4}$
Sex (M, F)	29, 22	41, 33	20, 24	0.477
APOE ε4 carrier (%)	19.6%	48.6%	54.5%	$6.37x10^{-4}$
Aβ Positivity (%)[a]	9.8%	50.0%	65.1%	$6.35x10^{-8}$
FAQ Total Score[b]	n/a	4.3 (4.9)	15.2 (6.9)	$2.23x10^{-17}$
MMSE Total Score[e]	29.6 (0.5)	27.9 (1.8)	22.0 (4.0)	$3.60x10^{-33}$

ADAS Total Score[e]	5.4 (3.1)	10.1 (4.4)	19.9 (8.7)	$1.19x10^{-23}$
Story Recall Z-Score – Learning[c,e]	0.0 (1.0)	-1.0 (0.8)	-2.0 (-0.8)	$2.57x10^{-21}$
Story Recall Z-Score – Delayed[c,e]	0.0 (1.0)	-1.0 (0.8)	-2.1 (0.5)	$9.14x10^{-26}$
Trail Making – Part A[d,e]	35.2 (15.1)	48.2 (21.4)	73.2 (43.6)	$3.45x10^{-8}$
Trail Making – Part B[d,e]	86.7 (42.8)	126.5 (77.3)	204.4 (101.2)	$1.53x10^{-9}$
Animal Fluency[e]	21.6 (6.3)	16.8 (4.7)	9.6 (4.6)	$1.48x10^{-17}$

[a] 1 AD participants missing Aβ positivity
[b] All CN participants (n=51) missing FAQ score
[c] Z-score calculated relative to CN individuals
[d] 3 AD participants missing Trail Making Test scores
[e] Covaried for age, sex, and years of education
n.s. = not significant

A4/LEARN Study: The A4 study complies with all relevant ethical regulations. Informed consent was obtained for all participants according to the Declaration of Helsinki, and studies were approved by the relevant Institutional Review Boards. The A4 trial is an ongoing 240-week pharmacological trial testing whether an anti-amyloid treatment, solanezumab, can slow cognitive decline and progression to dementia in older adults with preclinical AD (i.e., amyloid-positive cognitively normal older adults).[16,17] Participants were recruited and assessed at 67 clinical trial sites across the world and included in the study if they were positive on an amyloid PET scan with [^{18}F]florbetapir, cognitively normal, and met all other study criteria. A subset of 303 CN participants, including 248 participants from the main A4 study and 55 participants from part of the LEARN sub-study, received a tau PET scan with [^{18}F]flortaucipir that was downloaded as 80-100 minute status on the LONI data repository (https://ida.loni.usc.edu/home/projectPage.jsp?project=A4). Then, the [^{18}F]flortaucipir scans were co-registered to structural MRI scans from the same visit and normalized to MNI space using parameters from segmentation of the structural MRIs using SPM12. Standard uptake value ratio (SUVR) images were created by intensity-normalization using a cerebellar crus reference region, and mean [^{18}F]flortaucipir SUVR was extracted from a global cortical grey matter region generated from subject-specific parcellations of structural MPRAGE images generated using from FreeSurfer version 6. Amyloid scans were also collected for all participants using only

[^{18}F]florbetapir. Briefly, static images were downloaded from the A4 data repository (http://adni.loni.usc.edu) and processed using standard methods, including normalization to MNI space, in SPM12. Again, scans were processed using the CL method as described above and previously[7-9] and CL ≥ 20.76 was consider Aβ+. Demographic and neuropsychological performance data for A4 can be found in **Supplementary Table 5**.

**Supplementary Table 5. A4/LEARN Demographics (Mean (Standard Deviation))**	
	**CN (n=303)**
Age (years)	71.6 (4.7)
Sex (M, F)	185, 118
Education (years)	16.3 (2.7)
APOE ε4 carrier (%)	54.5%
Aβ Positivity (%)	86.8%
CDR Global Score	0.0 (0.0)
CDR Sum of Boxes	0.1 (0.2)
MMSE Total Score	28.7 (1.3)
Story Recall Z-Score – Learning	-0.1 (0.8)
Story Recall Z-Score – Delayed	-0.1 (0.8)

Genotyping and Imputation for ADNI, ADNI-DOD, A05, A4/LEARN, and IMAS:

Participants were genotyped using several Illumina genotyping platforms. As the cohorts used different genotyping platforms, un-genotyped SNPs were imputed separately in each cohort using MACH and the Haplotype Reference Consortium (HRC) data as a reference panel.[18] Before the imputation, standard sample and SNP quality control procedures were performed as described previously:[19] (1) for SNP, SNP call rate < 95%, Hardy-Weinberg p-value$<1\times10^{-6}$, and minor allele frequency (MAF) < 1%; (2) for sample, sex inconsistencies, and sample call rate < 95%. Furthermore, in order to prevent spurious association due to population stratification, only non-Hispanic participants of European ancestry that clustered with HapMap CEU (Utah residents with Northern and Western European ancestry from the CEPH collection) or TSI

(Toscani in Italia) populations were selected using multidimensional scaling (MDS) analysis (www.hapmap.org).[20] Imputation and quality control procedures were performed as described previously.[21] After the imputation, an r^2 value equal to 0.30 was used as the threshold to accept the imputed genotypes.

Harvard Aging Brain Study (HABS): The HABS study complies with all relevant ethical regulations. Informed consent was obtained for all participants according to the Declaration of Helsinki, and studies were approved by the relevant Institutional Review Boards. HABS is a longitudinal study of aging and dementia that includes more than 300 older adults who were cognitively normal at enrollment. More information about HABS can be found in previous publications.[22-25] A subset of 147 individuals (139 CN, 6 MCI, 2 dementia) from HABS had GWAS, cognitive, *APOE* genotype, and [^{18}F]flortaucipir PET data and were included in this analysis. HABS [^{18}F]flortaucipir PET scan acquisition parameters and processing protocols have been published previously.[23,25,26] SUVR images were generated from 80-100 minutes static images. Global cortical grey matter [^{18}F]flortaucipir SUVR was extracted and used as the phenotype for GWAS analysis. Demographic and neuropsychological performance data for HABS can be found in **Supplementary Table 6**.

In HABS, genome-wide genotyping was performed in two batches, using the same genotyping platform (Axiom™ Biobank Genotyping Array): the first batch (n=189) was genotyped in 2013, while the second batch (n=96) was completed in 2016. We selected single nucleotide polymorphisms (SNPs) and individuals according to the following quality control criteria, using PLINK v1.9: autosomal SNPs with minor allele frequency (MAF)>0.01, genotype missing rate (variant)<0.02, genotype missing rate (sample)<0.02, non-extreme heterozygosity (within 5 s.d.), identity by descent (IBD) pi-hat<0.125, and Hardy-Weinberg equilibrium (HWE) $p>10^{-6}$. We

selected participants of European descent using multi-dimensional scaling (MDS), resulting in 155 participants with 290,073 variants from the 2013 batch, and 61 participants with 264,623 variants from the 2016 batch. These two data files were merged, only taking matching SNPs across two batches, resulting in 216 participants with 239,503 variants. PLINK v1.9 was used to derive genotype principal components that capture population structures. Then, Eagle v2.4 was used to phase the genotypes, and imputation was done through Minimac4 on Michigan Imputation Server using the Haplotype Reference Consortium (HRC) reference panel (r1.1 2016, GRCh37/hg19). After post-imputation QC (MAF>0.01, HWE $p>10^{-6}$, and imputation $R^2>0.9$), we had 5,673,786 autosomal variants from 216 individuals of European ancestry (of which 150 were used in this analysis). Finally, a regression model implemented in PLINK was calculated, and summary association values for all variants were shared with IUSM.

Supplementary Table 6. HABS Demographics (Mean (Standard Deviation))

	**HABS CN (n=139)**	**HABS MCI (n=6)**
Mean age, years (sd)	79.3 (6.2)	80.3 (6.8)
Female (%)	79 (57)	3 (50)
Mean Education, years (sd)	16.6 (3.0)	16 (1.3)
APOE ε4 carrier (%)**	35 (25)	5 (83)
MMSE, median (IQR)	29 (1)	27 (2.3)

*Two dementia cases and three cases with missing diagnoses whose demographics are not summarized to protect participant privacy
**One CN participant with missing *APOE* genotype info

University of Pittsburgh ADRC: Participants were recruited through neuroimaging studies affiliated with the University of Pittsburgh ADRC from studies on cardiovascular risk factors and population-based cohorts from small towns near Pittsburgh and surrounding areas.[27] The study complies with all relevant ethical regulations. Informed consent was obtained for all participants according to the Declaration of Helsinki, and studies were approved by the relevant Institutional Review Boards. 138 individuals (59 CN, 35 MCI/AD, 44 Other) with GWAS data

and [^{18}F]flortaucipir scans were included in the present study. Briefly, [^{18}F]flortaucipir scans were processed using standard techniques, and global cortical tau SUVR was extracted.[27] Genotype data generated from the Illumina Infinium Global Diversity Array (GDA) underwent standard QC and imputed using HRC. Finally, a regression model implemented in PLINK was calculated, and summary association values were shared with IUSM. Demographic and neuropsychological performance data for the University of Pittsburgh ADRC can be found in **Supplementary Table 7**. Covariates included for each variable (if any) are indicated in the table footer.

**Supplementary Table 7. UPitt ADRC Demographics (Mean (Standard Deviation))**	**CN (n=59)**	**MCI/AD (n=35)**	**Other (n=44)**
Age (years)	72.5 (4.5)	74.5 (3.8)	74.4 (5.5)
Sex (M, F)	18, 41	12, 23	14, 30
Education (years)	16.3 (2.6)	14.9 (2.8)	15.3 (2.8)
APOE ε4 carrier (%)	33.9%	22.9%	18.2%
MMSE Total Score[a]	28.5 (1.4)	27.3 (2.2)	27.4 (2.2)
CDR Global Score	0.01 (0.07)	0.23 (0.25)	0.10 (0.23)

[a]6 participants (2 CN, 4 Other) missing MMSE score
n.s. = not significant

Replication Cohorts

<u>Mayo Clinic Study of Aging (MCSA):</u> The MCSA is a community-based study of older adults (aged 70-89) from Rochester, Minnesota and surrounding regions.[28,29] The study complies with all relevant ethical regulations. Informed consent was obtained for all participants according to the Declaration of Helsinki, and studies were approved by the relevant Institutional Review Boards. 649 individuals (571 CN, 66 MCI, 17 AD) with genetic data and [^{18}F]flortaucipir scans were included in this analysis. Briefly, [18F]flortaucipir scans were collected according to

previously described protocols and processed using standard techniques.[30-32] Regional uptake was measured with an in-house automated pipeline with ROI labels propagated from a custom MRI template.[33,34] Global tau burden was calculated from a composite meta-ROI as previously described.[30-32] GWAS data was generated for all participants using the Illumina Infinium Global Screening Array-24 v2.0 and underwent standard QC using PLINK version 1.9 as previously described.[30,35] GWAS data then underwent imputation using the HRC reference panel, and after QC, 6,417,232 SNPs were available for analysis.[30] The replication analysis was calculated using a linear regression model, covaried for age, sex, ancestry principal components (PCs), APOE genotype, and diagnosis for the top SNPs identified from the discovery cohorts. Demographic and neuropsychological performance data for MCSA participants can be found in **Supplementary Table 8**.

Supplementary Table 8. MCSA Demographics (Mean (Standard Deviation))

	**CN (n=571)**	**MCI (n=66)**	**AD (n=17)**
Age (years)	71.1 (10.5)	77.1 (9.3)	83.0 (4.8)
Sex (M, F)	313, 258	38, 28	10, 7
Education (years)	15.0 (2.3)	13.4 (3.1)	13.5 (3.0)
APOE ε4 carrier (%)	28.1%	34.8%	58.8%

Memory and Aging Project (MAP) at the Knight ADRC: For this study, data was collected from different AD-related cohorts enrolled in the MAP at the Knight ADRC. The total sample size included was 309 (279 CN, 30 MCI/AD). Collection of genotype data and PET image processing are described in detail in the respective studies.[36-39] The study complies with all relevant ethical regulations. Informed consent was obtained for all participants according to the Declaration of Helsinki, and studies were approved by the relevant Institutional Review Boards. Briefly, participants were diagnosed as cognitively normal (controls) or AD (cases), based on CDR global score. For each cohort, tau PET SUVR was extracted from a composite of cortical

brain areas.[36,40] The normalized z-score SUVRs were calculated by using the mean and standard deviation (SD) units for all the samples and applied to the entire cohort. Stringent QC steps of the GWAS data were applied. Briefly, we used the threshold of 98% for removing single nucleotide polymorphisms (SNPs) and participants with low call rate. Autosomal SNPs that were not in the Hardy-Weinberg equilibrium ($P<1\times10{-}6$) were also removed. Duplication and relatedness of participants were estimated from identity-by-descent (IBD) analysis carried out in Plink version 1.9.[41] In case of related participants (Pihat ≥ 0.25), the samples with a higher number of variants that passed the QC were prioritized. For phasing and imputation, we used the 1000 Genomes Project Phase 3 data (October 2014), SHAPEIT v2.r837[42] and IMPUTE2 v2.3.2.[43,44] We used imputed probability score<0.90 and ≥ 0.90 as thresholds for missing and fully observed participant genotypes, respectively. Genotyped and imputed variants with MAF<0.02 or IMPUTE2 information score<0.30 were discarded. Principle component analysis (PCA) was performed on the genotype data to obtain genetic PCs that capture population substructure. Statistical analyses and data visualization were performed in Plink version 1.9[45] and R version 3.5.2. We performed association analyses of the strongest variants found in the discovery cohort with tau pathology. The associations were tested using logistic regression model from the base R “stats” package, adjusted for sex, age, *APOE* genotype, diagnosis, and the first two genetic PCs. Demographic and neuropsychological performance data for MAP participants can be found in **Supplementary Table 9**.

**Supplementary Table 9. MAP at the Knight ADRC Demographics (Mean (Standard Deviation))**		
	**CN (n=279)**	**MCI/AD (n=30)**
Age (years)	69.8 (8.1)	77.4 (7.0)
Sex (M, F)	128, 184	16, 16
Education (years)	16.3 (2.3)	15.4 (3.0)

APOE ε4 carrier (%)	33.3%	62.5%

Australian Imaging, Biomarker and Lifestyle Study (AIBL): The AIBL study is a longitudinal cohort study collected at multiple sites in Australia.[46,47] The study complies with all relevant ethical regulations. Informed consent was obtained for all participants according to the Declaration of Helsinki, and studies were approved by the relevant Institutional Review Boards. A subset of AIBL individuals had genetic data and tau PET imaging and thus, were included in this analysis, including 77 individuals (65 CN, 8 MCI, 4 AD) who underwent tau PET with [^{18}F]flortaucipir and 458 who underwent tau PET with [^{18}F]MK-6240 (271 CN, 102 MCI, 85 AD). Briefly, [18F]flortaucipir and [18F]MK-6240 scans were collected as a 20-minute acquisition either 80 minutes or 90 minutes post-injection of tracer, respectively. Scan processing was done using standard methods to generate SUVR images.[48] Finally, mean global cortical [18F]flortaucipir and [18F]MK-6240 SUVR was extracted. GWAS SNP array data with 976,713 SNPs (including 273,000 exome variants and an additional 13,000 custom content SNPs) on the OmniExpressHumanExome+BeadChip (Illumina, USA) was generated. Genetic data was mapped to the human genome reference hg19, and standard QC was performed as previously described.[47] Finally, the dataset was imputed to the 1000 Genomes Project Phase 3. Linear regression models testing the top SNPs identified in the discovery cohort were calculated separately for the [^{18}F]flortaucipir and [^{18}F]MK-6240 cohorts, covaried for age, sex, *APOE* genotype, diagnosis, and ancestry PCs. Demographic and neuropsychological performance data for AIBL participants who underwent [^{18}F]flortaucipir scans can be found in **Supplementary Table 10**, while demographic and neuropsychological performance data for participants who underwent [^{18}F]MK-6240 scans can be found in **Supplementary Table 11**.

Supplementary Table 10. AIBL [^{18}F]Flortaucipir Cohort Demographics (Mean (Standard Deviation))			
	**CN (n=65)**	**MCI (n=8)**	**AD (n=4)**
Age (years)	75.0 (7.0)	74.0 (6.0)	75.0 (8.0)
Sex (M, F)	32, 34	5, 3	2, 2
Education (years)	12.5 (3.0)	9.5 (6.0)	14.5 (5.0)
Aβ Positivity (%)	39.0%	50.0%	75.0%
MMSE Total Score	28.0 (1.0)	26.5 (2.0)	22.5 (5.0)
CDR Global Score	0.0 (0.1)	0.5 (0.2)	0.8 (0.5)
CDR Sum of Boxes	0.1 (0.3)	1.1 (1.1)	4.2 (3.0)

Supplementary Table 11. AIBL [^{18}F]MK-6240 Cohort Demographics (Mean (Standard Deviation))			
	**CN (n=271)**	**MCI (n=102)**	**AD (n=85)**
Age (years)	76.0 (6.0)	74.0 (8.0)	71.0 (8.0)
Sex (M, F)	123, 156	61, 44	45, 40
Education (years)	14.0 (3.0)	12.0 (3.0)	12.0 (3.0)
Aβ Positivity (%)	19.0%	58.0%	83.0%
MMSE Total Score	28.5 (1.0)	26.5 (2.0)	22.5 (4.0)
CDR Global Score	0.0 (0.1)	0.5 (0.1)	0.8 (0.5)
CDR Sum of Boxes	0.1 (0.3)	1.3 (0.9)	5.2 (2.5)

Berkeley Aging Cohort Study (BACS): The BACS study complies with all relevant ethical regulations. Informed consent was obtained for all participants according to the Declaration of Helsinki, and studies were approved by the relevant Institutional Review Boards. The BACS began recruiting cognitively normal individuals from the community in 2005.[49,50] 107 individuals with genotyping and [^{18}F]flortaucipir were included in the analysis. [^{18}F]flortaucipir scans were collected as previously described and processed using standard techniques to generate SUVR images. Global cortical [^{18}F]flortaucipir SUVR was extracted for each scan using Freesurfer-generated ROIs. Genotype data was generated using the Illumina OmniExpress Exome panel and subjected to standard QC.[51] Data was then imputed using European samples from the HRC r1.1.216 reference panel (Build 37, Assembly 19) and filtered for quality and minor allele frequency. Finally, linear regression models were calculated to assess the relationship of the top

SNPs from the discovery cohorts to tau deposition, covaried for age, sex, *APOE* genotype, and the first two ancestry PCs. Demographic and neuropsychological performance data for BACS participants who underwent [^{18}F]flortaucipir scans can be found in **Supplementary Table 12.**

**Supplementary Table 12. BACS Demographics (Mean (Standard Deviation))**	
	**CN (n=107)**
Age (years)	70.8 (11.8)
Sex (M, F)	45, 63
Education (years)	16.8 (1.8)
APOE ε4 carrier (%)	31.0%
MMSE Total Score	29.0 (1.1)

Additional Statistical Methods

Genome-wide association analysis (GWAS): A conservative threshold for genome-wide significant association (p<5 × 10^{-8}) was employed.[52] QQman was used to generate Manhattan and Q-Q plots, and LocusZoom was used to obtain regional association plots for selected loci.[53]

Gene-set enrichment analysis: Gene-set enrichment analysis was performed using GWAS summary statistics to identify pathways and functional gene sets with significant associations with global cortical tau deposition levels using the GSA-SNP software, as described in the Supplementary information. The GSA-SNP uses a p-value of each SNP from GWAS summary statistics to test if a pathway-phenotype association is significantly different from all other pathway-phenotype associations.[54,55] Gene-set enrichment analysis was restricted to pathways containing between 10 and 200 genes. False discovery rate (FDR) with the Benjamini-Hochberg procedure was used for multiple comparison correction.[56] Significantly enriched pathways with global cortical tau levels were defined as those with FDR-corrected *p*-value<0.05.

Gene-based association analysis: Genome-wide gene-based association analysis was performed using GWAS p-values and the KGG software as described previously.[55,57] First, SNPs in each

gene were divided into different LD blocks depending on pairwise LD coefficients (r^2) for all SNPs. Second, for each block, a block-based p-value for association was calculated, and the key SNP was derived and marked. Next, the block-based p-values were combined, accounting for LD between the key SNPs using the scaled chi-square.

Interaction with diagnosis, *APOE* genotype, and amyloid positivity: Using data from ADNI, ADNI-DOD, IMAS, A4, and A05, we assessed the effect of the identified SNP (rs2113389 – dominant model (CC vs CT/TT)) and its interaction with other variables of interest on global and medial temporal lobe (MTL) tau, which includes the entorhinal cortex, fusiform, and parahippocampal gyrus and is a primary location of tau deposition in the early stages of AD. 1154 individuals, including 789 CN, 265 MCI, and 100 AD, were included in these *post-hoc* analyses (12 participants (3 CN, 8 MCI, 1 AD) were excluded for missing either *APOE* genotype or amyloid status data). The effect of the identified SNP on MTL and global tau (both rank-based inverse normal transformed) was assessed using an ANCOVA model, covaried for age, sex, *APOE* ε4 carrier status, amyloid positivity, diagnosis, and the first two population PCs. Next, we tested whether there was an interaction between the identified SNP (rs2113389 – dominant model (CC vs CT/TT)) with diagnosis on MTL, and global tau was evaluated using a two-way ANCOVA model, covaried for age, sex, *APOE* ε4 carrier status, amyloid positivity, and the first two population PCs. Similarly, the interaction effect of the identified SNP (rs2113389 – dominant model (CC vs CT/TT)) with *APOE* ε4 carrier status on MTL and global tau was evaluated using a two-way ANCOVA model, covaried for age, sex, amyloid positivity, diagnosis, and the first two population PCs. Finally, the interaction effect of the identified SNP (rs2113389 – dominant model (CC vs CT/TT)) with amyloid positivity status on MTL and global

tau was evaluated using a two-way ANCOVA model, covaried for age, sex, *APOE* ε4 carrier status, diagnosis, and the first two population PCs.

In addition, we sought to determine whether differential effects were observed in a sex-stratified analysis, given previous studies suggesting that females have higher tau deposition than males. Thus, we tested the main effect of the identified SNP (rs2113389 – dominant model (CC vs CT/TT)) on MTL and global tau, as well as its interaction with diagnostic group, *APOE* ε4 carrier status, and amyloid positivity status, in males (n=648) and females (n=506) separately.

Detailed whole-brain imaging analysis: The tau PET SUVR images were used in a voxel-wise statistical analysis of the effect of the top identified SNP on tau deposition using SPM12 (www.fil.ion.ucl.ac.uk/spm/) in a *post-hoc* analysis (n=1154). A multivariable regression analysis, masked for grey and white matter, was performed using age, sex, *APOE* ε4 carrier status, amyloid positivity, diagnosis, and the first two ancestry PCs as covariates. In the voxel-wise whole brain analysis, a voxel-wise threshold of $p<0.05$ with family-wise error (FWE) adjustment for multiple comparisons was used. Both an additive and dominant model for the identified SNP (rs2113389 – dominant model (CC vs CT/TT)) were tested.

AMP-AD bulk RNA-Seq data in the post-mortem human brain: Pre-processed bulk RNA-Seq data were downloaded from the AMP-AD Knowledge Portal (https://doi.org/10.7303/syn2580853)[58] of the Accelerating Medicines Partnership for Alzheimer's Disease (AMP-AD) Consortium:[59-63] the Mayo Clinic Brain Bank, the Mount Sinai Medical Center Brain Bank (MSBB), and the Religious Orders Study and Memory and Aging Project cohorts (ROSMAP), where RNA-Seq data were generated from the temporal cortex and cerebellum, from parahippocampal gyrus, inferior frontal gyrus, superior temporal gyrus, and frontal pole, and from dorsolateral prefrontal cortex, respectively. The procedures for sample

collection, post-mortem sample descriptions, tissue and RNA preparation methods, library preparation and sequencing methods, and sample quality controls were previously described in detail.[58,63]

Single-nucleus RNA-Seq (snRNA-Seq) analysis: Single-nucleus RNA-Seq preprocessing: Processed snRNA-Seq data from frozen brain tissue specimens (N=479) from the dorsolateral prefrontal cortex (DLFPC) from the ROSMAP cohort was downloaded from the AD Knowledge Portal (https://www.synapse.org/#!Synapse:syn31512863).[64,65] Nuclei suspension was prepared as described previously.[66] 5,000 nuclei from each of eight participants were then pooled into one sample, and the 40,000 nuclei in around 15-30 ul volume were loaded into the 10X Single Cell RNA-Seq Platform using the Chromium Single Cell 3' Reagent Kits version 3. Libraries were made following the manufacturer's protocol and sequenced on using Illumina NovaSeq 6000. All raw data are available through the AD Knowledge Portal (https://www.synapse.org/#!Synapse:syn31512863). FASTQ files were processed using the CellRanger software (10x Genomics). The "remove-background" module of CellBender (https://github.com/broadinstitute/CellBender) was used to call cells and eliminate UMI counts of ambient RNA. Our snRNA-Seq library consisted of nuclei from eight individuals. To assign back each nucleus to its participant of origin, each nucleus' genotype data obtained from the snRNA-Seq reads were compared with whole genome sequencing (WGS) SNPs of 1,196 ROS/MAP individuals using demuxlet software.[67] Among 479 specimens analyzed by snRNA-Seq, the following was excluded: specimens that failed in the genotype-based donor assignment, specimens that potentially experienced sample swaps, specimens that had quality problems in WGS, specimens with shallow sequencing depth, and specimens from duplicated individuals. After these quality control processes, 424 individuals remained (n=55 removed). Single-nucleus

RNA-Seq analysis: Using the cell-type annotations of a previous work,[66] a regularized logistic-regression classifier was constructed that infers cell type based on single-nucleus gene expression. This classifier was applied to each cell of single-nucleus libraries. Then, low-quality nuclei were determined using cell-type specific thresholds of total number of UMIs and number of unique features, and low-quality nuclei were removed. Doublets were detected by running DoubletFinder.[68] Pseudobulk UMI count matrix for each cell type was generated by extracting UMI counts of the cell type and by aggregating the counts per gene per individual. Low expression genes were filtered out by using filterByExpr function of edgeR with its default parameters. The pseudobulk counts were normalized by using the trimmed mean of M-values (TMM) method of edgeR, and $\log_2$ of counts per million mapped reads (CPM) were computed using the voom function of limma. Low expression genes whose $\log_2$CPM were less than 2.0 were filtered out. Batch effects were corrected using ComBat.

Allen Human Brain Atlas data and analysis: Regional gene expression profiles for 20,736 protein-coding genes were derived from brain-wide microarray-based transcriptome data from the Allen Human Brain Atlas (https://human.brain-map.org/microarray/search).[69,70] The microarray probes were collected from 3,700 regional brain tissue samples from autopsy data of six adult individuals (5 males/1 female; aged 24–57 years) without history of neurological disorders. Since the first two donors (1 male and 1 female) did not show interhemispheric asymmetries or sex-related differences in gene expression data,[69] the later four donors (all males) had brain tissue collection only in the left hemisphere.[70] The Desikan-Killiany (D-K) atlas was used to parcellate each gene expression map into 68 cortical ROIs.[6] The expression values from multiple probes were mean averaged for each gene. The averaged gene expression values from all voxels in each ROI were mapped into a specific cortical region. Median values were calculated for each gene and each

ROI.[71] Here, we focus on the spatial distributions of gene expression profiles in the left hemisphere genes in the identified locus, *CYP1B1* and *RMDN2*.

ADNI DNA methylation data: DNA methylation data was downloaded from the ADNI LONI (https://adni.loni.usc.edu), where Illumina EPIC chips (Illumina, Inc., San Diego, CA, USA) were used to profile DNA methylation in 1,920 blood or buffy coats samples including 200 duplicate samples according to the Illumina protocols.[72] A detailed protocol describing DNA measurement has been described previously in detail.[72-74] In brief, genomic DNA samples were obtained from NCRAD (National Centralized Repository for Alzheimer's Disease and Related Dementias), and normalization and quality control were conducted. One sample out of the total 1,920 was excluded because it had no CpG calls, and four samples were excluded because $\geq 1\%$ of the CpG sites had a detection p-value > 0.05. The remaining longitudinal 1,915 samples were normalized using the dasen method in the wateRmelon R package.[75] After normalization, quality control, and removal of duplicates, the dataset (N=1,708) of comprised beta-values was used for further analyses. We performed methylation quantitative trait loci (meQTLs) of rs2113389 with CpGs in *CYP1B1* at the first visit (N=634) using multivariate linear models adjusted for age, sex, cell composition changes, and DNA storage/source.

AD pathology mouse model analysis: hTau mouse model: The generation of hTAU mice was previously described.[76,77] The hTAU mice generated by Andorfer et al., (Jackson Laboratory #005491)[78] was crossed to *Mapt* knockout mouse (Jackson Laboratory #007251). The mice were bred and housed in specific-pathogen-free conditions. Both male and female mice were used. Animals used in the study were housed in the Stark Neurosciences Research Institute Laboratory Animal Resource Center, Indiana University School of Medicine. All animals were maintained, and experiments were performed in accordance with the recommendations in the Guide for the

Care and Use of Laboratory Animals of the National Institutes of Health. The protocol was approved by the Institutional Animal Care and Use Committee (IACUC) at Indiana University School of Medicine. Brain extraction and tissue processing: Mice were euthanized by perfusion with ice-cold phosphate-buffered saline (PBS) following full anesthetization.[79] Brains were immediately removed. The left hemisphere was dissected into cortex and hippocampus and immediately frozen in dry ice. Samples were stored at -80°C until further processing for biochemical experiments. The frozen brain tissue was homogenized in buffer consisting of 20 mM Tris-HCl (pH=7.4), 250 mM sucrose, 0.5 mM ethylene glycol-bis (β-aminoethyl ether)-N,N,N',N'-tetraacetic acid (EGTA), 0.5 mM ethylenediaminetetraacetic acid (EDTA), and RNase-free water and were stored in an equal volume of RNA STAT-60 (Amsbio) at -80°C until RNA extraction was performed. RNA was isolated by chloroform extraction and purified using the Purelink RNA Mini Kit (Life Technologies).[80] The cDNA was prepared from 750 ng of RNA using the High-Capacity of RNA-to-cDNA kit (Applied Biosystems), and qPCR was performed on the StepOne Plus Real-Time PCR system (Life Technologies) with the Taqman Gene Expression Assay (Cyp1b1; Mm00487229_m1, Life Technologies). Relative gene expression was determined with the △△CT method and was assessed relative to GAPDH (Mm99999915_g1). Student's t test was performed for qPCR results comparing C57BL/6J (B6; wild type) and hTAU mice. rTg4510 and J20 mouse model: Transgenic mice harboring human tau (rTg4510) and amyloid precursor protein (J20) mutations were used to investigate if gene expression changes of *Cyp1b1* was associated with the progression of Alzheimer's disease (AD) pathology.[81] The rTg4510 mouse model overexpresses a human mutant (P301L) form of the microtubule-associated protein tau (MAPT) for progressive tau pathology.[82,83] The J20 mouse line expresses a mutant (K670N/M671L and V717F) form of the human amyloid precursor

protein (APP) for amyloid pathology.[84,85] The experimental models and methods were described in detail in a previous publication.[81] Briefly, all animal procedures were carried out at Eli Lilly and Company. Only female mice were used in this study.[86,87] rTg4510 (rTg(tet-o-TauP301L)4510) were bred on a mixed 129S6/SvEvTac + FVB/NCrl background.[82,83] Bi-transgenic female mice and littermate controls (WT) at ages 2, 4, 6 and 8 months-old (n = 9-10 animals per group) were used for this study. J20 (B6.Cg-Zbtb20Tg(PDGFB-APPSwInd) 20Lms/2Mmjax) were bred on a C57BL/6JOlaHsd background.[85,88] Hemizygous females and littermate controls (WT) at ages 6, 8, 10 and 12 months of age (n = 9-10 animals per group) were used for this study. Histopathology: The right hemisphere from all animals was processed and embedded in paraffin wax. Negative and positive controls were used for each immunohistochemistry experiment. Tau pathology was assessed by using mouse monoclonal PG5 as the primary antibody, which recognizes tau phosphorylated at Ser409.[89,90] Tau pathology was also assessed with the mouse monoclonal AT8 primary antibody specific for tau phosphorylated at Ser202 and Thr205.[89,90] Amyloid pathology was assessed by using monoclonal biotinylated 3D6, which binds to the amino acids 1-5 in Aβ.[91] RNA isolation and highly parallel RNA sequencing: Tissue samples from each model were processed separately and individual samples were randomized to ensure that each group was equally represented in each processing batch. Total RNA from the entorhinal cortex was isolated using the AllPrep DNA/RNA Mini Kit (QIAGEN), with minor modifications to the manufacturer’s protocol. RNA quality and quantity for all samples was checked using RNA ScreenTape (Agilent). The optimal eight entorhinal cortex samples for each group were selected for transcriptional profiling (total n = 128 samples; two models (rTg4510/J20) x two groups (TG/WT) x four timepoints x eight individual animals per group). Stranded-specific mRNA sequencing libraries and cDNA libraries were prepared,

and libraries were individually cleaned up.[92] Samples were pooled together to a 2nM concentration, for subsequent sequencing. Pooled libraries were quantified, and final library pools were distributed across twelve HiSeq2500 (Illumina) lanes (six lanes for each model) and subjected to 125bp paired-end sequencing yielding a mean untrimmed read depth of ~20 million reads/sample. RNA-seq data processing: Raw files were demultiplexed into FASTQ files and checked for potential contamination. The randomized FASTQ files underwent quality control (QC) assessments using *FastQC*.[93] Trimming (ribosomal sequences removal, quality threshold 20, minimum sequence length 35) was performed with *fastqmcf* and trimmed samples were aligned to the *mm10* (GRCm38.p4) reference mouse genome using *STAR*.[94] Gene expression quantification was achieved using *featureCounts*.[95] RNA-seq gene expression analysis: Read counts were analyzed for differential expression using the R package DESeq2.[96] We were interested in detecting both genotype effects and progressive changes across age between the transgenic and wild-type samples. The following statistical model was used, including main effects for both Genotype and Age (both coded as categorical variables) and an interaction between these two terms: *f*

Gene expression = Genotype + Age + Genotype*Age

To identify significant genotype effects, a Wald test was used, and to identify significant effects of age and interaction effects (i.e., Genotype*Age), the likelihood-ratio test was used, both applied with the DESeq function from the DESeq2 package.[96] To test for associations between gene expression and measures of neuropathology quantified using immunohistochemistry, linear models using DESeq were fitted and a Wald test was used to calculate *P* values. These models were fitted separately for neuropathology data measured in the entorhinal cortex for each

individual. *P* values were adjusted for multiple testing using the false discovery rate (FDR) method.[56]

Supplementary Table 13. Gene set enrichment analysis of global cortical tau deposition

(A) List of top gene ontology (GO) pathways for global cortical tau deposition.

Pathway maps	Set size[a]	z-score	q-value
Homophilic cell adhesion via plasma membrane adhesion molecules	151(154)	6.24	1.38E-6
Regulation of Ras protein signal transduction	182(192)	6.06	2.22E-6
Ras guanyl-nucleotide exchange factor activity	123(130)	5.97	2.51E-6
Guanyl-nucleotide exchange factor activity	192(205)	5.93	2.51E-6
Plasma membrane organization	197(199)	5.80	4.46E-6
UDP-glycosyltransferase activity	128(139)	5.21	1.04E-4
Interaction with host	157(159)	5.12	1.44E-4
Flavonoid glucuronidation	21(21)	5.06	1.71E-4
Protein autophosphorylation	179(189)	5.04	1.71E-4
Regulation of Rho protein signal transduction	105(110)	5.03	1.71E-4
Positive regulation of leukocyte cell-cell adhesion	192(208)	4.99	1.81E-4
Positive regulation of homotypic cell-cell adhesion	191(207)	4.97	1.81E-4
Positive regulation of T cell activation	187(203)	4.93	2.06E-4
Protein tyrosine kinase activity	140(146)	4.84	3.00E-4
Cellular glucuronidation	25(25)	4.83	3.00E-4
Antigen binding	81(234)	4.80	3.24E-4
Calcium ion transmembrane transport	132(136)	4.67	5.69E-4
Glucuronosyltransferase activity	33(34)	4.67	5.69E-4
Divalent inorganic cation transmembrane transporter activity	155(162)	4.64	5.94E-4
Phosphatidylinositol binding	187(195)	4.64	5.94E-4
Lamellipodium	163(169)	4.58	7.18E-4
Flavonoid biosynthetic process	19(19)	4.57	7.28E-4

(B) List of top the Kyoto Encyclopedia of genes and genomes (KEGG) pathways for global cortical tau deposition level

Pathway maps	Set size[a]	z-score	q-value
Focal adhesion	194(202)	4.74	2.01E-4
Cell adhesion molecules (CAMs)	127(137)	4.71	2.01E-4
Ascorbate and aldarate metabolism	26(27)	4.51	2.02E-4
Type I diabetes mellitus	43(47)	4.24	5.23E-4
Viral myocarditis	69(76)	4.17	5.86E-4
Endocytosis	195(206)	4.09	6.72E-4

Phagosome	144(160)	4.06	6.72E-4
Allograft rejection	36(41)	4.02	6.97E-4
Bacterial invasion of epithelial cells	69(74)	3.82	1.43E-3
Porphyrin and chlorophyll metabolism	41(44)	3.72	1.88E-3
Pentose and glucuronate interconversions	29(30)	3.66	2.17E-3
ECM-receptor interaction	84(85)	3.53	3.27E-3
Calcium signaling pathway	168(179)	3.34	6.08E-3
Autoimmune thyroid disease	51(56)	3.33	6.08E-3
Renal cell carcinoma	67(71)	3.23	7.75E-3
Protein processing in endoplasmic reticulum	159(168)	3.18	8.71E-3
Type II diabetes mellitus	45(48)	3.18	8.71E-3
Steroid hormone biosynthesis	55(57)	3.16	8.71E-3
Drug metabolism - other enzymes	51(53)	3.13	8.71E-3
Complement and coagulation cascades	67(70)	3.10	9.22E-3
ABC transporters	42(45)	3.01	1.18E-2

Set size[a]: Number of genes from study data (number of genes in the pathway).

Supplementary Figures

A) Discovery dataset

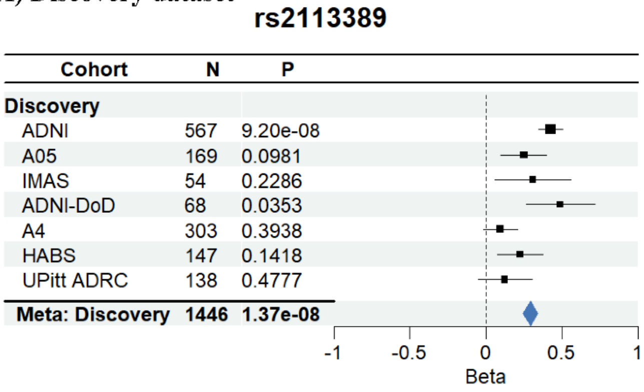

B) Replication dataset

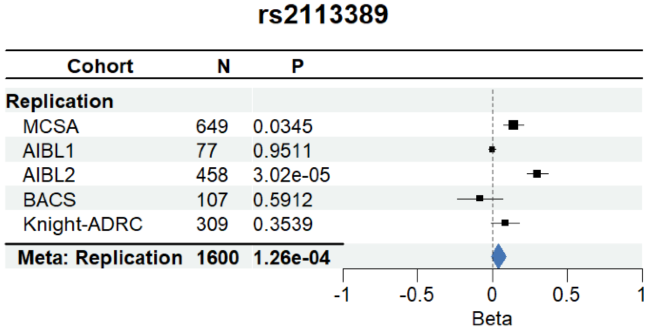

C) Combined dataset

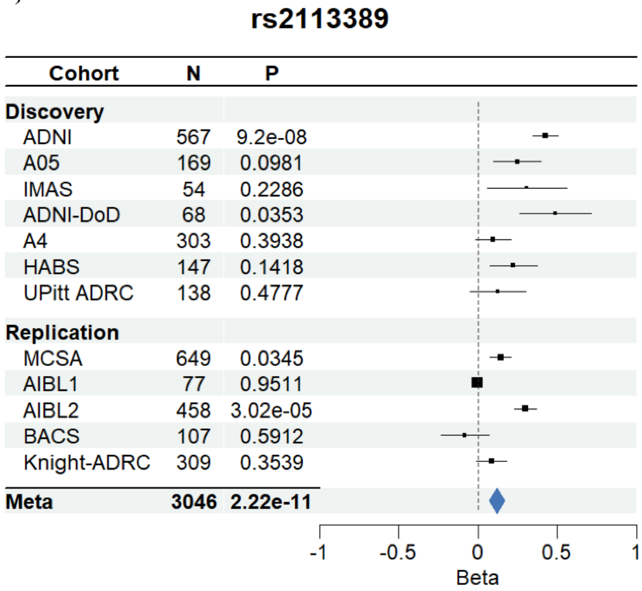

Supplementary Figure 1. Forest plots of rs2113389 across discovery, replication, and all included cohorts

A fixed effect meta-analysis with an inverse variance weighted approach was performed using METAL, and a heterogeneity analysis in METAL was performed to evaluate the possible effect of study heterogeneity on the results. In the discovery sample (A; ADNI, A05, IMAS, ADNI-DoD, HABS, UPitt ADRC), the minor allele T of rs2113389 (MAF=0.146) was associated with higher tau (Z score=5.68; *p*-value=1.37 x 10^{-8}; Heterogeneity I^2=27.8; Heterogeneity *p*-value=2.17 x 10^{-1}). We conducted a replication meta-analysis in five additional cohorts (B; n=1,687). The top SNP (rs2113389) in the discovery stage was replicated with the same association direction (Z score=3.83; *p*-value=1.26 x 10^{-4}; Heterogeneity I^2=52.0, Heterogeneity *p*-value=8.02 x 10^{-2}). The overall meta-analysis (C) showed a genome-wide significant association of rs2113389 with cortical tau deposition.

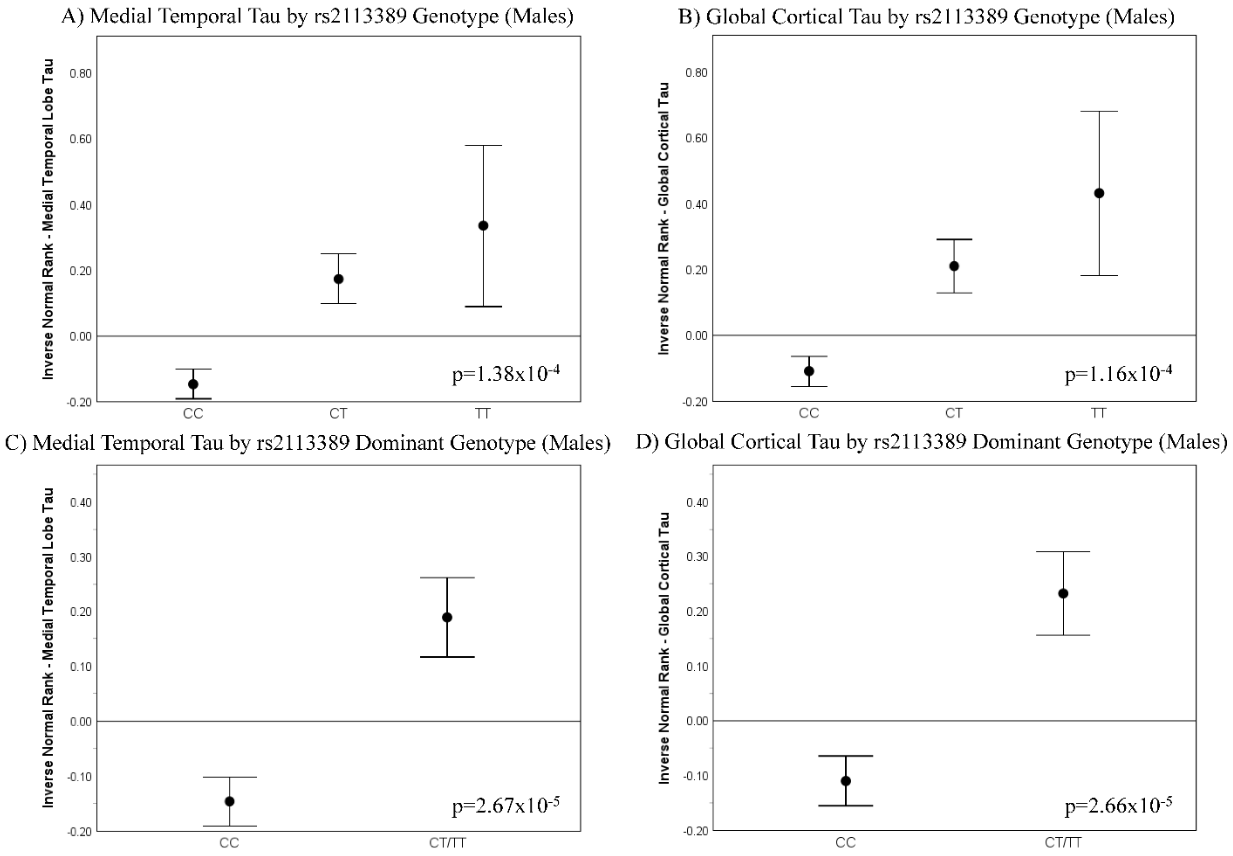

Supplementary Figure 2. Association of rs2113389 genotype with medial temporal and cortical tau deposition in males

In both additive and dominant models, rs2113389 genotype was associated with medial temporal lobe (MTL) and cortical tau deposition in males. In the additive model, CT and TT individuals showed more tau deposition in the MTL (A) and cortex (B) than CC individuals, but no differences between CT and TT groups. In the dominant model, carriers of the minor allele (T) of rs2113389 have higher MTL (C) and cortical (D) tau deposition than CC individuals. One-way ANCOVA models were used, covaried for age, diagnosis, *APOE4* carrier status, and Aβ positivity. Plots represent mean+/-standard error of the mean. Panels include 652 individuals (for panels (A-B), 482 CC, 153 CT, 17 TT; for panels (C-D), 482 CC, 170 CT/TT). Source data are

provided as a Source Data file. Aβ=amyloid-beta; ANCOVA = analysis of covariance; APOE=apolipoprotein E; MTL = medial temporal lobe; SUVR = standardized uptake value ratio. *Note: tau measured as an inverse normal transformed variable of medial temporal and cortical tau SUVR*

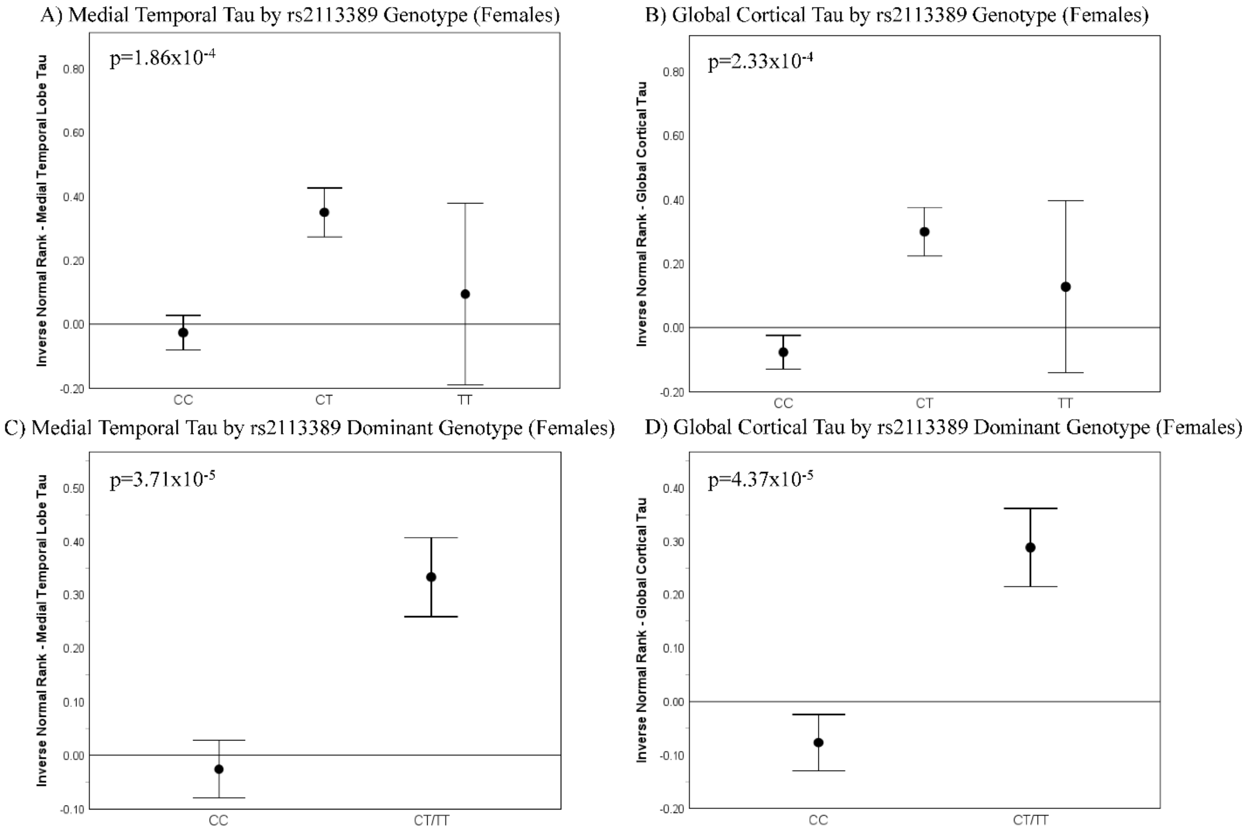

Supplementary Figure 3. Association of rs2113389 genotype with medial temporal and cortical tau deposition in females

In females, rs2113389 genotype is associated with medial temporal lobe (MTL) and cortical tau deposition. In the additive model, CT and TT individuals showed more tau deposition in the medial temporal lobe (MTL; A) and cortex (B) than CC individuals, but no differences between CT and TT groups. In the dominant model, carriers of the minor allele (T) of rs2113389 have higher MTL (C) and cortical (D) tau deposition than CC individuals. One-way ANCOVA models were used, covaried for age, diagnosis, *APOE4* carrier status, and Aβ positivity. Plots represent mean+/-standard error of the mean. Panels include 509 individuals (for panels (A-B), 352 CC, 147 CT, 10 TT; for panels (C-D), 352 CC, 157 CT/TT). Source data are provided as a Source Data file. Aβ=amyloid-beta; ANCOVA = analysis of covariance; APOE=apolipoprotein

E; MTL = medial temporal lobe; SUVR = standardized uptake value ratio. *Note: tau measured as an inverse normal transformed variable of medial temporal and cortical tau SUVR*

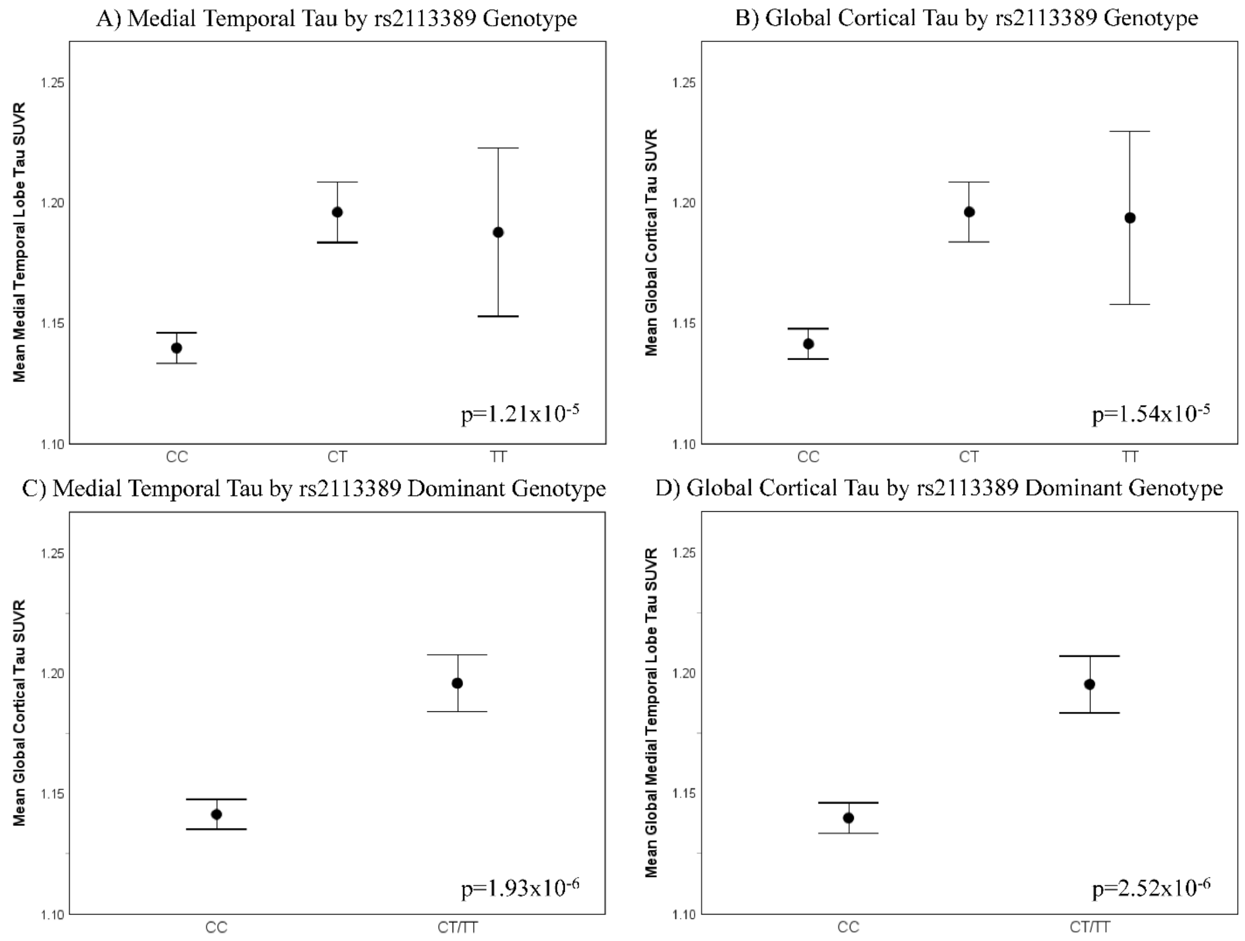

Supplementary Figure 4. Association of rs2113389 genotype with medial temporal and cortical tau deposition measured using SUVR values

Medial temporal lobe (MTL) and cortical tau SUVR was significantly associated with rs2113389 genotype. In the additive model, CT and TT individuals showed more tau deposition in the MTL (A) and cortex (B) than CC individuals, but no differences between CT and TT groups. In the dominant model, carriers of the minor allele (T) of rs2113389 have higher MTL (C) and cortical (D) tau deposition than CC individuals. One-way ANCOVA models were used, covaried for age, diagnosis, *APOE4* carrier status, and Aβ positivity. Plots represent mean+/-standard error of the mean. Panels include 1161 individuals (for (A-B), 834 CC, 300 CT, 27 TT); for (C-D), 834 CC,

327 CT/TT). Source data are provided as a Source Data file. Aβ=amyloid-beta; ANCOVA = analysis of covariance; APOE=apolipoprotein E; MTL = medial temporal lobe; SUVR = standardized uptake value ratio.

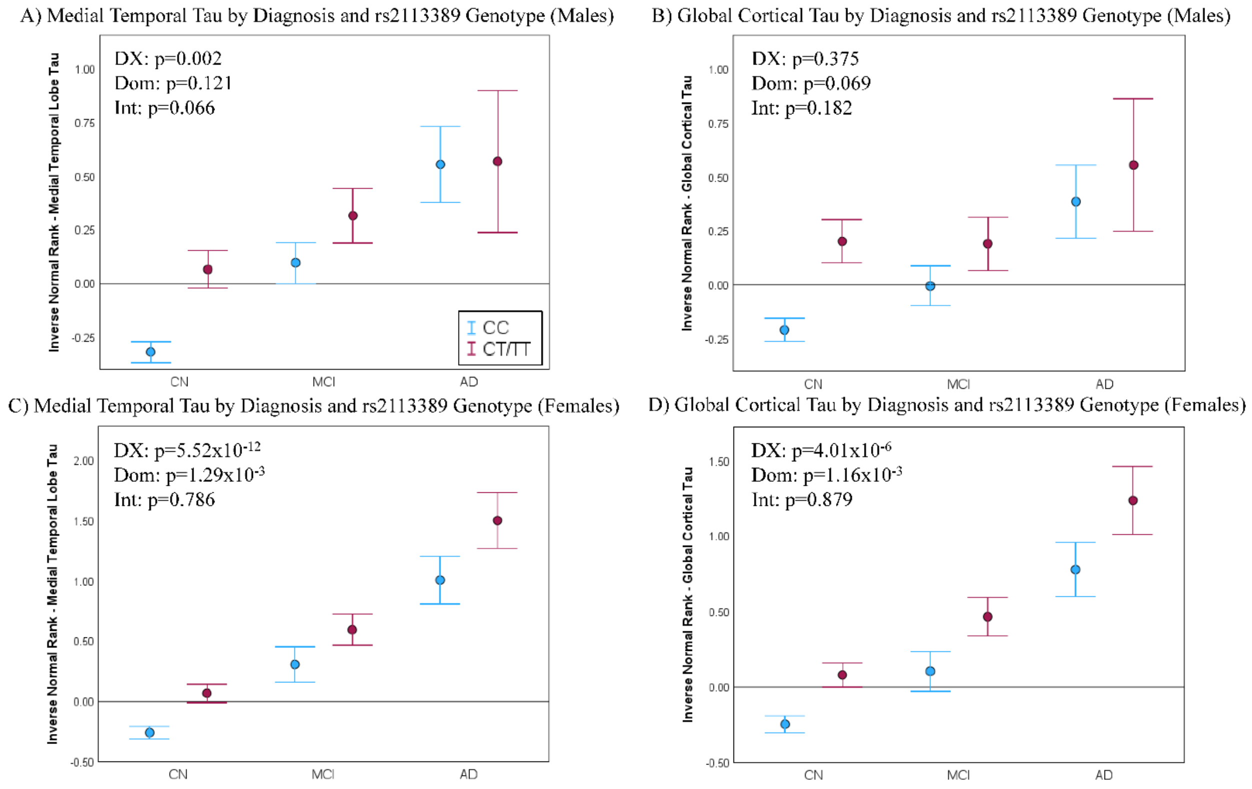

Supplementary Figure 5. Association of diagnosis and rs2113389 genotype with medial temporal and cortical tau deposition in sex-stratified samples

Significant main effects of diagnosis and rs2113389 dominant genotype were observed in females, while not generally in males. In fact, only diagnosis was only associated with tau deposition in the medial temporal lobe (MTL) in males (A), although rs2113389 dominant genotype showed a trend for a significant association with cortical tau deposition in males (B). Both diagnosis and rs2113389 dominant genotype were associated with tau in females (C-D). No significant interaction effects were observed in either males or females, although a trend for an interaction effect between rs2113389 dominant genotype and diagnosis on MTL tau was observed in males only. Two-way ANCOVA models were used, covaried for age, sex, *APOE4* carrier status, and Aβ positivity. Plots are displayed as mean+/-standard error of the mean. Panels

(A) and (B) include 652 male participants (331 CN-CC, 110 CN-CT/TT, 113 MCI-CC, 46 MCI-CT/TT, 38 AD-CC, 14 AD-CT/TT); panels (C) and (D) include female 509 participants (237 CN-CC, 112 CN-CT/TT, 82 MCI-CC, 29 MCI-CT/TT, 33 AD-CC, 16 AD-CT/TT). Source data are provided as a Source Data file. Aβ=amyloid-beta; AD=Alzheimer's disease; CN=cognitively normal; DX=diagnosis; Dom=rs2113389 dominant genotype (CC vs. CT/TT); Int.=interaction; MCI=mild cognitive impairment; MTL = medial temporal lobe; SUVR = standardized uptake value ratio. *Note: tau measured as an inverse normal transformed variable of medial temporal and cortical tau SUVR*

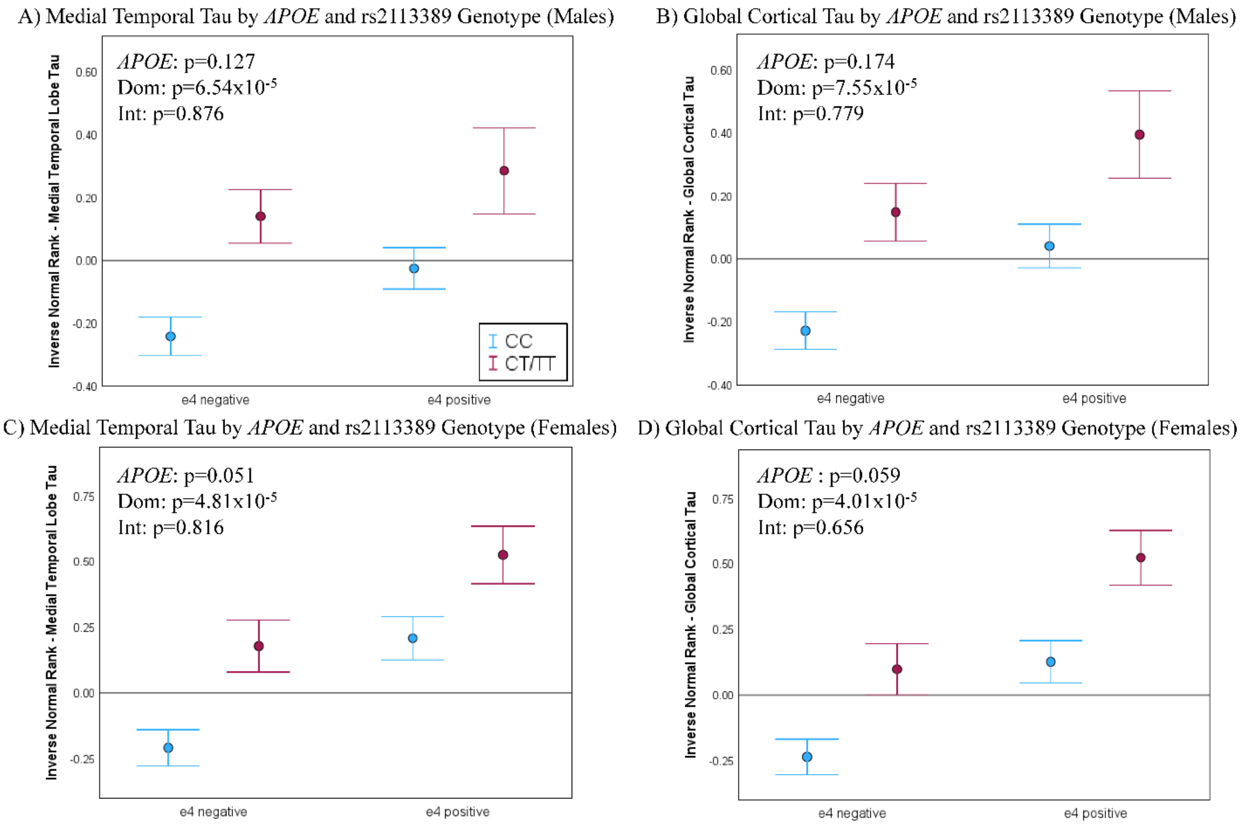

Supplementary Figure 6. Association of *APOE* ε4 carrier status and rs2113389 genotype with medial temporal and cortical tau deposition in sex-stratified samples

In both males and females, rs2113389 dominant genotype was significantly associated with medial temporal lobe (MTL) and cortical tau deposition, but *APOE4* carrier status was not significantly associated with tau deposition in the MTL or cortex. No significant interaction effects were observed in males or females. In male participants, only rs2113389 dominant genotype was associated with MTL (A) or cortical (B) tau deposition. In female participants, rs2113389 dominant genotype was significantly associated with MTL (C) and cortical (D) tau deposition, while *APOE4* carrier status showed a trend for a significant relationship with tau deposition. Two-way ANCOVA models were used, covaried for age, sex, diagnosis, and Aβ positivity. Plots are displayed as mean+/-standard error of the mean. Panels (A) and (B) include

652 male participants (270 *APOE4*-/CC, 112 *APOE4*-/CT/TT, 212 *APOE4*+/CC, 58 *APOE4*+/CT/TT); panels (C) and (D) include 509 female participants (198 *APOE4*-/CC, 87 *APOE4*-/CT/TT, 154 *APOE4*+/CC, 70 *APOE4*+/CT/TT). Source data are provided as a Source Data file. Aβ=amyloid-beta; APOE=apolipoprotein E; Dom=rs2113389 dominant genotype (CC vs. CT/TT); Int.=interaction; MTL = medial temporal lobe; SUVR = standardized uptake value ratio. *Note: tau measured as an inverse normal transformed variable of medial temporal and cortical tau SUVR*

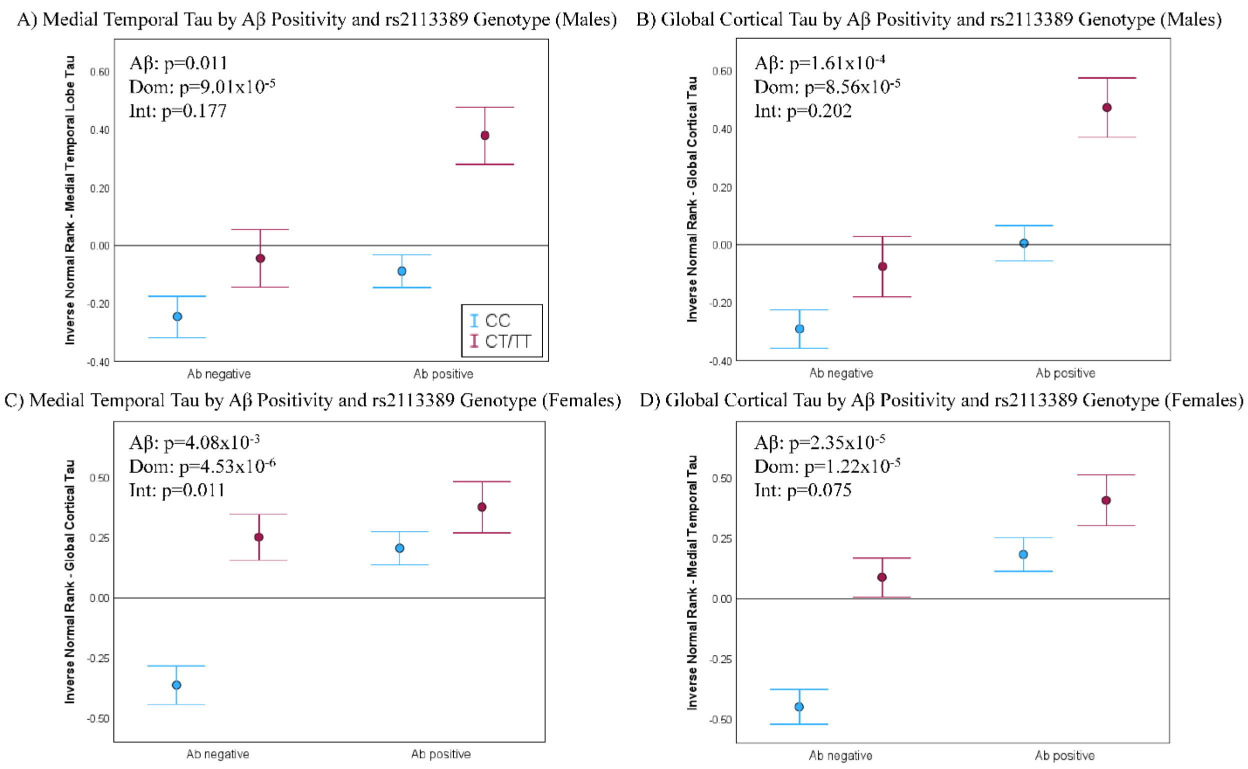

Supplementary Figure 7. Association of Aβ positivity and rs2113389 genotype with medial temporal and cortical tau deposition in sex-stratified samples

Males showed significant main effects of both Aβ positivity and rs2113389 dominant genotype on medial temporal lobe (MTL; A) and cortical (B) tau deposition, with Aβ positive individuals carrying at least one minor allele (T) at rs2113389 showing the highest level of tau deposition relative to all other groups. No interaction effect was observed between Aβ positivity and rs2113389 in males. However, in females both main effects and an interaction effect between Aβ positivity and rs2113389 were observed. Specifically, Aβ positive females carrying at least one minor allele (T) at rs2113389 showed the highest levels of tau in the MTL (C) and cortex (D) relative to all other groups. An interaction effect was also seen, which was significant in the MTL and showed trend significance in the cortex. Two-way ANCOVA models were used, covaried for age, sex, diagnosis, and *APOE4* carrier status. Plots are displayed as mean+/-

standard error of the mean. Panels (A) and (B) include include 648 participants (194 Aβ-/CC, 71 Aβ-/CT/TT, 285 Aβ+/CC, 98 Aβ+/CT/TT; panels (C) and (D) include 506 participants (144 Aβ-/CC, 60 Aβ-/CT/TT, 206 Aβ+/CC, 96 Aβ+/CT/TT). Source data are provided as a Source Data file. Aβ=amyloid-beta; APOE=apolipoprotein E; Dom=rs2113389 dominant genotype (CC vs. CT/TT); Int.=interaction; MTL = medial temporal lobe; SUVR = standardized uptake value ratio. *Note: tau measured as an inverse normal transformed variable of medial temporal and cortical tau SUVR*

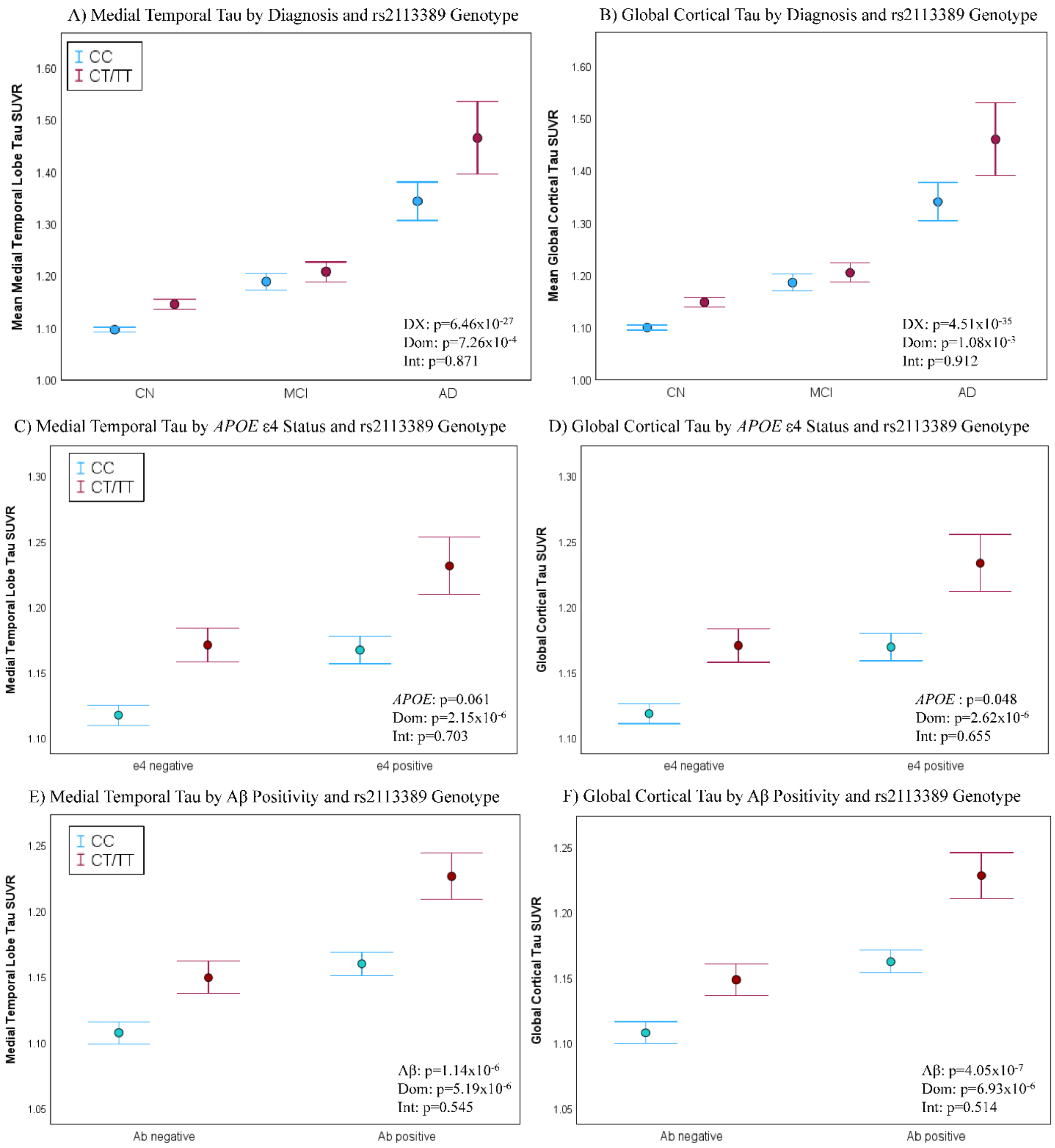

Supplementary Figure 8. Interaction effect of rs2113389 with diagnosis, *APOE4* carrier status, and Aβ positivity on regional and cortical tau SUVR

Both diagnosis and rs2113389 dominant genotype were significantly associated with medial temporal lobe (MTL; A) and cortical (B) tau SUVR, such that AD participants who carry at least one minor allele (T) of rs2113389 have the highest tau level relative to all other diagnoses and CC rs2113389 individuals. *APOE4* carrier status and rs2113389 dominant genotype are also significantly associated with MTL (C) and cortical (D) tau SUVR, although *APOE4* carrier status showed only trend level significance for the MTL. The highest tau level is observed in carriers of both at least one *APOE4* allele and minor allele (T) at rs2113389, relative to either CC rs2113389 individuals or *APOE4* negative individuals. Finally, significant effects of both Aβ positivity and rs2113389 dominant genotype on medial temporal (E) and cortical (F) tau SUVR are observed, with Aβ+ individuals carrying at least one minor allele (T) at rs2113389, showing the highest level of tau deposition relative to all other groups (Aβ- individuals, rs2113389 CC individuals). Two-way ANCOVA models, covaried for age and sex, as well as diagnosis, *APOE4* carrier status, and Aβ positivity where appropriate, were used. Plots are displayed as mean+/-standard error of the mean. Panels (A) and (B) include 1161 participants (568 CN-CC, 222 CN-CT/TT, 195 MCI-CC, 75 MCI-CT/TT, 71 AD-CC, 30 AD-CT/TT); panels (C) and (D) include 1161 participants (468 *APOE4*-/CC, 199 *APOE4*-/CT/TT, 366 *APOE4*+/CC, 128 *APOE4*+/CT/TT); panels (E) and (F) include 1154 participants (338 Aβ-/CC, 131 Aβ-/CT/TT, 491 Aβ+/CC, 194 Aβ+/CT/TT). Source data are provided as a Source Data file. Aβ=amyloid-beta; AD=Alzheimer's disease; ANCOVA = analysis of covariance; APOE=apolipoprotein E; CN=cognitively normal; DX=diagnosis; Dom=rs2113389 dominant genotype (CC vs. CT/TT); Int.=interaction; MCI=mild cognitive impairment; MTL = medial temporal lobe; SUVR = standardized uptake value ratio.

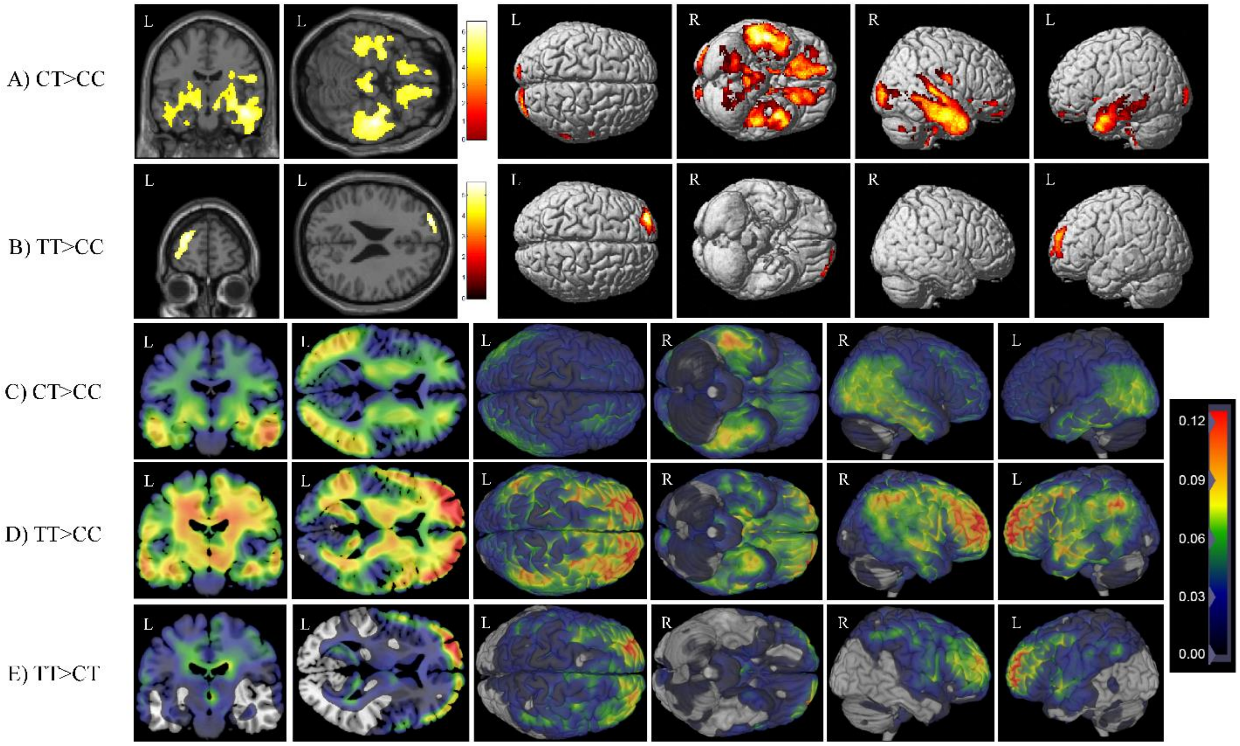

Supplementary Figure 9. Exploratory voxel-wise analysis of the effect of rs2113389 additive genotype on tau deposition

Using an additive model, the voxel-wise statistical maps of tau deposition showed that individuals with (A) rs2113389 CT genotype demonstrated significantly higher tau deposition in the inferior frontal, parietal, and medial and lateral temporal lobes relative to those with a rs2113389 CC genotype. (B) Individuals with a rs2113389 TT genotype also showed more tau deposition than those with a rs2113389 CC genotype in a focal region in the left frontal cortex. No significant differences were seen between rs2113389 CT and TT genotype individuals on voxel-wise statistical maps. The latter two findings were likely limited topographically due to the small sample size of the TT group. Images are displayed at a voxel-wise threshold of $p<0.05$ with family-wise error correction for multiple comparisons and a minimum cluster size (k)=100 voxels. Due to the very unequal group sizes, we also evaluated non-statistically limited beta-map

value maps. (C) Beta-value maps suggest that rs2113389 CT heterozygotes show higher tau deposition than CC homozygotes in primarily parietal and temporal regions. (D) Alternatively, the beta-value maps suggest that rs2113389 TT homozygotes have higher tau in widespread regions of the temporal and parietal lobes, as well as strongly in the bilateral frontal lobes, relative to rs2113389 CC homozygotes. (E) Finally, beta-value maps suggested that rs2113389 TT homozygotes show greater bilateral frontal tau than rs2113389 CT heterozygotes. One-way ANCOVA models were used, covaried for age, sex, diagnosis, *APOE4* carrier status, and Aβ positivity. Analyses include 1154 individuals (829 CC, 298 CT, 27 TT). Aβ=amyloid-beta; ANCOVA = analysis of covariance; APOE=apolipoprotein E.

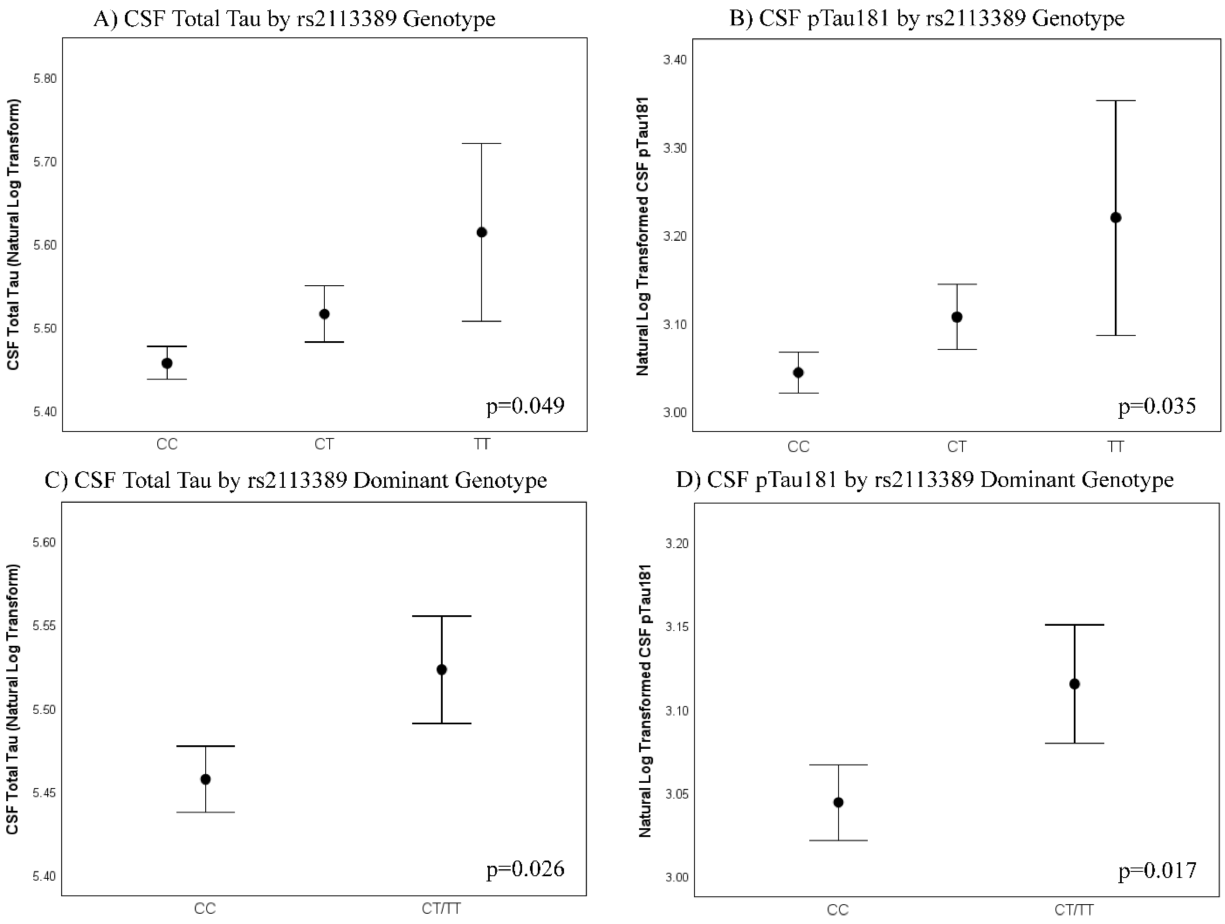

Supplementary Figure 10. Association of rs2113389 genotype with CSF total tau and phosphorylated tau 181 (pTau181)

In the ADNI and ADNI-DoD cohorts, CSF total tau (A) and pTau181 (B) were significantly associated with rs2113389 genotype in an additive model. The dominant model showed similar results, with carriers of the rs2113389 T-allele showing higher CSF total tau (C) and pTau181 (D) levels than non-carriers. One-way ANCOVA models were used, covaried for age, sex, diagnosis, *APOE4* carrier status, and Aβ positivity. Plots represent mean+/-standard error of the mean. Panels (A) and (C) include 528 individuals (for (A), 353 CC, 163 CT, 12 TT; for (C), 353 CC, 175 CT/TT), while panels (B) and (D) include 525 individuals (for (B), 352 CC, 161 CT, 12 TT; for (D), 352 CC, 173 CT/TT). Source data are provided as a Source Data file. Aβ=amyloid-

beta; ADNI = Alzheimer's Disease Neuroimaging Initiative; ADNI-DoD = ADNI - Department of Defense; ANCOVA = analysis of covariance; APOE=apolipoprotein E; CSF = cerebrospinal fluid; pTau181 = tau phosphorylated at threonine-181. *Note: CSF tau measures were not normally distributed and thus, were transformed with a natural log prior to analysis*.

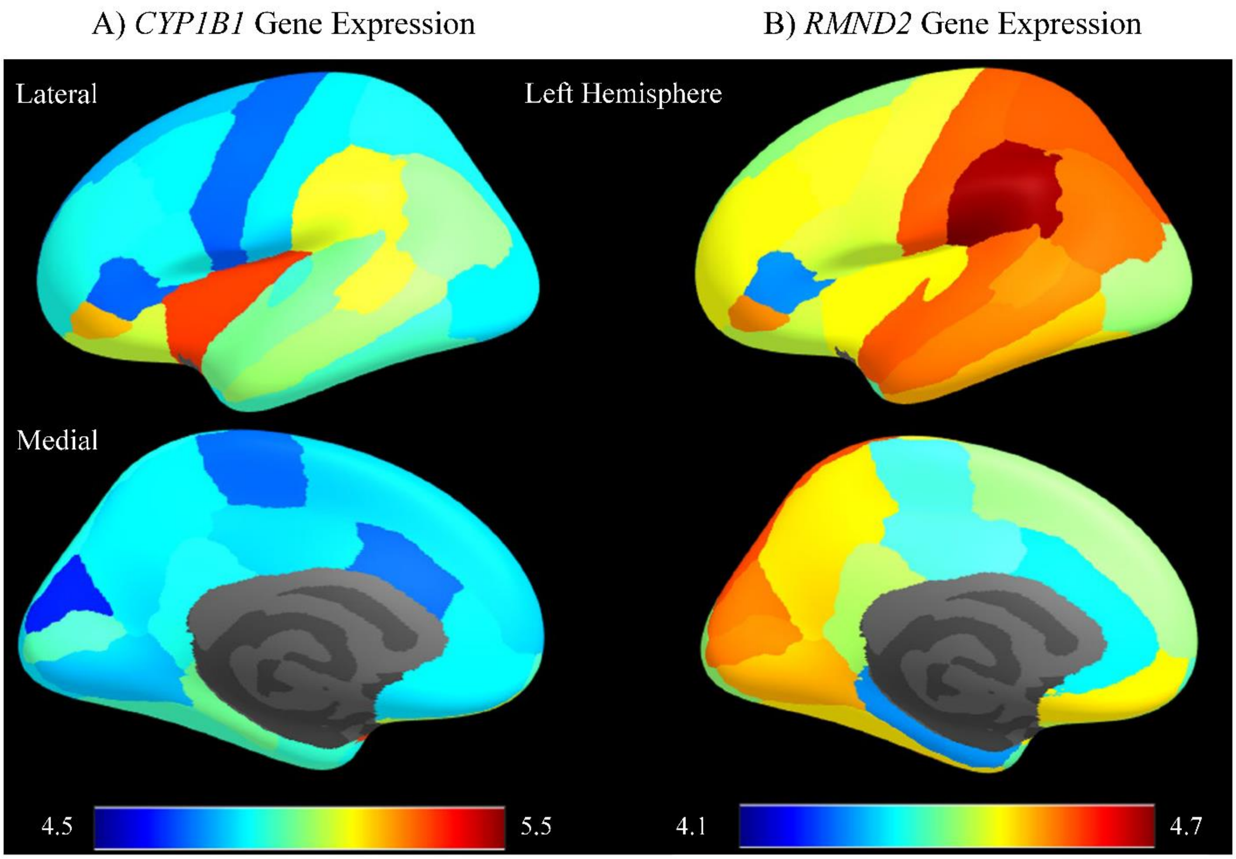

Supplementary Figure 11. Spatial distributions of *CYP1B1* and *RMDN2* gene expression profiles in the left hemisphere

The lateral (top) and medial (bottom) brain views for *CYP1B1* (A) and *RMDN2* (B) gene expression in the Allen Human Brain Atlas are shown. *CYP1B1* shows high gene expression in the insula, orbitofrontal cortex, temporal lobe, and medial temporal lobe. *RMDN2* shows strong gene expression levels in the temporal lobe, visual cortex, frontal (medial orbitofrontal cortex) and posterior (precuneus and isthmus cingulate cortex) default mode network regions, and sensorimotor cortex. Of note, the regions showing high gene expression levels for both genes partly overlap with the typical regions with high tau deposition in AD. AD = Alzheimer's disease

Supplementary References

1 Weiner, M. W. *et al.* Impact of the Alzheimer's Disease Neuroimaging Initiative, 2004 to 2014. *Alzheimers Dement* **11**, 865-884 (2015). https://doi.org/10.1016/j.jalz.2015.04.005

2 Weiner, M. W. & Veitch, D. P. Introduction to special issue: Overview of Alzheimer's Disease Neuroimaging Initiative. *Alzheimers Dement* **11**, 730-733 (2015). https://doi.org/10.1016/j.jalz.2015.05.007

3 Weiner, M. W. *et al.* The Alzheimer's Disease Neuroimaging Initiative 3: Continued innovation for clinical trial improvement. *Alzheimers Dement* **13**, 561-571 (2017). https://doi.org/10.1016/j.jalz.2016.10.006

4 Veitch, D. P. *et al.* Understanding disease progression and improving Alzheimer's disease clinical trials: Recent highlights from the Alzheimer's Disease Neuroimaging Initiative. *Alzheimers Dement* **15**, 106-152 (2019). https://doi.org/10.1016/j.jalz.2018.08.005

5 Weiner, M. W. *et al.* The Alzheimer's Disease Neuroimaging Initiative 3: Continued innovation for clinical trial improvement. *Alzheimers Dement* **13**, 561-571 (2017). https://doi.org/10.1016/j.jalz.2016.10.006

6 Desikan, R. S. *et al.* An automated labeling system for subdividing the human cerebral cortex on MRI scans into gyral based regions of interest. *Neuroimage* **31**, 968-980 (2006). https://doi.org/10.1016/j.neuroimage.2006.01.021

7 Klunk, W. E. *et al.* The Centiloid Project: standardizing quantitative amyloid plaque estimation by PET. *Alzheimers Dement* **11**, 1-15 e11-14 (2015). https://doi.org/10.1016/j.jalz.2014.07.003

8 Risacher, S. L. *et al.* Olfactory identification in subjective cognitive decline and mild cognitive impairment: Association with tau but not amyloid positron emission tomography. *Alzheimers Dement (Amst)* **9**, 57-66 (2017). https://doi.org/10.1016/j.dadm.2017.09.001

9 Risacher, S. L. *et al.* Head injury is associated with tau deposition on PET in MCI and AD patients. *Alzheimers Dement (Amst)* **13**, e12230 (2021). https://doi.org/10.1002/dad2.12230

10 Weiner, M. W. *et al.* Effects of traumatic brain injury and posttraumatic stress disorder on development of Alzheimer's disease in Vietnam Veterans using the Alzheimer's Disease Neuroimaging Initiative: Preliminary Report. *Alzheimers Dement (N Y)* **3**, 177-188 (2017). https://doi.org/10.1016/j.trci.2017.02.005

11 McKhann, G. M. *et al.* The diagnosis of dementia due to Alzheimer's disease: recommendations from the National Institute on Aging-Alzheimer's Association workgroups on diagnostic guidelines for Alzheimer's disease. *Alzheimers Dement* **7**, 263-269 (2011). https://doi.org/10.1016/j.jalz.2011.03.005

12 Petersen, R. C. Mild cognitive impairment as a diagnostic entity. *J Intern Med* **256**, 183-194 (2004). https://doi.org/10.1111/j.1365-2796.2004.01388.x

13 Trzepacz, P. T. *et al.* Relationship between the Montreal Cognitive Assessment and Mini-mental State Examination for assessment of mild cognitive impairment in older adults. *BMC Geriatr* **15**, 107 (2015). https://doi.org/10.1186/s12877-015-0103-3

14 Pontecorvo, M. J. *et al.* A multicentre longitudinal study of flortaucipir (18F) in normal ageing, mild cognitive impairment and Alzheimer's disease dementia. *Brain : a journal of neurology* **142**, 1723-1735 (2019). https://doi.org/10.1093/brain/awz090

15 Pontecorvo, M. J. *et al.* Relationships between flortaucipir PET tau binding and amyloid burden, clinical diagnosis, age and cognition. *Brain : a journal of neurology* **140**, 748-763 (2017). https://doi.org/10.1093/brain/aww334

16 Sperling, R. A. *et al.* Association of Factors With Elevated Amyloid Burden in Clinically Normal Older Individuals. *JAMA Neurol* **77**, 735-745 (2020). https://doi.org/10.1001/jamaneurol.2020.0387
17 Sperling, R. A. *et al.* The A4 study: stopping AD before symptoms begin? *Sci Transl Med* **6**, 228fs213 (2014). https://doi.org/10.1126/scitranslmed.3007941
18 Lee, Y. *et al.* Genetic variation affecting exon skipping contributes to brain structural atrophy in Alzheimer's disease. *AMIA Jt Summits Transl Sci Proc* **2017**, 124-131 (2018).
19 Kim, S. *et al.* Genome-wide association study of CSF biomarkers Abeta1-42, t-tau, and p-tau181p in the ADNI cohort. *Neurology* **76**, 69-79 (2011). https://doi.org/10.1212/WNL.0b013e318204a397
20 Price, A. L. *et al.* Principal components analysis corrects for stratification in genome-wide association studies. *Nat Genet* **38**, 904-909 (2006). https://doi.org/10.1038/ng1847
21 Nho, K. *et al.* Whole-exome sequencing and imaging genetics identify functional variants for rate of change in hippocampal volume in mild cognitive impairment. *Mol Psychiatry* **18**, 781-787 (2013). https://doi.org/10.1038/mp.2013.24
22 Farrell, M. E. *et al.* Association of Emerging β-Amyloid and Tau Pathology With Early Cognitive Changes in Clinically Normal Older Adults. *Neurology* **98**, e1512-e1524 (2022). https://doi.org/10.1212/wnl.0000000000200137
23 Dagley, A. *et al.* Harvard Aging Brain Study: Dataset and accessibility. *Neuroimage* **144**, 255-258 (2017). https://doi.org/10.1016/j.neuroimage.2015.03.069
24 Yang, H. S. *et al.* An UNC5C Allele Predicts Cognitive Decline and Hippocampal Atrophy in Clinically Normal Older Adults. *J Alzheimers Dis* **68**, 1161-1170 (2019). https://doi.org/10.3233/jad-180788
25 Johnson, K. A. *et al.* Tau positron emission tomographic imaging in aging and early Alzheimer disease. *Annals of neurology* **79**, 110-119 (2016). https://doi.org/10.1002/ana.24546
26 Buckley, R. F. *et al.* Sex Differences in the Association of Global Amyloid and Regional Tau Deposition Measured by Positron Emission Tomography in Clinically Normal Older Adults. *JAMA Neurol* **76**, 542-551 (2019). https://doi.org/10.1001/jamaneurol.2018.4693
27 Snitz, B. E. *et al.* Associations between NIH Toolbox Cognition Battery and in vivo brain amyloid and tau pathology in non-demented older adults. *Alzheimers Dement (Amst)* **12**, e12018 (2020). https://doi.org/10.1002/dad2.12018
28 Petersen, R. C. *et al.* Prevalence of mild cognitive impairment is higher in men. The Mayo Clinic Study of Aging. *Neurology* **75**, 889-897 (2010). https://doi.org/10.1212/WNL.0b013e3181f11d85
29 Roberts, R. O. *et al.* The Mayo Clinic Study of Aging: design and sampling, participation, baseline measures and sample characteristics. *Neuroepidemiology* **30**, 58-69 (2008). https://doi.org/10.1159/000115751
30 Ramanan, V. K. *et al.* Variants in PPP2R2B and IGF2BP3 are associated with higher tau deposition. *Brain Commun* **2**, fcaa159 (2020). https://doi.org/10.1093/braincomms/fcaa159
31 Jack, C. R. *et al.* The bivariate distribution of amyloid-β and tau: relationship with established neurocognitive clinical syndromes. *Brain : a journal of neurology* **142**, 3230-3242 (2019). https://doi.org/10.1093/brain/awz268
32 Jack, C. R., Jr. *et al.* Longitudinal tau PET in ageing and Alzheimer's disease. *Brain : a journal of neurology* **141**, 1517-1528 (2018). https://doi.org/10.1093/brain/awy059
33 Jack, C. R., Jr. *et al.* Brain β-amyloid load approaches a plateau. *Neurology* **80**, 890-896 (2013). https://doi.org/10.1212/WNL.0b013e3182840bbe

34 Schwarz, C. G. *et al.* A Comparison of Partial Volume Correction Techniques for Measuring Change in Serial Amyloid PET SUVR. *J Alzheimers Dis* **67**, 181-195 (2019). https://doi.org/10.3233/jad-180749
35 Ramanan, V. K. *et al.* Tau polygenic risk scoring: a cost-effective aid for prognostic counseling in Alzheimer's disease. *Acta Neuropathol* **143**, 571-583 (2022). https://doi.org/10.1007/s00401-022-02419-2
36 Brier, M. R. *et al.* Tau and Aβ imaging, CSF measures, and cognition in Alzheimer's disease. *Sci Transl Med* **8**, 338ra366 (2016). https://doi.org/10.1126/scitranslmed.aaf2362
37 Suárez-Calvet, M. *et al.* Early increase of CSF sTREM2 in Alzheimer's disease is associated with tau related-neurodegeneration but not with amyloid-β pathology. *Mol Neurodegener* **14**, 1 (2019). https://doi.org/10.1186/s13024-018-0301-5
38 Deming, Y. *et al.* Genome-wide association study identifies four novel loci associated with Alzheimer's endophenotypes and disease modifiers. *Acta Neuropathol* **133**, 839-856 (2017). https://doi.org/10.1007/s00401-017-1685-y
39 Maxwell, T. J. *et al.* Genome-wide association study for variants that modulate relationships between cerebrospinal fluid amyloid-beta 42, tau, and p-tau levels. *Alzheimers Res Ther* **10**, 86 (2018). https://doi.org/10.1186/s13195-018-0410-y
40 Mishra, S. *et al.* AV-1451 PET imaging of tau pathology in preclinical Alzheimer disease: Defining a summary measure. *Neuroimage* **161**, 171-178 (2017). https://doi.org/10.1016/j.neuroimage.2017.07.050
41 Guvakova, M. A. Improving patient classification and biomarker assessment using Gaussian Mixture Models and Bayes' rule. *Oncoscience* **6**, 383-385 (2019). https://doi.org/10.18632/oncoscience.494
42 Delaneau, O. & Marchini, J. Integrating sequence and array data to create an improved 1000 Genomes Project haplotype reference panel. *Nat Commun* **5**, 3934 (2014). https://doi.org/10.1038/ncomms4934
43 Howie, B., Fuchsberger, C., Stephens, M., Marchini, J. & Abecasis, G. R. Fast and accurate genotype imputation in genome-wide association studies through pre-phasing. *Nat Genet* **44**, 955-959 (2012). https://doi.org/10.1038/ng.2354
44 Abecasis, G. R. *et al.* An integrated map of genetic variation from 1,092 human genomes. *Nature* **491**, 56-65 (2012). https://doi.org/10.1038/nature11632
45 Chang, C. C. *et al.* Second-generation PLINK: rising to the challenge of larger and richer datasets. *Gigascience* **4**, 7 (2015). https://doi.org/10.1186/s13742-015-0047-8
46 Ellis, K. A. *et al.* The Australian Imaging, Biomarkers and Lifestyle (AIBL) study of aging: methodology and baseline characteristics of 1112 individuals recruited for a longitudinal study of Alzheimer's disease. *Int Psychogeriatr* **21**, 672-687 (2009). https://doi.org/10.1017/s1041610209009405
47 Fowler, C. *et al.* Fifteen Years of the Australian Imaging, Biomarkers and Lifestyle (AIBL) Study: Progress and Observations from 2,359 Older Adults Spanning the Spectrum from Cognitive Normality to Alzheimer's Disease. *J Alzheimers Dis Rep* **5**, 443-468 (2021). https://doi.org/10.3233/adr-210005
48 Doré, V. *et al.* Plasma p217+tau versus NAV4694 amyloid and MK6240 tau PET across the Alzheimer's continuum. *Alzheimers Dement (Amst)* **14**, e12307 (2022). https://doi.org/10.1002/dad2.12307
49 Schöll, M. *et al.* PET Imaging of Tau Deposition in the Aging Human Brain. *Neuron* **89**, 971-982 (2016). https://doi.org/10.1016/j.neuron.2016.01.028

50 Perrotin, A., Mormino, E. C., Madison, C. M., Hayenga, A. O. & Jagust, W. J. Subjective Cognition and Amyloid Deposition Imaging: A Pittsburgh Compound B Positron Emission Tomography Study in Normal Elderly Individuals. *Archives of Neurology* **69**, 223-229 (2012). https://doi.org/10.1001/archneurol.2011.666
51 Raghavan, N. S. *et al.* Association Between Common Variants in RBFOX1, an RNA-Binding Protein, and Brain Amyloidosis in Early and Preclinical Alzheimer Disease. *JAMA Neurol* **77**, 1288-1298 (2020). https://doi.org/10.1001/jamaneurol.2020.1760
52 Kanai, M., Tanaka, T. & Okada, Y. Empirical estimation of genome-wide significance thresholds based on the 1000 Genomes Project data set. *J Hum Genet* **61**, 861-866 (2016). https://doi.org/10.1038/jhg.2016.72
53 Pruim, R. J. *et al.* LocusZoom: regional visualization of genome-wide association scan results. *Bioinformatics* **26**, 2336-2337 (2010). https://doi.org/10.1093/bioinformatics/btq419
54 Nam, D., Kim, J., Kim, S. Y. & Kim, S. GSA-SNP: a general approach for gene set analysis of polymorphisms. *Nucleic Acids Res* **38**, W749-754 (2010). https://doi.org/10.1093/nar/gkq428
55 Horgusluoglu-Moloch, E. *et al.* Genome-wide association analysis of hippocampal volume identifies enrichment of neurogenesis-related pathways. *Sci Rep* **9**, 14498 (2019). https://doi.org/10.1038/s41598-019-50507-3
56 Benjamini, Y. & Hochberg, Y. Controlling the False Discovery Rate - a Practical and Powerful Approach to Multiple Testing. *J R Stat Soc B* **57**, 289-300 (1995). https://doi.org/DOI 10.1111/j.2517-6161.1995.tb02031.x
57 Li, M. X., Gui, H. S., Kwan, J. S. & Sham, P. C. GATES: a rapid and powerful gene-based association test using extended Simes procedure. *Am J Hum Genet* **88**, 283-293 (2011). https://doi.org/10.1016/j.ajhg.2011.01.019
58 Wan, Y. W. *et al.* Meta-Analysis of the Alzheimer's Disease Human Brain Transcriptome and Functional Dissection in Mouse Models. *Cell Rep* **32**, 107908 (2020). https://doi.org/10.1016/j.celrep.2020.107908
59 Allen, M. *et al.* Human whole genome genotype and transcriptome data for Alzheimer's and other neurodegenerative diseases. *Sci Data* **3**, 160089 (2016). https://doi.org/10.1038/sdata.2016.89
60 De Jager, P. L. *et al.* A multi-omic atlas of the human frontal cortex for aging and Alzheimer's disease research. *Sci Data* **5**, 180142 (2018). https://doi.org/10.1038/sdata.2018.142
61 Wang, M. *et al.* The Mount Sinai cohort of large-scale genomic, transcriptomic and proteomic data in Alzheimer's disease. *Sci Data* **5**, 180185 (2018). https://doi.org/10.1038/sdata.2018.185
62 Wan, Y. W. *et al.* Meta-Analysis of the Alzheimer's Disease Human Brain Transcriptome and Functional Dissection in Mouse Models. *Cell Reports* **32** (2020). https://doi.org/ARTN 107908

10.1016/j.celrep.2020.107908
63 Logsdon, B. A. *et al.* Meta-analysis of the human brain transcriptome identifies heterogeneity across human AD coexpression modules robust to sample collection and methodological approach. *bioRxiv*, 510420 (2019). https://doi.org/10.1101/510420
64 Bennett, D. A. *et al.* Religious Orders Study and Rush Memory and Aging Project. *J Alzheimers Dis* **64**, S161-S189 (2018). https://doi.org/10.3233/JAD-179939
65 Fujita, M. *et al.* Cell subtype-specific effects of genetic variation in the Alzheimer's disease brain. *Nat Genet* **56**, 605-614 (2024). https://doi.org/10.1038/s41588-024-01685-y
66 Cain, A. *et al.* Multi-cellular communities are perturbed in the aging human brain and with Alzheimer’s disease. *bioRxiv*, 2020.2012.2022.424084 (2020). https://doi.org/10.1101/2020.12.22.424084

67 Kang, H. M. *et al.* Multiplexed droplet single-cell RNA-sequencing using natural genetic variation. *Nat Biotechnol* **36**, 89-94 (2018). https://doi.org/10.1038/nbt.4042
68 McGinnis, C. S., Murrow, L. M. & Gartner, Z. J. DoubletFinder: Doublet Detection in Single-Cell RNA Sequencing Data Using Artificial Nearest Neighbors. *Cell Syst* **8**, 329-337.e324 (2019). https://doi.org/10.1016/j.cels.2019.03.003
69 Hawrylycz, M. J. *et al.* An anatomically comprehensive atlas of the adult human brain transcriptome. *Nature* **489**, 391-399 (2012). https://doi.org/10.1038/nature11405
70 Hawrylycz, M. *et al.* Canonical genetic signatures of the adult human brain. *Nat Neurosci* **18**, 1832-1844 (2015). https://doi.org/10.1038/nn.4171
71 French, L. & Paus, T. A FreeSurfer view of the cortical transcriptome generated from the Allen Human Brain Atlas. *Front Neurosci* **9**, 323 (2015). https://doi.org/10.3389/fnins.2015.00323
72 Vasanthakumar, A. *et al.* Harnessing peripheral DNA methylation differences in the Alzheimer's Disease Neuroimaging Initiative (ADNI) to reveal novel biomarkers of disease. *Clin Epigenetics* **12**, 84 (2020). https://doi.org/10.1186/s13148-020-00864-y
73 Kim, J. P. *et al.* Integrative Co-methylation Network Analysis Identifies Novel DNA Methylation Signatures and Their Target Genes in Alzheimer's Disease. *Biol Psychiatry* (2022). https://doi.org/10.1016/j.biopsych.2022.06.020
74 Li, Q. S. *et al.* Association of peripheral blood DNA methylation level with Alzheimer's disease progression. *Clin Epigenetics* **13**, 191 (2021). https://doi.org/10.1186/s13148-021-01179-2
75 Pidsley, R. *et al.* A data-driven approach to preprocessing Illumina 450K methylation array data. *BMC Genomics* **14**, 293 (2013). https://doi.org/10.1186/1471-2164-14-293
76 Komuro, Y., Xu, G., Bhaskar, K. & Lamb, B. T. Human tau expression reduces adult neurogenesis in a mouse model of tauopathy. *Neurobiol Aging* **36**, 2034-2042 (2015). https://doi.org/10.1016/j.neurobiolaging.2015.03.002
77 Bemiller, S. M. *et al.* TREM2 deficiency exacerbates tau pathology through dysregulated kinase signaling in a mouse model of tauopathy. *Molecular Neurodegeneration* **12**, 74 (2017). https://doi.org/10.1186/s13024-017-0216-6
78 Andorfer, C. *et al.* Hyperphosphorylation and aggregation of tau in mice expressing normal human tau isoforms. *J Neurochem* **86**, 582-590 (2003). https://doi.org/10.1046/j.1471-4159.2003.01879.x
79 Jadhav, V. S. *et al.* Trem2 Y38C mutation and loss of Trem2 impairs neuronal synapses in adult mice. *Molecular Neurodegeneration* **15**, 62 (2020). https://doi.org/10.1186/s13024-020-00409-0
80 Tsai, A. P. *et al.* PLCG2 is associated with the inflammatory response and is induced by amyloid plaques in Alzheimer’s disease. *Genome Medicine* **14**, 17 (2022). https://doi.org/10.1186/s13073-022-01022-0
81 Castanho, I. *et al.* Transcriptional Signatures of Tau and Amyloid Neuropathology. *Cell Rep* **30**, 2040-2054 e2045 (2020). https://doi.org/10.1016/j.celrep.2020.01.063
82 Ramsden, M. *et al.* Age-dependent neurofibrillary tangle formation, neuron loss, and memory impairment in a mouse model of human tauopathy (P301L). *J Neurosci* **25**, 10637-10647 (2005). https://doi.org/10.1523/JNEUROSCI.3279-05.2005
83 Santacruz, K. *et al.* Tau suppression in a neurodegenerative mouse model improves memory function. *Science* **309**, 476-481 (2005). https://doi.org/10.1126/science.1113694
84 Hsia, A. Y. *et al.* Plaque-independent disruption of neural circuits in Alzheimer's disease mouse models. *Proc Natl Acad Sci U S A* **96**, 3228-3233 (1999). https://doi.org/10.1073/pnas.96.6.3228
85 Mucke, L. *et al.* High-level neuronal expression of abeta 1-42 in wild-type human amyloid protein precursor transgenic mice: synaptotoxicity without plaque formation. *J Neurosci* **20**, 4050-4058 (2000).

86 Blackmore, T. *et al.* Tracking progressive pathological and functional decline in the rTg4510 mouse model of tauopathy. *Alzheimers Res Ther* **9**, 77 (2017). https://doi.org/10.1186/s13195-017-0306-2

87 Yue, M., Hanna, A., Wilson, J., Roder, H. & Janus, C. Sex difference in pathology and memory decline in rTg4510 mouse model of tauopathy. *Neurobiol Aging* **32**, 590-603 (2011). https://doi.org/10.1016/j.neurobiolaging.2009.04.006

88 Harris, J. A. *et al.* Many neuronal and behavioral impairments in transgenic mouse models of Alzheimer's disease are independent of caspase cleavage of the amyloid precursor protein. *J Neurosci* **30**, 372-381 (2010). https://doi.org/10.1523/JNEUROSCI.5341-09.2010

89 Jicha, G. A. *et al.* cAMP-dependent protein kinase phosphorylations on tau in Alzheimer's disease. *J Neurosci* **19**, 7486-7494 (1999).

90 Ahmed, Z. *et al.* A novel in vivo model of tau propagation with rapid and progressive neurofibrillary tangle pathology: the pattern of spread is determined by connectivity, not proximity. *Acta Neuropathol* **127**, 667-683 (2014). https://doi.org/10.1007/s00401-014-1254-6

91 Demattos, R. B. *et al.* A plaque-specific antibody clears existing beta-amyloid plaques in Alzheimer's disease mice. *Neuron* **76**, 908-920 (2012). https://doi.org/10.1016/j.neuron.2012.10.029

92 Risso, D., Ngai, J., Speed, T. P. & Dudoit, S. Normalization of RNA-seq data using factor analysis of control genes or samples. *Nat Biotechnol* **32**, 896-902 (2014). https://doi.org/10.1038/nbt.2931

93 Nho, K. *et al.* Integration of bioinformatics and imaging informatics for identifying rare PSEN1 variants in Alzheimer's disease. *BMC Med Genomics* **9 Suppl 1**, 30 (2016). https://doi.org/10.1186/s12920-016-0190-9

94 Dobin, A. *et al.* STAR: ultrafast universal RNA-seq aligner. *Bioinformatics* **29**, 15-21 (2013). https://doi.org/10.1093/bioinformatics/bts635

95 Liao, Y., Smyth, G. K. & Shi, W. featureCounts: an efficient general purpose program for assigning sequence reads to genomic features. *Bioinformatics* **30**, 923-930 (2014). https://doi.org/10.1093/bioinformatics/btt656

96 Love, M. I., Huber, W. & Anders, S. Moderated estimation of fold change and dispersion for RNA-seq data with DESeq2. *Genome Biol* **15**, 550 (2014). https://doi.org/10.1186/s13059-014-0550-8

Consortia

Alzheimer's Disease Neuroimaging Initiative (ADNI)

Michael Weiner, Paul Aisen, Ronald Petersen, Clifford R. Jack Jr, William Jagust, John Q. Trojanowki, Arthur W. Toga, Laurel Beckett, Robert C. Green, Andrew J. Saykin, John C. Morris, Leslie M. Shaw, Enchi Liu, Tom Montine, Ronald G. Thomas, Michael Donohue, Sarah Walter, Devon Gessert, Tamie Sather, Gus Jiminez, Danielle Harvey, Matthew Bernstein, Nick Fox, Paul Thompson, Norbert Schuff, Charles DeCArli, Bret Borowski, Jeff Gunter, Matt Senjem, Prashanthi Vemuri, David Jones, Kejal Kantarci, Chad Ward, Robert A. Koeppe, Norm Foster, Eric M. Reiman, Kewei Chen, Chet Mathis, Susan Landau, Nigel J. Cairns, Erin Householder, Lisa Taylor Reinwald, Virginia Lee, Magdalena Korecka, Michal Figurski, Karen Crawford, Scott Neu, Tatiana M. Kim, Steven Potkin, Li Shen, Kelly Faber, Sungeun Kim, Kwangsik Nho, Zaven Kachaturian, Richard Frank, Peter J. Snyder, Susan Molchan, Jeffrey Kaye, Joseph Quinn, Betty Lind, Raina Carter, Sara Dolen, Lon S. Schneider, Sonia Pawluczyk, Mauricio Beccera, Liberty Teodoro, Bryan M. Spann, James Brewer, Helen Vanderswag, Adam Fleisher, Judith L. Heidebrink, Joanne L. Lord, Sara S. Mason, Colleen S. Albers, David Knopman, Kris Johnson, Rachelle S. Doody, Javier Villanueva Meyer, Munir Chowdhury, Susan Rountree, Mimi Dang, Yaakov Stern, Lawrence S. Honig, Karen L. Bell, Beau Ances, Maria Carroll, Sue Leon, Erin Householder, Mark A. Mintun, Stacy Schneider, Angela OliverNG, Randall Griffith, David Clark, David Geldmacher, John Brockington, Erik Roberson, Hillel Grossman, Effie Mitsis, Leyla deToledo-Morrell, Raj C. Shah, Ranjan Duara, Daniel Varon, Maria T. Greig, Peggy Roberts, Marilyn Albert, Chiadi Onyike, Daniel D'Agostino II, Stephanie Kielb, James E. Galvin, Dana M. Pogorelec, Brittany Cerbone, Christina A. Michel, Henry Rusinek, Mony J. de Leon, Lidia Glodzik, Susan De Santi, P. Murali Doraiswamy, Jeffrey R. Petrella, Terence Z. Wong, Steven E. Arnold, Jason H. Karlawish, David A. Wolk, Charles D. Smith, Greg Jicha, Peter Hardy, Partha Sinha, Elizabeth Oates, Gary Conrad, Oscar L. Lopez, MaryAnn Oakley, Donna M. Simpson, Anton P. Porsteinsson, Bonnie S. Goldstein, Kim Martin, Kelly M. Makino, M. Saleem Ismail, Connie Brand, Ruth A. Mulnard, Gaby Thai, Catherine Mc Adams Ortiz, Kyle Womack, Dana Mathews, Mary Quiceno, Ramon Diaz Arrastia, Richard King, Myron Weiner, Kristen Martin Cook, Michael DeVous, Allan I. Levey, James J. Lah, Janet S. Cellar, Jeffrey M. Burns, Heather S. Anderson, Russell H. Swerdlow, Liana Apostolova, Kathleen Tingus, Ellen Woo, Daniel H. S. Silverman, Po H. Lu, George Bartzokis, Neill R. Graff Radford, Francine ParfittH, Tracy Kendall, Heather Johnson, Martin R. Farlow, Ann Marie Hake, Brandy R. Matthews, Scott Herring, Cynthia Hunt, Christopher H. van Dyck, Richard E. Carson, Martha G. MacAvoy, Howard Chertkow, Howard Bergman, Chris Hosein, Sandra Black, Bojana Stefanovic, Curtis Caldwell, Ging Yuek Robin Hsiung, Howard Feldman, Benita Mudge, Michele Assaly Past, Andrew Kertesz, John Rogers, Dick Trost, Charles Bernick, Donna Munic, Diana Kerwin, Marek Marsel Mesulam, Kristine Lipowski, Chuang Kuo Wu, Nancy Johnson, Carl Sadowsky, Walter Martinez, Teresa Villena, Raymond Scott Turner, Kathleen Johnson, Brigid Reynolds, Reisa A. Sperling, Keith A. Johnson, Gad Marshall, Meghan Frey, Jerome Yesavage, Joy L. Taylor, Barton Lane, Allyson Rosen, Jared Tinklenberg, Marwan N. Sabbagh, Christine M. Belden, Sandra A. Jacobson, Sherye A. Sirrel, Neil Kowall, Ronald Killiany, Andrew E. Budson, Alexander Norbash, Patricia Lynn Johnson, Thomas O. Obisesan, Saba Wolday, Joanne Allard, Alan Lerner, Paula Ogrocki, Leon Hudson, Evan Fletcher, Owen Carmichael, John Olichney, Charles DeCarli, Smita Kittur, Michael Borrie, T. Y. Lee, Rob Bartha, Sterling Johnson, Sanjay Asthana, Cynthia M. Carlsson, Steven G. Potkin, Adrian Preda,

Dana Nguyen, Pierre Tariot, Adam Fleisher, Stephanie Reeder, Vernice Bates, Horacio Capote, Michelle Rainka, Douglas W. Scharre, Maria Kataki, Anahita Adeli, Earl A. Zimmerman, Dzintra Celmins, Alice D. Brown, Godfrey D. Pearlson, Karen Blank, Karen Anderson, Robert B. Santulli, Tamar J. Kitzmiller, Eben S. Schwartz, Kaycee M. SinkS, Jeff D. Williamson, Pradeep Garg, Franklin Watkins, Brian R. Ott, Henry Querfurth, Geoffrey Tremont, Stephen Salloway, Paul Malloy, Stephen Correia, Howard J. Rosen, Bruce L. Miller, Jacobo Mintzer, Kenneth Spicer, David Bachman, Elizabether Finger, Stephen Pasternak, Irina Rachinsky, John Rogers, Andrew Kertesz, Dick Drost, Nunzio Pomara, Raymundo Hernando, Antero Sarrael, Susan K. Schultz, Laura L. Boles Ponto, Hyungsub Shim, Karen Elizabeth Smith, Norman Relkin, Gloria Chaing, Lisa Raudin, Amanda Smith, Kristin Fargher & Balebail Ashok Raj

Department of Defense Alzheimer's Disease Neuroimaging Initiative (DoD-ADNI)

Paul Aisen, Ronald C. Petersen, Michael W. Weiner

Anti-Amyloid Treatment in Asymptomatic Alzheimer's Study (A4 Study) and Longitudinal Evaluation of Amyloid Risk and Neurodegeneration (LEARN)

Reisa Sperling, Paul Aisen, Roy Yaari, Cheryl A. Brown, John R. Si, Michael Donohue, Mike Rafii, Rema Raman, Robert Rissman, Cecily Jenkins, Gustavo Jimenez-Maggiora, Jeremy Pizzola, Alison Belsha, Clifford Jack Jr, Jason Karlawish, Joshua Grill, James B. Brewer, Mark Mintun, Karen Holdridge, Isabella Velona, Kenneth Marek, John Seibyl, Marybeth Howlett, Paul Maruff, Keith Johnson, Aaron Schultz, Dorene Rentz, Kate Papp, Beth Mormino, Rebecca Amariglio, Gad Marshall, Dylan Kirn, Michael Properzi, J. Alex Becker, Rachel Buckley, Maria Arampatzidou, Alison Belsha, Jennifer Salazar, Paula Cohen, Jorge Gutierrez, Harriett Davey, Melissa Ruiz, Isabel Francis, Michael Rafii, Gabriela Muranevici, Tiffany Chow, Steve Bruno III, Gina Garcia-Camilo, Adriana Bohorquez, Alyssa Carroll (Schmitt), Sarah Danowski, Deborah Tobias, Jeremy Pizzola, Lindsey Earp, Dan Abinsay, Sarah Walter, Ryoko Ihara, Cecily Jenkins, Xavier Salazar, Stefanie Juliano, Sarah Espinoza, Shelley Moore, Taylor Clanton, Agnes Lewandowski, Gustavo Jimenez-Maggiora, Phuoc Hong, Hongmei Qiu, Jia-shing So, Stefania Bruschi, Kimberlee Eudy, Quin Revel, Michael Selsnik, Olusegun Adegoke, Veasna Tan, Olga Baryshnikava, Iris Sim Dacio, Sandhya Niranjan Jaiswal, Elizabeth Shaffer, Michelle Pablo, Rema Raman, Michael Donohue, Karin Ernstrom, Gopalan Sethuraman, Jiyoon Choi, Oliver Langford, Shunran Wang, Andy Liu, Barbara Bartocci, Melissa KorbaRobert Rissman, Sara Abdel-Latif, Andrea Abram, Bhavna Madduri, Cheryl A. Brown, Dillon Hilderbrand, Isabella Velona, John Brad Holmes, John R. Sims, Julie Chandler, Karen Holdridge, Keith Parsons, Lisa Ferguson-Sells, Mark Mintun, Marybeth Devine, Marybeth Howlett, Michael Case, Michele Mancini, Michael Pontecorvo, Peter Fairfield, Vedeline Torreon, Marianne Manire, Renarda Jones, Phyllis Ferrell, Roy Yaari, Satina Hall, Scott Kaiser, Sergey Shcherbinin, Stacy Huckins, Susan Warner, Jude Burger, Bret J. Borowski, Petrice M. Cogswell, Cory A. Johnson, Kejal Kantarci, Leonard C. Matoush Jr., William C. Turke, Ashritha L. Reddy, Denise A. Reyes, Kaely B. Thostenson, Samantha M. Zuk, Jason Karlawish, Jeffrey M. Burns, Joshua D. Grill, David Sultzer, Howard Feldman, Genny Matthews, Stephanie Parks, Jen Mason, Jason Young, Ashlee Heldreth, Renarda Jones, Janet Kastelan, Rebecca Ryan-Jones, Lindsay Cotton, Maria Bulger Lennox, Ronelyn Chavez, Tilman Oltersdorf, Curtis Taylor, Barbara LaPlante, Meghan Stirn, Joanne Brechlin, Gina Varner, Carol Evans, Karim Hussein, Erika Wilson, Ronald Thomas, Sheila Jin, James Barlow, James B. Brewer, Nichol Ferng, Robin

Jennings, Leonardino Digma, Aaron Schultz, J. Alex Becker, Heidi Jacobs, Jorge Sepulcre, Michael Properzi, Justin Sanchez, Matthew Scott, Kenneth Marek, John Seibyl, Reneé Tschopp, Donna Miles, Paul Maruff, Amy Frederickson, François Windels, Bodil Hook, Lenny Nuciforo, Natalia Contreras, Patrick McCabe, Becky Watt Knight, Rachel Griffith, Melissa McGue, Shannon Conti, Amy Martin Vogt, Marissa C. Natelson Love, P. Denise Ledlow, Amber Watkins, David S. Geldmacher, Loren Brown Ashley, Jacqueline Vaughn, William J. Burke, Roma Patel, Daniel Viramontes Apodaca, Sachin Y. Pandya, Anna D. Burke, Edward Zamrini, Zoran Obradov, Christine M. Belden, Carol Cline, Margaret Rich, Lisa Roye, Marwan Sabbagh, Jerome Yesavage, Steven Z. Chao, Tamara Beale, Jaila Coleman, Shawn Kile, Valentina Mikhalenko, Yvonne Au, Mary Vaughn, Sampreet Moneski, Tammy Donnell, Dawn Lenakakis, John Gregory Duffy, Lorrie Bisesi, Poonam Nina Banerjee, Maria Gonzalez, Rania Bilwani, John Olichney, Charles DeCarli, Hongzheng Zhang, Antoinette Lopez, Mary McPhail-Ciufo, Adrian Preda, Andrea Weideman, Steven Potkin, Melanie Tallakson, Catherine McAdams-Ortiz, Beatriz Vides, Gaby T. Thai, Steven P. Tam, Maryam Beigi, Thao Rodriguez, Maya Farchi, Roberto Hernandez, Lorena Monserratt, Lauren Garcia, Celine Ossinalde, Douglas Galasko, Helen Vanderswag, Laura Linares, Chi Kim, Shawnees Peacock, Adam L. Boxer, Lawren Vandevrede, Peter Ljubenkov, Julio C. Rojas, Mauricio Becerra, Liberty Teodoro, Sonia Pawluczyk, Karen Dagerman, Lon Schneider, Christopher H. van Dyck, Julia W. McDonald, Susan P. Good, Joanna E. Harris, Kara Bates, Jessica Lam, Raymond Scott Turner, Melanie Chadwick, Kathleen Johnson, Brigid Reynolds, Kelly McCann, Thomas O. Obisesan, Oyonumo E. Ntekim, Sheeba R. Nadarajah, Sharlene Leong, Saba Wolday, Jillian Turner, Mark Brody, Paayal Patel, Cynthia Stimeck, Neill R. Graff-Radford, Christopher Homa, Amanda Phillips, Paul Winner, Alfonso X. Moreno, Esteban Olivera, Jennifer West, Noureen Dhanani, Maria Amy Edridge, Alisa Petit, Yarnick Mirjah, Olivia Reilly, Angela Okolie, Uzma Khan, Elma Fallejo, Amanda G. Smith, Juris Jarvis, Kelly Rodrigo, Ijeoma Mba, Anna D. Sladky, Patricia Lowe, Breanna Davis, Ranjan Duara, Maria T. Greig Custo, Rosemarie A. Rodriguez, Julieth Formosa, Warren Barker, Joyce Lee, James J. Lah, Allan I. Levey, Deborah Westover, Gail Schwartz, Lauren Mariotti, Jeffrey Ross, Linda Rice, Sandra Weintraub, Ian Grant, Brittanie Muse, Shea Gold, Jelena Pejic, Loreece Haddad, Neelum T. Aggarwal, Ajay Sood, Kimberly Blanchard, Peter Lambiotis, Amelia Troutman, Martin Farlow, Jared Brosch, Nancy McClaskey, Del D. Miller, Hristina K. Koleva, Karen Ekstam Smith, Laura Temple, Susan Schultz, Jeffrey M. Burns, Anne Arthur, Rachel Starr, Nicole Mathis, Phyllis Switzer, Gregory A. Jicha, Andrea L. Shaffer, Sarah Hatch, Sierra Fuhrmann, Molly Harper, Kelly Parsons, Jeffrey N. Keller, William P. Gahan, Robert Brouillette, Heather Foil, Owen Carmichael, Paul B. Rosenberg, Meghan Schultz, Samantha Schwartz, Samantha Horn, Mersania Jn Pierre, Robert A. Stern, Jane Mwicigi, Alexa Puleio, Jesse Mez, Wendy Qiu, Eric Steinberg, Gad A. Marshall, Tia Hall, Emily Sprague, Mariana Palou, Martha Vander Vliet, Dorene M. Rentz, Jaimie Ziolkowski, Judith L. Heidebrink, Bekkie Wang, David S. Knopman, Bronwyn Briseno, Jonathan Graff Radford, Sara Mason, Karen Kuntz, Kari Baxter, Randall Bateman, Joy Snider, Gregory Day, Nupur Ghoshal, Erik Musiek, Tammie Benzinger, John Morris, Marta Santos, Daniel L. Murman, Haley Kampschneider, Deb Heimes, Nick Miller, Dylan Wint, Charles Bernick, Michelle Torreliza, Simrit Saraon, Barnett Shpritz, Karen L. Bell, Ruth Tejeda, Chrismary De La Cruz, Lawrence Honig, Betina Idnay, Horacio A. Capote, Michelle Rainka, Traci Aladeen, Tatiana Jimenez-Knight, HeatherMacNamara, Mary Sano, Judith Neugroschl, Joanne Lim, Allison Ardolino, Gina Garcia Camilo, Amy Aloyisi, Melanie Shulman, Anaztasia Ulysse, Jamika Singleton-

Garvin, Mohammed Sheikh, Mrunaliniash Gaikwad, Anton P. Porsteinsson, Audrey Rice, Susan Salem-Spencer, Bridget Holvey, Asa Widman, Michael Lin, Norman Relkin, Suzanne Craft, Abigail Heston O'Connell, Alexis Webb, Bevan Hoover, Patricia Wittmer, Alan J. Lerner, Maria Toth, Parianne Fatica, Susie Sami, Paula Ogrocki, Marianne Sanders, Michael Karathanos, Christy Lisenbee, Sarah Land, Carmen Toegel, Aimee L. Pierce, Lisa C. Silbert, Jeffrey A. Kaye, Alexandra A. Ruhf, Amy B. Thomas, Steven Aurich, G. Peter Gliebus, Katherine Rife, Melinda Webster, Christine Barr, Monica Mazurek, Jason Karlawish, Sanjeev N. Vaishnavi, Martha Combs, Jade Uffelman, Loren Terrill, Oscar Lopez, Thomas Baumgartner, Sarah Goldberg, Donna Simpson, Cary Zik, Stephen P. Salloway, Diane Monast, Vanessa Rua, Jessica Alber, Athene K.W. Lee, Sophia Tarro, Brian R. Ott, Chuang-Kuo Wu, Lori A. Daiello, Jonathan D. Drake, Alisa Omert, Hannah Alaimo, Ralph H. Johnson, Jacobo Mintzer, Olga Brawman-Mintzer, Allison Acree, Heather Allen, Arthur Williams, Sydney O'Connor, Valory Pavlik, Melissa Yu, Shayla Yonce, Joseph C. Masdeu, Belen Pascual, Mica Mangibin, Benjamin Batista, Brendan Kelley, Shahera Ranjha, Jana Windsor, Mary Quiceno, Elaine Peskind, James O'Connell, Adam McPartlin, Murray A. Raskind, Anita Ranta, Cynthia M. Carlsson, Benjamin Farral, Kim Peterson, Sandra Harding, Aleshia Cole, Sarah Best, Rebecca Shostak, Kayla VanderPloeg, Elsa Mann, Julia Truemner, Sandra Black, Benjamin Lam, Chinthaka Heyn, Samantha Paul-Stotz, Maryna Butenko, Sharon Cohen, Ian Cohen, Atif Shaikh, Ellen Buchman, Barathy Tharmalingam, Linda Schlesinger, Robin Hsiung, Ellen Kim, Tahlee Marian, Haakon Nygaard, Benita Mudge, Michele Assaly, Colin L. Masters, Andrew Huynh, Mclin Tres, Paul Yates, Georgios Zisis, Laura Marginson, Takeshi Iwatsubo, Atushi Iwata, Kazushi Suzuki, Yoshiki Niimi, Ken-ichiro Sato

Australian Imaging Biomarkers and Lifestyle (AIBL)

Colin L. Masters, Ashley Bush, Liang Jin, Scott Ayton, Kevin Taddei, Qiao-Xin Li, Paul Maruff, Jo Robertson, Christopher C. Rowe, Jurgen Fripp, Ralph Martins, Stephanie Rainey-Smith, Belinda Brown, Simon M. Laws, Tenielle Porter, Larry Ward
