# Supplementary material for: CYP1B1-RMDN2 Alzheimer’s disease endophenotype locus identified for cerebral tau PET
Source: Nat Commun. 2024 Sep 20;15:8251. doi: 10.1038/s41467-024-52298-2 (PMC11415491; doi:10.1038/s41467-024-52298-2)
Supplement: Supplementary file 3 — Reporting Summary [file 41467_2024_52298_MOESM3_ESM.pdf]

nature portfolio

Corresponding author(s): Andrew J. Saykin

Last updated by author(s): Jun 25, 2024

Reporting Summary

Nature Portfolio wishes to improve the reproducibility of the work that we publish. This form provides structure for consistency and transparency in reporting. For further information on Nature Portfolio policies, see our Editorial Policies and the Editorial Policy Checklist.

Statistics

For all statistical analyses, confirm that the following items are present in the figure legend, table legend, main text, or Methods section.

n/a	Confirmed	
☐	☒	The exact sample size (n) for each experimental group/condition, given as a discrete number and unit of measurement
☐	☒	A statement on whether measurements were taken from distinct samples or whether the same sample was measured repeatedly
☐	☒	The statistical test(s) used AND whether they are one- or two-sided<br>*Only common tests should be described solely by name; describe more complex techniques in the Methods section.*
☐	☒	A description of all covariates tested
☐	☒	A description of any assumptions or corrections, such as tests of normality and adjustment for multiple comparisons
☐	☒	A full description of the statistical parameters including central tendency (e.g. means) or other basic estimates (e.g. regression coefficient) AND variation (e.g. standard deviation) or associated estimates of uncertainty (e.g. confidence intervals)
☐	☒	For null hypothesis testing, the test statistic (e.g. F, t, r) with confidence intervals, effect sizes, degrees of freedom and P value noted<br>*Give P values as exact values whenever suitable.*
☒	☐	For Bayesian analysis, information on the choice of priors and Markov chain Monte Carlo settings
☐	☒	For hierarchical and complex designs, identification of the appropriate level for tests and full reporting of outcomes
☐	☒	Estimates of effect sizes (e.g. Cohen's d, Pearson's r), indicating how they were calculated

Our web collection on statistics for biologists contains articles on many of the points above.

Software and code

Policy information about availability of computer code

Data collection	RedCap, https://www.project-redcap.org/
Data analysis	All analyses were produced with standard software freely available online. PLINK software, https://www.cog-genomics.org/plink/; Michigan Imputation Server, https://imputationserver.sph.umich.edu/index.html#!pages/home; LocusZoom software, http://locuszoom.org/; GSA-SNP software, https://sourceforge.net/projects/gsasnp2/; KGG software, http://pmglab.top/kgg/; SPM12 software, https://www.fil.ion.ucl.ac.uk/spm/software/spm12/; Synapse database, https://www.synapse.org/; eQTLGen Consortium, https://www.eqtlgen.org/; ADNI LONI, https://adni.loni.usc.edu/; ROS/MAP cohort, https://www.radc.rush.edu/; Allen Human Brain Atlas, https://portal.brain-map.org/

For manuscripts utilizing custom algorithms or software that are central to the research but not yet described in published literature, software must be made available to editors and reviewers. We strongly encourage code deposition in a community repository (e.g. GitHub). See the Nature Portfolio guidelines for submitting code & software for further information.

Data

Policy information about availability of data

All manuscripts must include a data availability statement. This statement should provide the following information, where applicable:

- Accession codes, unique identifiers, or web links for publicly available datasets
- A description of any restrictions on data availability
- For clinical datasets or third party data, please ensure that the statement adheres to our policy

Summary statistics will be made available in the GWAS catalog, as well as NIH designated repositories. Tau PET scans, GWAS genotyping data, DNA methylation, and clinical data from the ADNI cohort: http://adni.loni.usc.edu; AMP-AD bulk RNA-Seq data: https://www.synapse.org/#!Synapse:syn17115987; AMP-AD snRNA-Seq data: https://www.synapse.org/#!Synapse:syn31512863; Tg4510 and J20 mouse RNA-Seq data: https://www.epigenomicslab.com/ADmice/; Human Brain microarray datasets: https://human.brain-map.org/static/download.

Research involving human participants, their data, or biological material

Policy information about studies with human participants or human data. See also policy information about sex, gender (identity/presentation), and sexual orientation and race, ethnicity and racism.

Reporting on sex and gender	Self-reported biological sex was included as a covariate in all analyses. In addition, stratified analyses of the main genetic effects were done by self-reported biological sex.
Reporting on race, ethnicity, or other socially relevant groupings	Only non-Hispanic participants of European ancestry by multidimensional scaling analysis using GWAS genotype data were selected for the genetic association analysis.
Population characteristics	Only non-Hispanic participants of European ancestry by multidimensional scaling analysis using GWAS genotype data were selected for the genetic association analysis. Furthermore, two principal component (PC) factors from population stratification were used as covariates to help account for any substructure effects. We note that we consider multi-ethnic studies to be very important but such data are not yet available for tau PET coupled with GWAS. We and others are actively working on collection of such data in ADNI4, KBASE/ADSP, CLEAR-AD and other NIA-sponsored studies.
Recruitment	Participants were recruited from a broad range of academic medical centers and clinical trial sites in the U.S., Canada, and Australia through community advertisement and clinical referral.
Ethics oversight	Indiana University School of Medicine - Institutional Review Board (IBR); IRBs from each institution contributing data

Note that full information on the approval of the study protocol must also be provided in the manuscript.

Field-specific reporting

Please select the one below that is the best fit for your research. If you are not sure, read the appropriate sections before making your selection.

☒ Life sciences ☐ Behavioural & social sciences ☐ Ecological, evolutionary & environmental sciences

For a reference copy of the document with all sections, see nature.com/documents/nr-reporting-summary-flat.pdf

Life sciences study design

All studies must disclose on these points even when the disclosure is negative.

Sample size	During last two years, we collected data from 12 independent cohorts that have both tau PET (positron emission tomography) scans and genome-wide genotype (GWAS array) data. We performed a genome-wide association study of cortical tau quantified by PET in 3,136 participants from 12 independent studies.
Data exclusions	In the analysis, no data were excluded. However, to reduce the possible impact of population stratification, we included by design only non-Hispanic participants of European ancestry based on multidimensional scaling analysis of the GWAS genotype data. These design criteria were to enable the first large-scale tau PET GWAS while avoiding the well-known population stratification effects. Future multi-ethnic tau PET GWAS studies are in the data collection phase and will likely take several more years to reach appropriately well-powered sample sizes.
Replication	For the discovery and replication analysis, we used the same phenotype, cortical tau deposition quantified by positron emission tomography (tau PET) in independent cohorts. In the replication, we replicated a genetic locus that was genome-wide significant in the discovery data set.
Randomization	No randomization was necessary as this study was an association study. Participants were grouped by diagnosis (assessed at sites collecting the data) and genotype for the identified SNP in the replication phase.
Blinding	All diagnostic categorization, data collection, and scan preprocessing and analysis were done blinded to genotype.

Reporting for specific materials, systems and methods

We require information from authors about some types of materials, experimental systems and methods used in many studies. Here, indicate whether each material, system or method listed is relevant to your study. If you are not sure if a list item applies to your research, read the appropriate section before selecting a response.

Materials & experimental systems

n/a	Involved in the study	
☒	☐	Antibodies
☒	☐	Eukaryotic cell lines
☒	☐	Palaeontology and archaeology
☒	☐	Animals and other organisms
☐	☒	Clinical data
☒	☐	Dual use research of concern
☒	☐	Plants

Methods

n/a	Involved in the study	
☒	☐	ChIP-seq
☒	☐	Flow cytometry
☐	☒	MRI-based neuroimaging

Clinical data

Policy information about clinical studies

All manuscripts should comply with the ICMJE guidelines for publication of clinical research and a completed CONSORT checklist must be included with all submissions.

Clinical trial registration: This report is not a clinical trial. The data analyzed here are from observational studies without interventions or random assignment.

Study protocol: Study design characteristics for each of the 12 cohorts included are available in other publications and briefly summarized in this report (supplemental material).

Data collection: Measures used in clinical research are included and reported within the manuscript and supplemental materials.

Outcomes: No clinical outcomes were included as this is an observational genetic association study. Clinically relevant variables were assessed and reported within to facilitate characterization of the cohorts and interpretation of findings.

Plants

Seed stocks: N/A

Novel plant genotypes: N/A

Authentication: N/A

Magnetic resonance imaging

Experimental design

Design type: N/A

Design specifications: N/A

Behavioral performance measures: N/A

Acquisition

Imaging type(s): Structural

Field strength: 3 Tesla

Sequence & imaging parameters: MPRAGE or comparable T1-weighted volumetric scan.

Area of acquisition: Whole brain - used for PET scan processing

Diffusion MRI: ☐ Used ☒ Not used

Preprocessing

Preprocessing software: VBM (voxel-based morphometry) using SPM12.

Normalization: Non-linear transformation of both MRI and PET scans to normal atlas space.

Normalization template: MNI

Noise and artifact removal: Motion correction during generation of static PET images for between frame movement.

Volume censoring: SPM12 - voxel-wise analysis masked for grey matter + white matter

Statistical modeling & inference

Model type and settings: Multivariate linear regression

Effect(s) tested: Genotype; genotype by diagnosis; genotype by amyloid positivity; genotype by APOE e4 positivity; sex effects in stratified analysis

Specify type of analysis: ☐ Whole brain ☐ ROI-based ☒ Both

Anatomical location(s): Whole cerebral cortex - sum of all Freesurfer v6 parcellation regions; medial temporal lobe - sum of entorhinal cortex, parahippocampal gyrus,

Statistic type for inference: voxel-wise $p<0.05$

(See Eklund et al. 2016)

Correction: FWE

Models & analysis

n/a	Involved in the study
☒	☐ Functional and/or effective connectivity
☒	☐ Graph analysis
☐	☒ Multivariate modeling or predictive analysis

Multivariate modeling and predictive analysis: Effect of genotype on voxel-wise and ROI-based tau deposition from PET covaried for age, sex, APOE e4 carrier status, first two genetic ancestry principal components, amyloid positivity, and diagnosis
